# Active and stable alcohol dehydrogenase-assembled hydrogels via synergistic bridging of triazoles and metal ions

**DOI:** 10.1038/s41467-023-37921-y

**Published:** 2023-04-13

**Authors:** Qiang Chen, Ge Qu, Xu Li, Mingjian Feng, Fan Yang, Yanjie Li, Jincheng Li, Feifei Tong, Shiyi Song, Yujun Wang, Zhoutong Sun, Guangsheng Luo

**Affiliations:** 1 The State Key Lab of Chemical Engineering, Department of Chemical Engineering, Tsinghua University, Beijing, 100084 China; 2 Tianjin Institute of Industrial Biotechnology, Chinese Academy of Sciences, Tianjin, 300308 China; 3 Technology Center for Protein Sciences, School of Life Sciences, Tsinghua University, Beijing, 100084 China

**Keywords:** Biocatalysis, Cryoelectron microscopy, Immobilized enzymes

## Abstract

Biocatalysis is increasingly replacing traditional methods of manufacturing fine chemicals due to its green, mild, and highly selective nature, but biocatalysts, such as enzymes, are generally costly, fragile, and difficult to recycle. Immobilization provides protection for the enzyme and enables its convenient reuse, which makes immobilized enzymes promising heterogeneous biocatalysts; however, their industrial applications are limited by the low specific activity and poor stability. Herein, we report a feasible strategy utilizing the synergistic bridging of triazoles and metal ions to induce the formation of porous enzyme-assembled hydrogels with increased activity. The catalytic efficiency of the prepared enzyme-assembled hydrogels toward acetophenone reduction is 6.3 times higher than that of the free enzyme, and the reusability is confirmed by the high residual catalytic activity after 12 cycles of use. A near-atomic resolution (2.1 Å) structure of the hydrogel enzyme is successfully analyzed via cryogenic electron microscopy, which indicates a structure–property relationship for the enhanced performance. In addition, the possible mechanism of gel formation is elucidated, revealing the indispensability of triazoles and metal ions, which guides the use of two other enzymes to prepare enzyme-assembled hydrogels capable of good reusability. The described strategy can pave the way for the development of practical catalytic biomaterials and immobilized biocatalysts.

## Abstract (web summary)

Biocatalysis using immobilized enzymes is increasingly replacing traditional methods of manufacturing fine chemicals but heir industrial applications are limited by the low specific activity and poor stability. Here, the authors report a feasible strategy utilizing the synergistic bridging of triazoles and metal ions to induce the formation of porous enzyme-assembled hydrogels with increased activity.

## Introduction

Biocatalytic transformation has emerged as a key industrial technology for the sustainable manufacture of high-value chemicals with excellent selectivity (particularly stereoselectivity), low environmental footprint, and cost-effectiveness, which enable its potential application as a viable alternative to traditional processes^1,2^. In the pharmaceutical manufacturing, enzymatic catalysis exhibits many advantages and high constructability, playing an irreplaceable role in the industrial synthesis of a broad range of important chiral compounds^3,4^. Enzymes, which are the workhorses of biocatalytic processes, can be tailored to specific requirements using various approaches, such as directed evolution and rational design^5,6^. However, the tertiary enzyme structure may undergo denaturation owing to its flexible nature^7^, which considerably limits the industrial application of enzymes and decreases their long-term stability. Moreover, most enzymes are water-soluble homogeneous catalysts whose recovery requires the use of expensive ultrafiltration systems^8^; therefore, they are usually discarded after a single use, which contradicts the principles of circular economy^9^.

The use of protein immobilization technology can effectively overcome the aforementioned challenges. As a heterogeneous biocatalyst, an immobilized catalyst can be easily recovered and separated from the product via simple filtration or centrifugation, and its immobilized state is conducive to stabilizing the tertiary structure of the protein^10^. In recent decades, various protocols have been developed for immobilizing enzyme^11,12^, which have even boosted the rise of flow biocatalysis^13–15^; however, it is still difficult to achieve an appropriate balance between catalytic activity and stability during immobilization. Therefore, methods for mitigating the above-mentioned issues are currently being explored^16,17^. Most schemes involve bonding biomacromolecules to host carriers either through strong interactions (such as covalent bonds^18,19^) or weak interactions (such as physical adsorption)^20,21^. However, strong interaction often lead to a loss of activity or even complete inactivation, while weak interactions are characterized by low stability. In addition, the use of frameworks for physical encapsulation typically results in high mass-transfer resistance, which requires the utilization of complex surface engineering techniques^22,23^. Note that while the use of carriers incurs additional costs, the loading amount of the enzyme is severely limited by the surface area of the matrix, thereby decreasing the specific activity of the prepared bio-composite. Meanwhile, the carrier-free immobilization based on strong intermolecular interactions is cost-effective and leads to high specific activity. However, the strategy of self-immobilizing enzyme fusion relies on the complex de novo coding of genes^24,25^. Moreover, the use of cross-linking agents to induce the formation of cross-linked enzyme aggregates results in high activity losses^26^. To the best of our knowledge, a carrier-free immobilization method based on weak interactions has not been reported thus far. If the instability issue caused by weak interactions can be effectively mitigated, such a method can potentially combine cost-effectiveness, high carrier-free specific activity, and low activity loss under the action of weak forces.

**Fig. 1:**
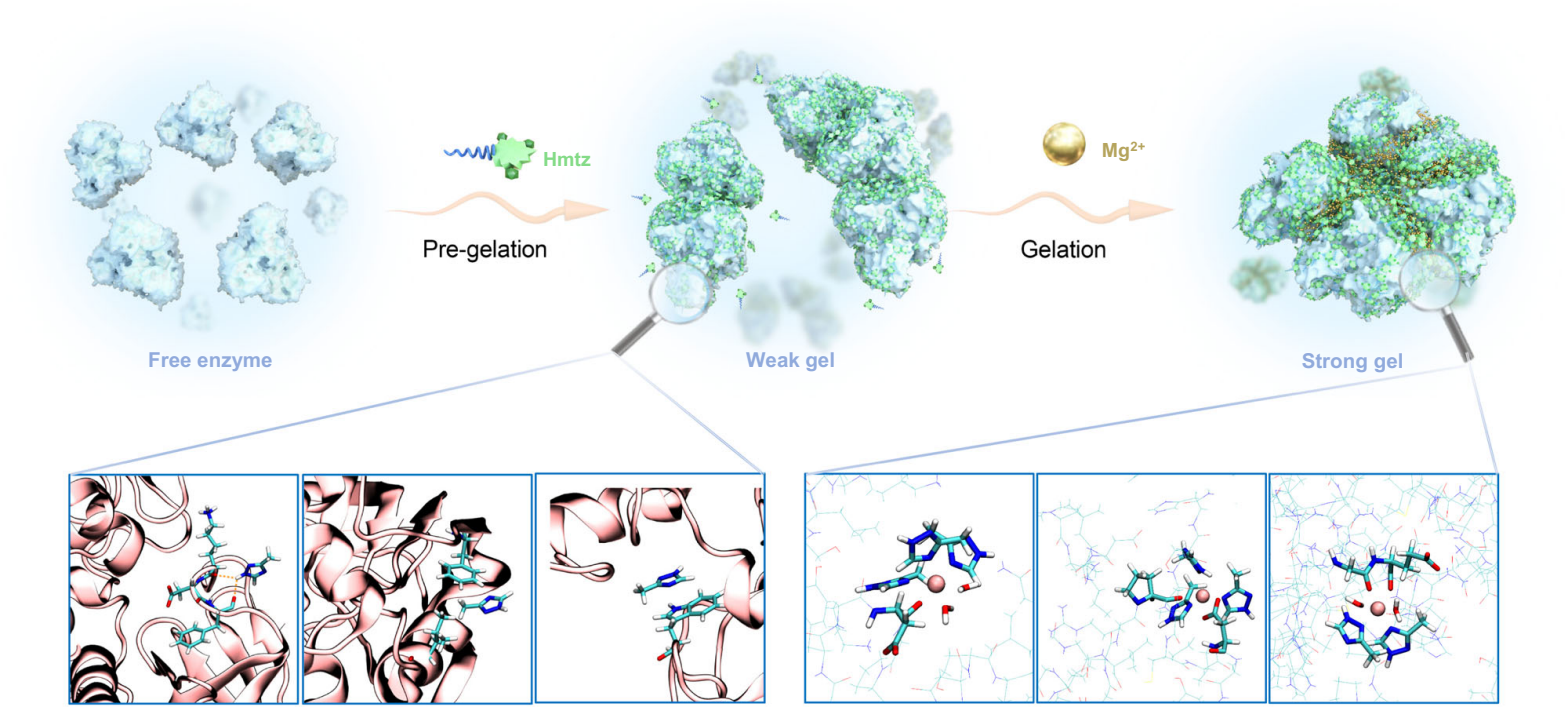
Schematic of EAG preparation. 1H-3-methyl-1,2,4-triazole (Hmtz) and Mg^2+^ ions were added to the enzyme solution before the pre-gelation and gelation processes, respectively. The left three local structures show some potential interaction sites of Hmtz on the protein surface; the right three local structures show the possible coordination spheres of Mg^2+^ at the bridging interface.

In this study, we developed a carrier-free immobilization method based on the weak force-coupled multisite bridging of enzyme molecules (Fig. 1). First, the binding of triazole molecules to alcohol dehydrogenase through hydrogen bonding and hydrophobic interactions induced a weak aggregation of enzyme molecules (pre-gelation). Afterward, the introduced magnesium ions (Mg^2+^) underwent polydentate chelation with triazoles and enzyme surface groups that increased the stability of the bridging interface and promoted further gelation. This strategy resulted in the formation of enzyme-assembled hydrogels (EAGs) with hierarchical porosity, which were used to catalyze the asymmetric reduction of aryl prochiral ketones with considerably higher activity than that of free enzymes and good reusability. Moreover, EAGs exhibited enhanced stability under various unfavorable conditions, successfully realizing the coexistence of various potentially advantageous properties of immobilized enzymes. Using cryogenic electron microscopy (Cryo-EM), a structure–property relationship of the enzyme after gelation was analyzed for the improved catalytic performance, and a near-atomic-level enzyme structure with an ultra-high resolution of 2.1 Å was obtained. Molecular dynamics (MD) simulations were performed to further investigate the enhanced catalytic efficiency and robustness of the enzymes. Moreover, the possible mechanism of EAG formation was elucidated by conducting both simulations and experiments, and the synergistic effect of triazoles and Mg^2+^ ions on the formation of bridging interfaces between enzymes was demonstrated. Further, the gelation immobilizations of two other alcohol dehydrogenases with different structures were successfully achieved, and the reusability of the produced EAGs was tested. By successfully synthesizing a carrier-free immobilized biocatalytic material with high activity and stability, this study provides a strategy for the development of catalytic biomaterials and immobilization methods.

## Results

### Preparation and catalytic performance of EAG

**Fig. 2:**
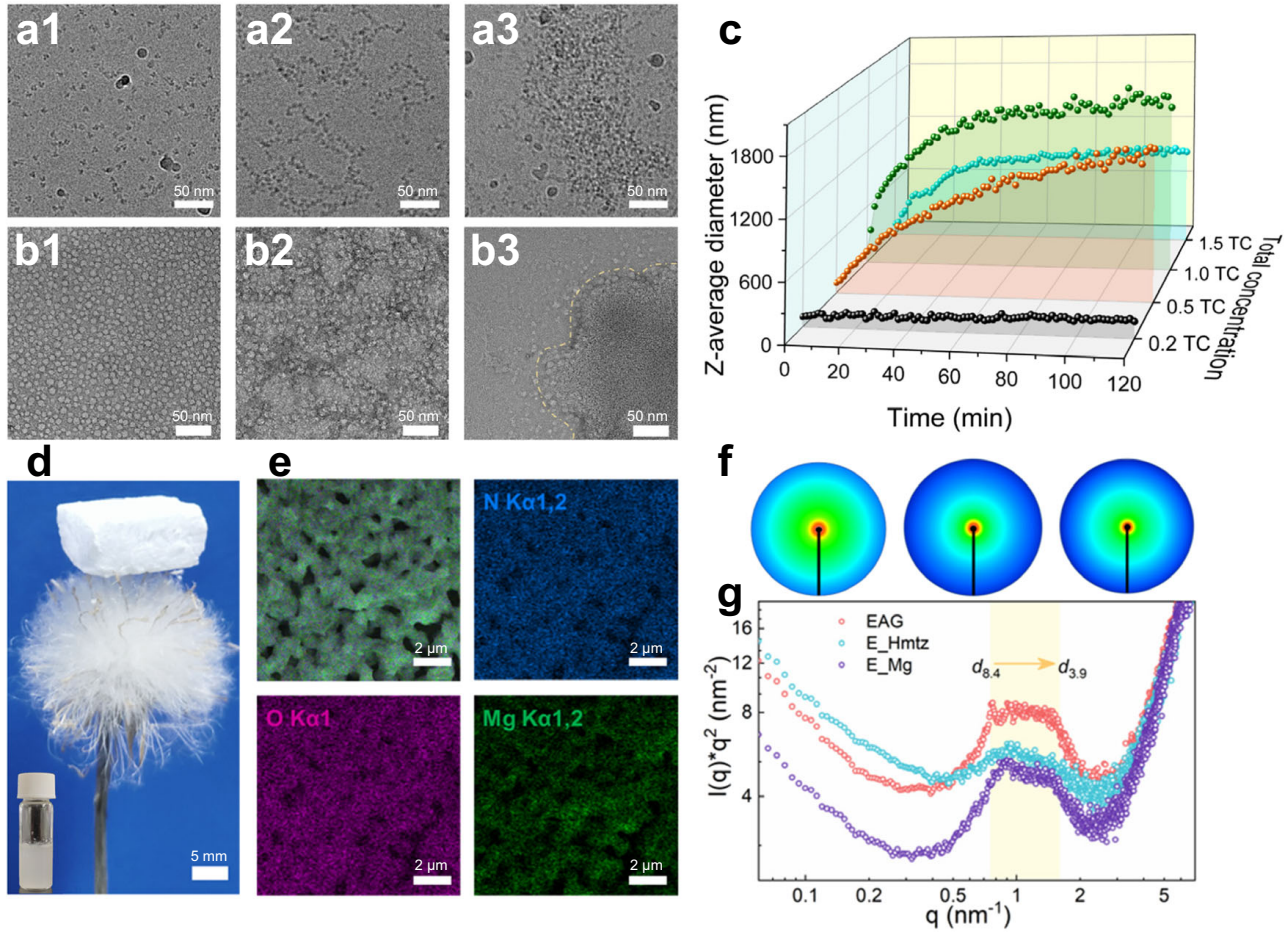
Characterization of EAG. **a** Cryo-TEM and **b** negatively stained TEM images of the free enzyme obtained (1) before pre-gelation, (2) after pre-gelation, and (3) after gelation. Both the pre-gelation and gelation processes were terminated at 2 min. Black particles in (**a**) and white particles in (**b**) represent enzyme molecules. For (**a**) and (**b**), representative images from five independent experiments are shown. In (**c**), In situ DLS assays obtained during the pre-gelation process at different enzyme concentrations and a temperature of 25 °C. Notation 1.0 TC refers to the enzyme concentration of 1 mg/mL, and the Hmtz:E mass ratio is equal to 60. **d** Photograph of the lyophilized EAG on top of a dandelion. Inserted image is the EAG suspension in a 4 mL vial before lyophilization. **e** SEM and EDX mapping images of EAG. Representative images from three independent experiments are shown. **f** SAXS two-dimensional (2D) patterns of E_Mg (left), E_Hmtz (middle), and EAG (right). **g** SAXS q^2^I(q) representations of E_Mg, E_Hmtz, and EAG. Source data are provided as a Source data file.

The obtained experimental data revealed that triazoles induced the aggregation of *Thermoanaerobacter brockii* alcohol dehydrogenase (TbSADH, Supplementary Fig. 1) to form a gel complex; however, its stability was very low. Intermolecular interactions in this gel were strengthened by the synergistic chelation of metal ions. As shown in Fig. 1, a stable EAG (Fig. 2d) was prepared by successively adding Hmtz and Mg^2+^ to the TbSADH solution, as described in the methods section. Both Fourier-transform infrared (FTIR) and Raman spectra confirmed the presence of the characteristic C–N–N in-plane bending of Hmtz and the enzyme amide bonds in the prepared gel complex (Supplementary Figs. 2 and 3). High-resolution cryogenic transmission electron microscopy (cryo-TEM) was performed to study the microscopic aggregation of TbSADH in an aqueous solution. The pre-gelation of Hmtz induced weak enzyme aggregation (Fig. 2a2), while the gelation of Hmtz-Mg^2+^ synergistically induced a strong aggregation process (Fig. 2a3). Consistently, the negative-stain TEM characterization of lyophilized EAG further demonstrated the existence of weak and strong aggregation states induced by Hmtz and the Hmtz–Mg^2+^, respectively (Fig. 2b). To determine the factors affecting the kinetics of TbSADH (E) aggregation, in situ dynamic light scattering (DLS) analysis was performed to measure the hydraulic diameter of the enzyme–Hmtz complex^27^. The conditions Hmtz:E = 60:1 (mass ratio) and 20–25 °C were found to be most favorable for the gelation process (Supplementary Fig. 4). When the Hmtz:E ratio was very low (<10), no gel was formed, which was consistent with the observed change in the zeta potential on the enzyme surface with the Hmtz:E ratio (Supplementary Fig. 5). As shown in Fig. 2c, the aggregate size in the pre-gelation process increased with time and remained constant after 2 h. The growth rate was affected by the enzyme concentration, and in particular, a gel could not be produced at enzyme concentrations below 0.2 mg/mL.

After the synergistic induction of Hmtz and Mg^2+^ ions, enzyme molecules continuously assembled to form a hydrogel with a hierarchically porous architecture. The upturn in I(q) in the low q range was confirmed by the results of small angle X-ray scattering (SAXS) experiments (Supplementary Fig. 6), which was characteristic of hydrogel heterogeity^28^. The porous structure (Fig. 2e and Supplementary Figs. 7 and 8) of EAG increased its potential applicability as an industrial heterogeneous biocatalyst. As controls, the denaturation complex of TbSADH induced by Mg^2+^ at high concentrations (E_Mg) and weak gel of the Hmtz–TbSADH complex (E_Hmtz) were used. As shown in Fig. 2f, the high-value halo size of the equatorial center indicating the degree of intermolecular compactness increases in the order E_Mg < E_Hmtz < EAG. Molecular interplanar distances between 8.4 and 3.9 nm were detected in EAG, and within this range, EAG exhibited a larger number of interplanar short distances than those obtained for E_Mg and E_Hmtz (Fig. 2g), implying the synergetic effect of Hmtz and Mg^2+^ on the gelation process. The Scanning electron microscopy (SEM) images of EAG and the corresponding energy-dispersive X-ray (EDX) spectroscopy mapping (Fig. 2e) showed that the signals of N, O, and Mg largely overlapped with the porous backbone, and although Hmtz contributed part of the N signal, the high signal intensities of N and O were mainly from the protein contribution, while the EDX signal of Mg proved its distribution in the porous backbone. Moreover, the negative-stained TEM and its EDX mapping (Supplementary Fig. 9) similarly demonstrated the high protein content and the presence of Mg distribution in the porous backbone of EAG. To statistically estimate specific pore information, SEM images were analyzed thus revealing that the lyophilized EAG contained macropores with an average pore size of 406 nm (Supplementary Fig. 7c), which is consistent with the macropore size of EAG in solution (see below). To examine the characteristics of mesopores, N_2_ isothermal adsorption–desorption experiments were performed (Supplementary Fig. 10). Their results confirmed the presence of mesopores with an average size of 8.3 nm in the EAG structure, and its mesopore content was significantly higher than those of E_Mg and E_Hmtz. In summary, EAG exhibited a hierarchical pore structure with pore sizes ranging from 3 to 900 nm, which was beneficial for the mass transfer process during the reaction. However, it is worth noting that excessive pore lengths in EAG may increase the mass transfer resistance. Therefore, the EAG used for the reaction is preferably dispersed as small-sized gel particles, while avoiding direct use of bulk gels.

**Fig. 3:**
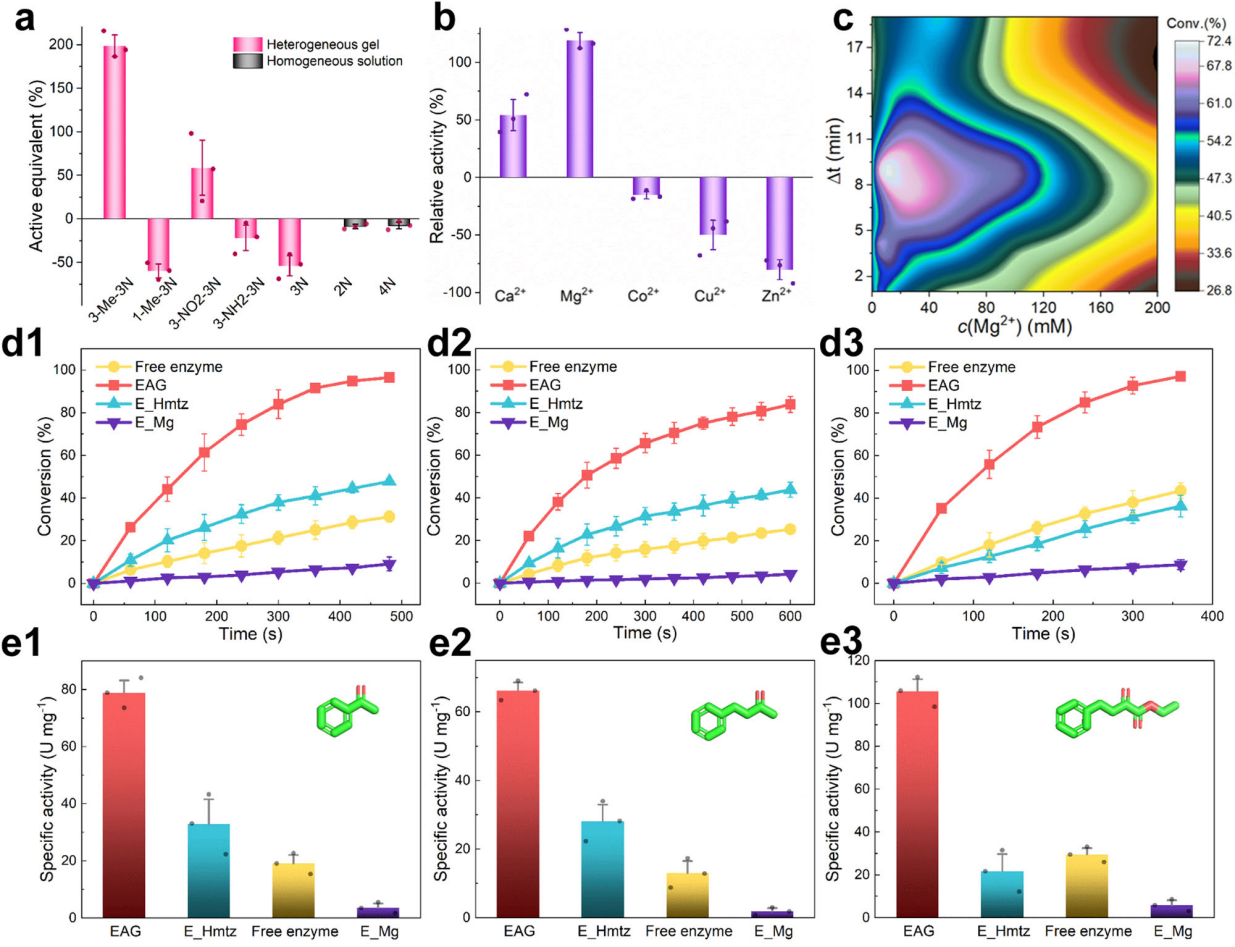
Catalytic performance of EAG for the asymmetric reduction of prochiral aryl ketones. **a** Screening of polyazoles based on the active equivalent of the formed gel (red bars) or solution (black bars) compared to the catalytic activity of the free enzyme utilized for gelation. 3-Me-3N: Hmtz. 1-Me-3N: 1-methyl-1,2,4-triazole (without N–H). 3-NO2-3N: 3-nitro-1,2,4-triazole. 3-NH2-3N: 3-amino-1,2,4-triazole. 3N: 1,2,4-triazole. 2N: imidazole. 4N: tetrazole. **b** Screening of metal ions based on the catalytic activity of the formed gel relative to that of the enzyme without metal ions (0%). **c** Effects of the Mg^2+^ concentration (*c*(Mg^2+^)) and pre-gelation time (Δ*t*) on the conversion at 4 min (Supplementary Table 2). **d** Conversion plots and **e** specific activity values obtained for the asymmetric reduction of (**d1**, **e1**) acetophenone (AP), (**d2**, **e2**) benzyl acetone (BAT), and (**d3**, **e3**) ethyl 2-oxo-4-phenylbutyrate (EBP) by different catalysts. Specific activity was calculated using the conversion at 1 min. U refers to the activity unit expressed as micromoles of substrate converted per hour. Unless specified otherwise, the reduction of 5 mM AP at 30 °C for 10 min was used as the template reaction for evaluating catalytic performance. For (**a**, **b**) and (**d**, **e**), *n* = 3. Error bars represent the standard deviations from three independent experiments; data are expressed as the mean ± SEM. Source data are provided as a Source data file.

The EAG synthesis conditions were optimized using the asymmetric reduction of AP as a template reaction. The utilized triazole type affected the amount of the produced gel (Fig. 3a). For triazoles with different substitutions, 3-methyl or 3-nitro substituted triazoles resulted in large gel amounts with activity equivalents even higher than that of the original enzyme. In contrast, 3-amino and 1-methyl substituted and unsubstituted triazoles produced low-quality gels with activity equivalents lower than that of the offered enzyme. However, no gel was formed for imidazole or tetrazole. These results suggest that Hmtz produced a stronger effect on the gelation of TbSADH, which may be attributed to its N–H and hydrophobic –CH_3_ groups. In addition, the effects of Ca^2+^, Mg^2+^, Co^2+^, Cu^2+^, and Zn^2+^ ions on the activity of the formed gel were examined. The obtained results revealed that the gel containing Mg^2+^ ions possessed the highest activity, while Co^2+^, Cu^2+^, and Zn^2+^ ions had negative effects on the gel activity (Fig. 3b). The catalytic activity corresponding to Zn^2+^ was the lowest, probably because on the one hand, the strong coordination between Zn^2+^ and triazoles led to a tight cross-linking of enzymes and reduced activity, and on the other hand, the Zn^2+^ in the cross-linked networks on the enzyme surface increased the diffusion resistance of the substrates into the active center by trapping the substrates. Furthermore, the effects of the *c*(Mg^2+^) and Δ*t* on the EAG activity were explored. As shown in Fig. 3c, the optimal *c*(Mg^2+^) value was 10–15 mM, and the optimal Δ*t* magnitude was equal to 8–10 min. Under this optimal condition, the immobilization yield of the enzyme for EAG synthesis was determined to be 53.7%.

Significantly, this three-dimensional (3D) porous EAG exhibited high catalytic activity and stability during the conversion of several aryl prochiral ketones, especially compared with the most common adsorption method based on commercial activated carbon carriers for enzyme immobilization reported in the literature^29^, which had a specific activity in the order of 0.1 U mg^−1^ and only 10% activity retention after 6 reuse cycles. As shown in Fig. 3d, EAG smoothly catalyzed the reactions and achieved a 96.5% conversion for AP within 8 min, 83.8% conversion for BAT within 10 min, and 97.2% conversion for EBP within 6 min. At a lower enzyme concentration of 0.2 mg/mL, the catalytic performance of EAG was still significantly enhanced compared to that of the free enzyme (Supplementary Fig. 11). Chiral high-performance liquid chromatography (HPLC) and ^1^H and ^13^C nuclear magnetic resonance (NMR) spectra confirmed that the chiral selectivity of the reaction exceeded 99%, while the conversions of AP, BAT, and EBP generated the corresponding nearly optically pure *R*, *S*, and *R* alcohols, respectively (Supplementary Figs. 12 and 13). In comparison, E_Hmtz, the free enzyme, and E_Mg exhibited significantly lower catalytic rates. Furthermore, the apparent specific activities of EAG catalyzing the conversions of AP, BAT, and EBP were equal to 78.9, 66.2, and 106.0 U mg^−1^, which were 4.1, 5.1, and 3.6 times higher than that of the free enzyme, respectively (Fig. 3e). Moreover, the conversion of AP was kinetically tested as a template reaction, and the EAG catalysis demonstrated significantly faster intrinsic kinetics than those of the free-enzyme catalysis (Supplementary Figs. 14 and 15). The fitting of the obtained data using the Michaelis–Menten equation revealed that the *k*_cat_ of EAG was 15.45 s^−1^ (Supplementary Table 1), which was 6.3 times higher than that of the free enzyme (2.46 s^−1^), while the *K*_m_ value increased only slightly from 17.86 to 19.84 mM, indicating that the catalytic efficiency of TbSADH was considerably improved after gelation and that the intrinsic catalytic activity (*k*_cat_/*K*_m_) obtained for the AP substrate increased by 5.6 times.

**Fig. 4:**
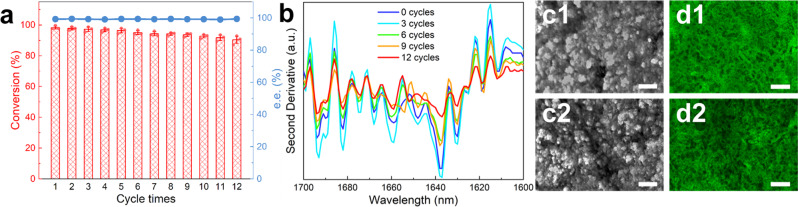
Reusability of EAG. **a** Conversions and enantiomeric excess (e.e.) values measured during 12 reaction cycles using EAG as the catalyst. *n* = 3. Data are expressed as the mean ± SEM. **b** Second derivatives of the FTIR spectra of EAG obtained after different numbers of cycles. **c** TEM images of the lyophilized EAG and **d** Confocal laser scanning microscope (CLSM) images of EAG in solution obtained (**c1**, **d1**) before use and (**c2**, **d2**) after 12 cycles. Scale bars: **c** 200 nm and **d** 4 μm. Error bars represent the standard deviations from three independent experiments. Source data are provided as a Source data file.

Significantly, the EAG heterogeneous catalyst can be recovered via simple filtration or centrifugation. The results of stability tests showed that EAG retained a high activity of 90.3% after 12 reuse cycles, while the enantioselectivity remained above 99%, indicating the good reusability of the hydrogel biocatalyst (Fig. 4a). As shown in Fig. 4b and Supplementary Fig. 16, the FTIR spectra and their second derivatives of EAG after multiple repeated uses were essentially the same compared to the peaks before use, indicating that the secondary structure of the EAG enzyme was well preserved^21^. In addition, the TEM images of lyophilized EAG and CLSM images of EAG in aqueous solution both confirmed that the porous architecture of EAG remained intact after multiple use cycles (Fig. 4c and d). Notably, the gel state enhanced the rigidity of the protein and suppressed the unfolding of the tertiary structure, rendering it more tolerant to organic solvents, heat treatment, proteolysis, and storage than the free enzyme (Supplementary Figs. 17 and 18, and also see below). Experimental data revealed that the free enzyme retained only 41.3% and 32.8% of its original activity after the exposure to isopropanol (IPA) and acetonitrile (ACN); however, EAG maintained 68.6% and 61.8% of its original activity, respectively (Supplementary Fig. 17). Moreover, the EAG enzyme suffered less substrate inhibition compared to the free state (Supplementary Fig. 19), which is beneficial for their industrial applications. Furthermore, the activity retention of EAG after 30 d of storage was as high as 92.5%, which was considerably larger than that of the free enzyme (55.8%) (Supplementary Fig. 18a). Note that in the presence of trypsin (3 mg mL^−1^ for 3 h at 25 °C), EAG retained 74.6% of its original activity compared to the value of 15.3% determined for the free enzyme (Supplementary Fig. 18b). In addition, the melting temperature (*T*_m_) of EAG (10 mM Mg^2+^) measured using differential scanning calorimetry (DSC) was 133.8 °C, which was 35.2 °C higher than that of the free enzyme (see below), indicating that the robustness of EAG was significantly improved with respect to that of the free enzyme. Moreover, EAG has sufficient mechanical stability and can be used well in stirred reactors (Supplementary Fig. 20). As an immobilized biocatalyst, EAG exhibits significant potential for adaptation to highly demanding application scenarios.

### Shedding light on the origin of the enhanced catalytic performance

Regarding the immobilization methods of enzymes, the examination of structural changes of enzymes after immobilization has always been the focus of attention and also a challenging difficulty^30,31^. The structure of EAG can be classified into two categories, one is the main structure of the enzyme (EAG enzyme structure) and the other is the structure of the network interface between the enzymes (enzyme-Hmtz-Mg structure). Since the binding of Hmtz to proteins is non-specific, the structure of the formed enzyme-Hmtz-Mg network is impossible to be resolved by cryo-EM or X-ray crystallography. However, the EAG enzyme structure, which contains the active center and the main structure of the enzyme after immobilization, is possible to be resolved, although high-resolution structures of such small molecular weight protein (<200 kDa) remain still difficult^32^.

**Fig. 5:**
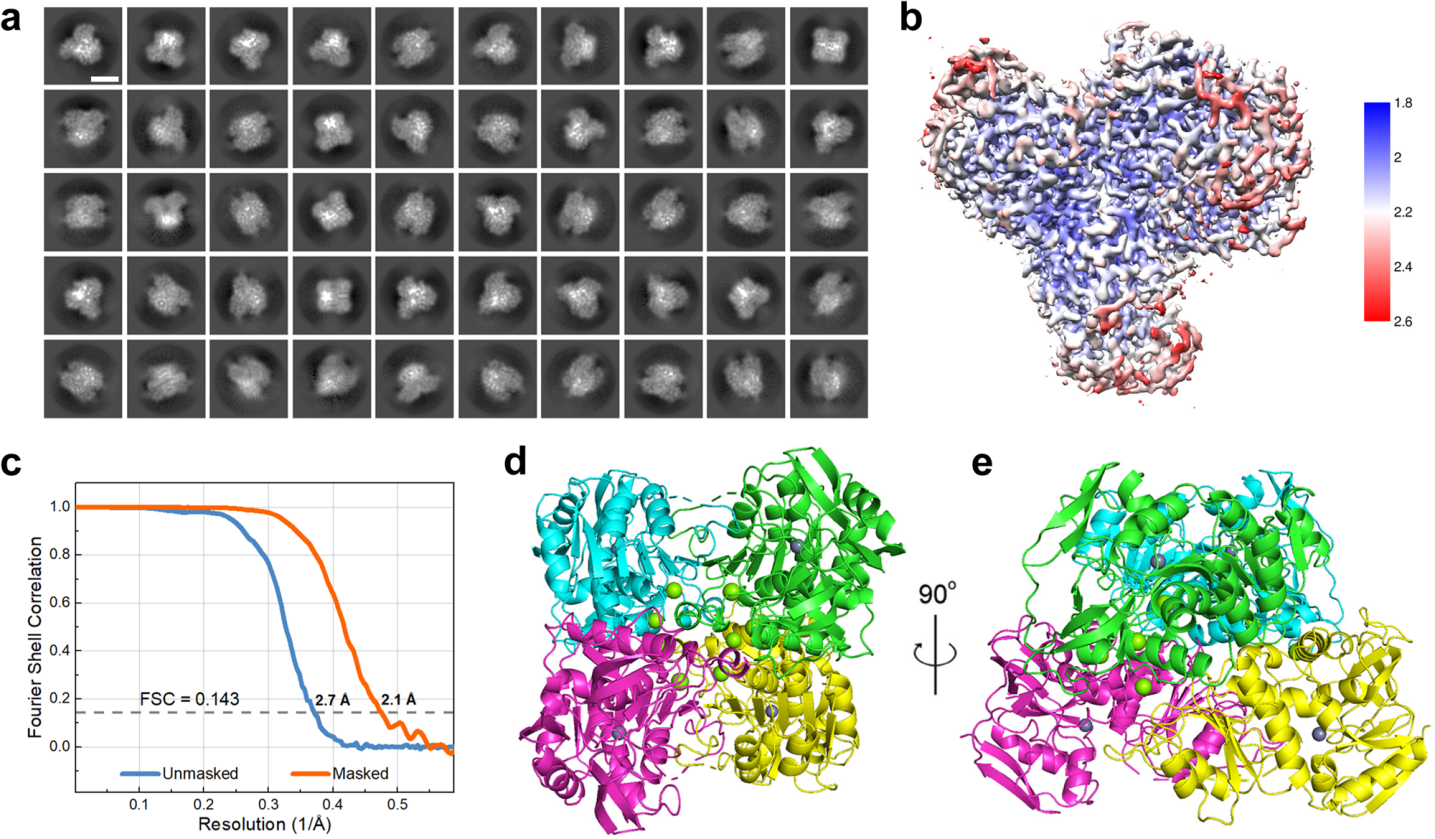
Cryo-EM structural determination of the EAG enzyme. **a** Representative 2D class average of enzyme particle images. Scale bar, 5 nm. **b** A D2 density map with a near-atomic resolution. Map is colored according to the local resolution values estimated using RELION. Color key on the right shows the local structural resolution expressed in angstroms (Å). **c** Fourier shell correlation (FSC) curves obtained for the masked (orange) and unmasked (blue) maps with the gold-standard refinement used for the final resolution determination. Global final map resolution of 2.1 Å was calculated at an FSC cutoff of 0.143. **d**, **e** Overall enzyme structure viewed from different sides. Green and gray beads represent Mg^2+^ and Zn^2+^ ions, respectively. Subunits are displayed in the cartoon models with different colors. PDB code: 7XY9.

EAG enzyme structure was analyzed using a Titan Krios cryo-EM (Supplementary Table 3). A total of 8299 movies were collected, and 1,254,312 particles were finally selected for data processing. Based on the obtained 2D class averages (Fig. 5a), a 3D density map with high local resolutions was constructed (Supplementary Fig. 21 and Fig. 5b). The overall final resolution of this map was 2.1 Å (Fig. 5c). To investigate the structural changes of TbSADH after gelation, an atomic model within the cryo-EM map (PDB code 7XY9) was built based on the available crystal structure of TbSADH (PDB code 1YKF), as shown in Fig. 5d and 5e. The obtained density map matched the atomic model very precisely (Supplementary Fig. 22). In this model, the tetrameric enzyme has a symmetrical tetrahedral structure with one Zn^2+^ active center in each subunit, and no Hmtz was found in the structure, suggesting that Hmtz may bind to the enzyme by a non-specific manner. The active center catalyzing the asymmetric reduction of aryl prochiral ketones is mainly composed of Zn^2+^ ions and three amino acid residues (H59, C37, and D150) (Supplementary Fig. 23). According to the structure of the active center (Supplementary Fig. 23c), Zn^2+^ was not bound by triazole, indicating that the triazoles acted on the enzyme surface without entering the active center to scavenge Zn^2+^. The high-resolution cryo-EM structure provided a solid basis for the analysis of structure–performance relationship.

**Fig. 6:**
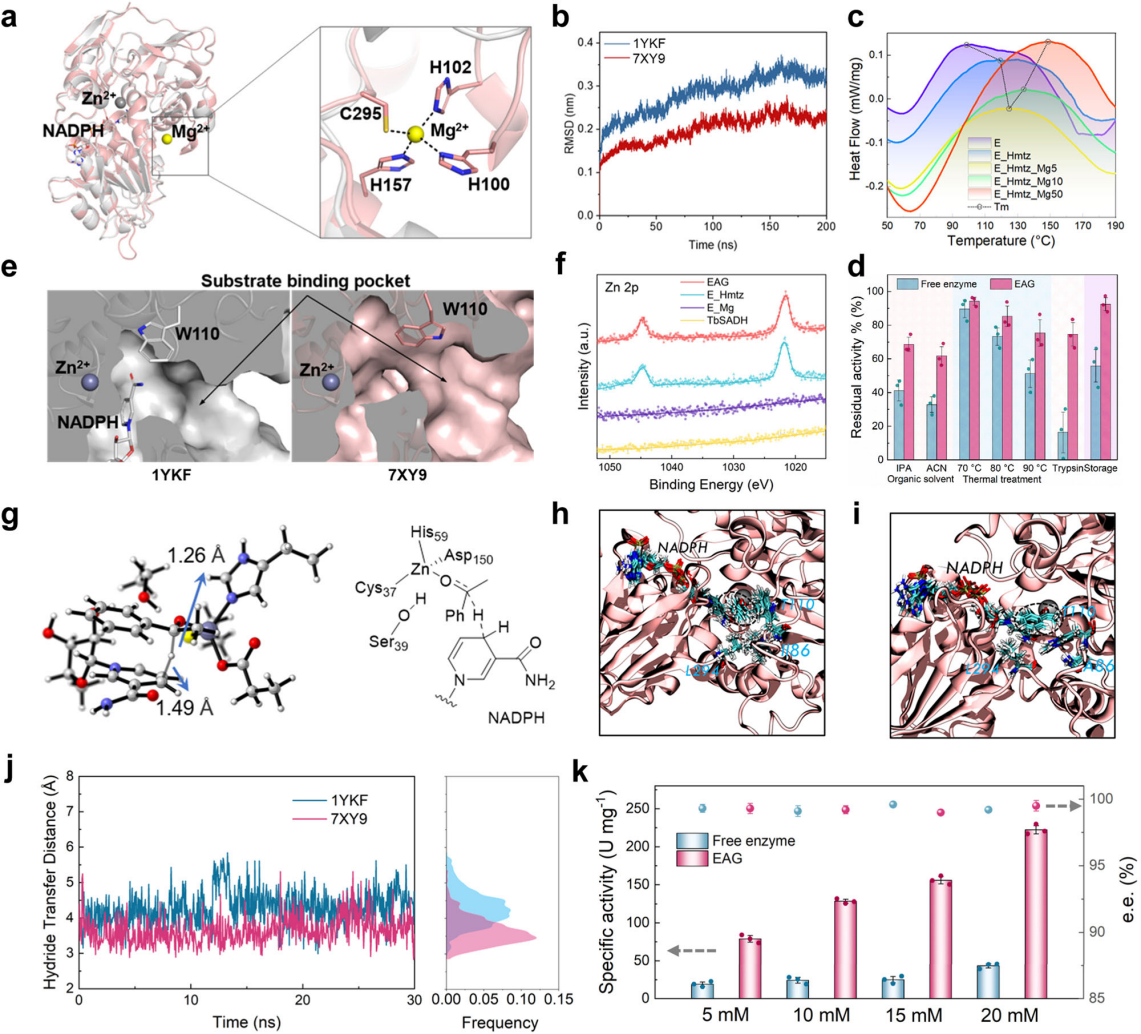
Structural analysis of the differences in enzyme activity and stability before (1YKF) and after (7XY9) gelation. **a** Overlay of the 7XY9 (salmon) and 1YKF (gray) structures. Inset shows the coordination of the Mg^2+^ ion with the adjacent residues. Dashed lines indicate the distances between the Mg^2+^ ion and coordination atoms of H102, C295, H157, and H100, which are equal to 2.2, 2.3, 2.0, and 2.4 Å, respectively. **b** RMSD values of 1YKF and 7XY9 obtained via high-temperature simulations. **c** DSC curves showing the thermostability of E, E_Hmtz, and EAG synthesized at different *c*(Mg^2+^) values. **d** Residual activities of the free enzyme and EAG measured after the exposure to an organic solvent (30% IPA or ACN, 2 h), thermal treatment (1 h), proteolytic agent treatment (trypsin, 3 h), and storage for 30 d. **e** Sliced surfaces of 1YKF (left) and 7XY9 (right). Black arrows indicate the substrate-binding pockets. **f** High-resolution Zn 2p XPS profiles of TbSADH, E_Mg, E_Hmtz, and EAG. **g** TS state determined for the hydride transfer step via DFT calculations. Representative snapshots of the conformational states obtained for **h** 1YKF and **i** 7XY9 via MD simulations. **j** Hydride transfer distance during MD simulations. **k** Specific activity and chiral selectivity values of the free enzyme and EAG catalyzing the conversion of AP with concentrations of 5–20 mM. Error bars represent the standard deviations from three independent experiments. For (**d**) and (**k**), *n* = 3. Data are expressed as the mean ± SEM. Source data are provided as a Source data file.

The cryo-EM structure of EAG enzyme was determined to identify the reason for the increased robustness of the bulk structure. Each of the four chains of 7XY9 shows magnesium ion binding and lacks NADP compared to the wild-type structure 1YKF (Fig. 6a). The structural alignment of 7XY9 to 1YKF results in a root-mean-squared deviation (RMSD) of 0.44 Å (297 core Cα atoms). The large motion of the loop consisting of residues L107 to H100 with a 4.1 Å shift at residue L107 and 10.5 Å shift at residue H100 (Supplementary Fig. 24) leads to a major structural rearrangement of the mutant. This causes the coordination of H100, H102, H157, and C295 to the Mg^2+^ ion (Fig. 6a), which increases the tension and stability of the enzyme structure, thereby enhancing enzyme robustness.

Further, MD simulations were performed to examine the molecular processes responsible for the increased robustness of EAG enzyme. After conducting a 200 ns-long equilibrium simulation at 298.15 K, the system temperature was switched to 373.15 K, and additional simulations were performed for another 200 ns. The low RMSD and per-residue root-mean-square fluctuations of 7XY9 indicate that the incorporations of Mg^2+^ ions in the enzyme bulk structure increase the stability at high temperatures (Fig. 6b and Supplementary Fig. 26). In addition to the specific coordination of Mg^2+^ in the enzyme bulk structure, the network of protein interfaces formed with the participation of Mg^2+^ also improves the robustness of EAG. The results of DSC analysis demonstrated a significant increase in *T*_m_ after gelation (Fig. 6c). Subsequently, the *T*_m_ of EAG continued to increase with increasing *c*(Mg^2+^), confirming the enhanced robustness due to Mg^2+^ coordination. However, the increase in rigidity limits the catalytic activity of the enzyme; therefore, *c*(Mg^2+^) must be accurately determined by evaluating the EAG activity and stability. As shown in Fig. 3c, at Δ*t* = 10 min, *c*(Mg^2+^) increased from 10 to 50 mM, decreasing the relative activity of the prepared EAG by 11.2%. In addition, EAG exhibited higher resistance to various adverse microenvironments than the free enzyme (Fig. 6d). Noteworthy, EAG showed good tolerance to different buffer salt conditions and was able to tolerate solutions with pH determined to be 5.8–11 (Supplementary Fig. 27).

Moreover, the cryo-EM structure of EAG enzyme provides a structural perspective for its activity enhancement. The loop motion further induced the side chain flipping of residue W110 with a torsion angle of 94.5° (Supplementary Fig. 25), thereby expanding the substrate-binding pocket of the enzyme (Fig. 6e). The larger binding pocket may promote the conformational sampling of the substrate and increase the frequency of productive binding modes^33^, thereby increasing the catalytic efficiency of the enzyme. This is consistent with the increased *k*_cat_ value obtained using a kinetic analysis (Supplementary Table 1). Furthermore, the X-ray photoelectron spectroscopy (XPS) data presented in Fig. 6f and Supplementary Fig. 28 indicate the absence of Zn 2p peaks in the TbSADH and E_Mg spectra; however, strong Zn 2p peaks were obtained for E_Hmtz and EAG, suggesting that the gelation process facilitated the exposure of the active center, which was beneficial for the catalytic process.

The activity of TbSADH is associated with the rate of hydride transfer from NADPH to the substrate^34^. Differences in activity can be predicted by estimating the distance between the carbonyl group of the substrate and the NADPH carbon atom involved in hydride transfer. Hence, we computed a transition state (TS) for the hydride transfer to the AP substrate by conducting density functional theory (DFT) calculations on a small subset of enzyme active site residues (Fig. 6g). The obtained TS exhibited a hydride transfer distance of 2.7 Å. To identify the origin of the high activity of 7XY9, we calculated the hydride transfer distances of the AP-bound states of 1YKF and 7XY9 by performing MD simulations (Fig. 6h and i). A constant force was applied to maintain the substrate bound to the Zn^2+^ ion. The MD simulation data revealed that the rearrangement of the substrate-binding pockets facilitated the hydride transfer process because the hydride transfer distance in 7XY9 was shorter than that in 1YKF (Fig. 6j). The enhanced hydride transfer is beneficial for the catalytic process. As shown in Fig. 6k, EAG exhibited a higher specific activity than that of the free enzyme (by 5.1–6.2 times) for 5–20 mM AP, and its enantioselectivity remained above 99% without any decrease. These results demonstrate that the rearrangement of the substrate-binding pockets enhanced the hydrogen-transfer process while maintaining the enantioselective conformation.

### Proposed mechanism of EAG formation

Since the non-specific structure at the enzyme-assembled interface could not be resolved due to the technical bottleneck, a combination of experimental and MD simulations was used to reveal the possible mechanism of gel formation. The process of pre-gelation of Hmtz with enzyme molecules to form a complex, and the further gelation of the complex with Mg^2+^ to form EAG were analyzed successively. Thus, it was possible to extrapolate the mechanism of gel formation and the roles played by Hmtz and Mg^2+^ in this process.

**Fig. 7:**
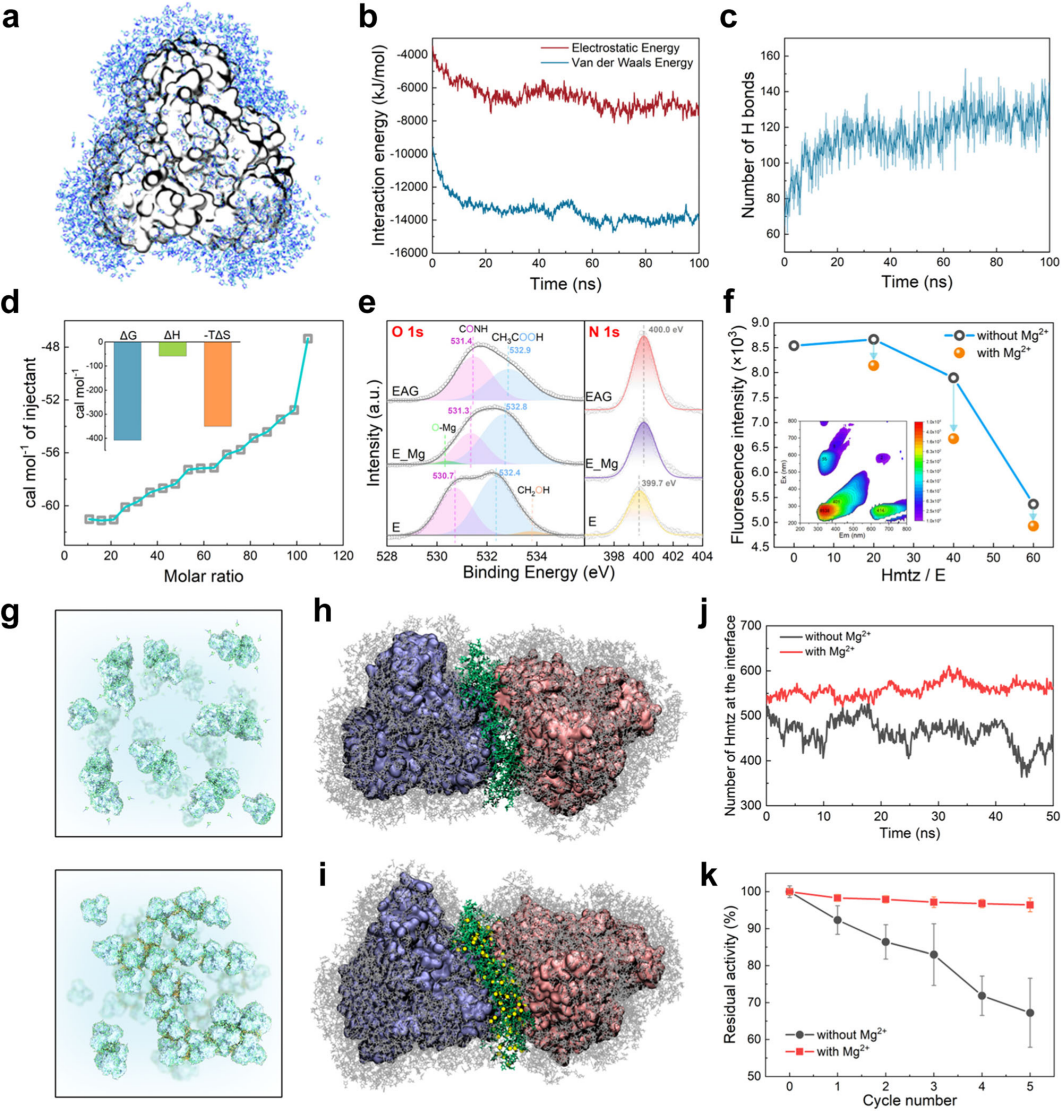
Mechanism of the EAG formation induced by Hmtz and Mg^2+^ ions. **a** Snapshot of the Hmtz interactions with a TbSADH tetramer during MD simulations. **b** Interaction energy between Hmtz and TbSADH plotted as a function of the simulation time. **c** Number of H bonds between Hmtz and TbSADH plotted as a function of the simulation time. **d** Isothermal titration thermograms obtained during the Hmtz titration into the TbSADH solution (0.1 mM). Inset shows the calculated thermodynamic data. **e** High-resolution O 1s and N 1s XPS profiles of E, E_Mg, and EAG. **f** Fluorescence intensities of E_Hmtz (without Mg^2+^ ions) and EAG (with Mg^2+^ ions) at (Ex, Em) = (280 nm, 335 nm) plotted as functions of the Hmtz:E ratio measured via EEM spectroscopy. Inset shows the EEM spectrum of E (Hmtz:E = 0). **g** Schematic of the (upper) weak gels without Mg^2+^ ions and (down) strong gels with Mg^2+^ ions. (**h**) Snapshot of two TbSADH tetramers bonded to Hmtz obtained during MD simulations. The Hmtz species located at the interface between the two tetramers are colored in green. **i** MD-simulated structure of two TbSADH tetramers with Hmtz and Mg^2+^ ions. Yellow beads represent Mg^2+^ ions. **j** Number of Hmtz species located at the interface between the two tetramers during MD simulations. **k** Stability of the gels synthesized with and without Mg^2+^ ions during five use cycles. Error bars represent the standard deviations from three independent experiments. Source data are provided as a Source data file.

The first step in EAG formation is the pre-gelation process. The results of MD simulations indicate that Hmtz will interact with the TbSADH surface to form an enzyme–Hmtz complex (E_Hmtz) (Fig. 7a). In the pre-gelation experiments, a solid-phase product was indeed generated, and an increased N 1s signal intensity was observed in its XPS survey spectrum, confirming the contribution of Hmtz in this complex product (Supplementary Fig. 28). To experimentally study the interactions, the binding process of Hmtz on the enzyme surface was measured using isothermal titration microcalorimetry (ITC). The dissociation constant *K*_d_ of the TbSADH–Hmtz complex measured by ITC was 7.7 × 10^−4^ M, which corresponded to non-specific weak intermolecular interactions (Supplementary Fig. 29). Because TbSADH tetramers form tenon-mortise joints in the 3D space, subunits of enzyme molecules can be weakly bridged with the induction of Hmtz, which leads to the weak aggregation of enzyme molecules (Fig. 2a2). The measured zeta potential of the protein surface decreased with increasing Hmtz:E ratio, which also confirmed the Hmtz-driven protein aggregation process (Supplementary Fig. 5). Subsequently, MD simulations were performed to determine interaction types.^35^ The obtained results revealed that Hmtz was bonded to the protein surface through H bonds and hydrophobic interactions (Fig. 7b, c), and the potential locations are exemplified in Supplementary Fig. 43. Moreover, Hmtz served as a binder facilitating the formation of cross-linked tetramers (Fig. 7h). Furthermore, the values of ΔH < 0 and ΔS > 0 obtained for the interaction process using ITC (Fig. 7d) experimentally confirmed that the interactions between Hmtz and proteins mainly included hydrogen bonding, van der Waals forces, and hydrophobic interactions. Significantly, the gel amounts generated by various triazoles increased in the order 3-Me-3N (Hmtz) > 3-NO_2_-3N > 3 NH_2_-3N ≈ 1-Me-3N > 3N (Fig. 3a). The structure of Hmtz is unique in having both an N–H group on the thiazole ring and a substituted –CH3 hydrophobic group, which provide a superimposed driving force for the interaction with the protein through H-bonding and hydrophobic interactions. The N–H groups can form strong hydrogen bonds with the carboxyl oxygens on the protein surface. MD simulations show obvious hydrogen bonding of the N–H of Hmtz towards the carbonyl oxygen of the glutamate and aspartate residues of the protein (Supplementary Fig. 43b, c). As a result, the shielding effect of Hmtz binding on the TbSADH surface also significantly reduced the O 1s XPS peak intensity of the COOH groups^36^ in the EAG structure compared with that of the pure enzyme. As shown in Fig. 7e, the O 1s signals in the high-resolution XPS spectra were mainly from amide oxygen (CONH, pink peak) and carboxyl oxygen (COOH, blue peak). For E and E_Mg, the photoelectron signal peaks of COOH were significantly higher than those of CONH. While for EAG containing Hmtz, the trend was reversed, and the COOH peak was significantly reduced and even lower than that of CONH, indicating the binding of Hmtz to carboxyl oxygen. In addition, MD simulations also demonstrate the interaction of –CH3 groups of Hmtz with hydrophobic amino acid residues on the protein surface (Supplementary Fig. 43e, f). The increased hydrophobicity of the gel caused by the Hmtz bonding to the protein surface was also confirmed using water adsorption isotherms (Supplementary Fig. 30).

The second step in EAG formation is the gelation process of E_Hmtz and Mg^2+^ to form EAG. The weakly aggregated interface (rich in Hmtz) between enzymes facilitated the chelation of Mg^2+^ ions, which further locked Hmtz species onto the interface. The presence of Mg^2+^ ions in the gel network was verified via EDX and XPS (Fig. 2e and Supplementary Fig. 28). As shown in Fig. 7i, j, the addition of Mg^2+^ ions increased the Hmtz content at the interface and decreased its fluctuations, indicating that the chelation effect of Mg^2+^ ions can strengthen the interface and promote the formation of a stable gel (Fig. 7g). Then how does Mg^2+^ interact with the interface networks? MD simulations show that the coordination of Mg^2+^ in the bridging interface depends mainly on the N atoms of Hmtz, and the N and O atoms of proteins, and some local coordination structures are exemplified in Supplementary Fig. 44. XPS analysis data confirmed that the N and O atoms on the protein surface were mainly involved in the coordination with Mg^2+^ ions at the bridging interface (Supplementary Fig. 31), which increased the binding energies of both the O 1s and N 1s electrons. As shown in Fig. 7e, compared with E, the O 1s electron binding energies of amide bond and carboxyl group in E_Mg increased from 530.7 and 532.4 eV to 531.3 and 532.8 eV, respectively; and its N 1s electron binding energy increased from 399.7 to 400.0 eV. In addition, tryptophan in TbSADH was used as an endogenous probe for fluorescence quenching experiments to study the interactions of the protein surface with Hmtz and Mg^2+^ ions. Excitation–emission–matrix (EEM) spectroscopy was utilized to determine the fluorescence intensity of the gels synthesized at different Hmtz:E ratios. As shown in Fig. 7f and Supplementary Fig. 32, when the Hmtz:E ratio increases from 0 to 20, the fluorescence intensity enhanced due to the increased exposure of fluorescent residues caused by conformational changes (Supplementary Fig. 33); however, at Hmtz:E ratios higher than 20, the fluorescence intensity decreased with an increase in the Hmtz:E ratio. The fluorescence quenching confirmed the interactions between Hmtz and tryptophan^37^. After the addition of Mg^2+^ ions, the fluorescence was further quenched (Fig. 7f), indicating that tryptophan partially contributed to the chelation of Mg^2+^ ions.

According to the obtained results, both Hmtz and Mg^2+^ are indispensable for the formation of highly active and stable EAG and produce a synergistic induction effect on the formation of bridging interfaces. As shown in Fig. 7k, the involvement of Mg^2+^ ions in interfacial chelation improved the stability of the gel, which effectively reduced enzyme shedding during the repeated use of EAG. Compared with the enzyme without Mg^2+^ ions, the residual activity of EAG increased from 67.2% to 96.4% after five uses. Based on these insights, this strategy was used for the immobilization of two other alcohol dehydrogenases with different structures, thus broadening the application of this method. ADHs from *Levilactobacillus brevis* (LbADH) and *Caldanaerobacter subterraneus* (CsADH) were successfully made into corresponding EAG, respectively, and used for the asymmetric reduction of ketones presenting excellent reusability. LbADH EAG was used to reduce AP, retaining 88.3% of the initial activity after being reused 10 times and maintaining a chiral selectivity of more than 99% for *R*-alcohol (Supplementary Fig. 41). CsADH EAG was used to reduce 1-hydroxy-2-butanon, retaining 91.5% of the initial activity after being reused 10 times and maintaining a chiral selectivity of ~82% for *R*-alcohol (Supplementary Fig. 42). It is evident that this immobilized biomaterial made by this facile strategy has excellent reusability and offers opportunities for biocatalytic synthesis and process development.

In summary, this study demonstrates a carrier-free immobilization strategy for the formation of EAG through the weak force-coupled multisite bridging between TbSADH molecules by the synergistic induction of Hmtz and Mg^2+^ ions. The prepared EAG exhibited a porous structure with pore sizes of 3–900 nm, which was used to catalyze the asymmetric reduction of AP, BAT, and EBP with specific activities exceeding those of the free enzyme by 4.1, 5.1, and 3.6 times, respectively. The results of intrinsic kinetic tests revealed that the *k*_cat_ of EAG determined for AP reduction was 6.3 times higher than that of the free enzyme, while its activity was retained at 90.3% after 12 cycles, indicating significantly the enhanced catalytic performance of EAG. Furthermore, a high-resolution (2.1 Å) structure of the EAG enzyme was obtained using cryo-EM. Based on the obtained cryo-EM structure and MD simulation data, a structure–property relationship was established to explain the enhanced activity and stability of EAG. Meanwhile, the coordination effect of Mg^2+^ ions improved the enzyme robustness, which resulted in higher stability than that of the free enzyme against various unfavorable environmental factors. In addition, the mechanism of EAG formation was elucidated, and the synergistic induction effect of Hmtz and Mg^2+^ ions during the gelation process was confirmed. These insights guided the use of this strategy for two other ADHs with different structures, and the produced immobilized EAG for the asymmetric reduction of ketones exhibited excellent reusability. Future applications can be further explored on more types of ADH, and even for the preparation of heterogeneous catalysts for multi-enzyme co-immobilization, enabling the construction of efficient tandem reactions or cyclic conversion systems. Moreover, this immobilization strategy is suitable for designing microfluidic reactors for continuous synthesis. It is expected that the present strategy may provide a pathway for the industrial application of biocatalytic transformation and development of biocatalytic materials and process technologies.

## Methods

### Protein expression and purification

*E. coli* BL21 (DE3) cells containing the recombinant plasmid were cultivated in 5 mL LB medium for overnight at 37 °C, mixed with 50 μg/mL kanamycin for pET28a vector or 100 μg/mL ampicillin for pET22b vector, respectively. The culture was then transferred into 1 L of TB medium containing kanamycin (50 μg/mL) or ampicillin (100 μg/mL) and grown at 37 °C until OD600 = 0.6 was reached. Then the culture was induced with 0.2 mM isopropyl β-D-1-thiogalactopyranoside (IPTG) and allowed to grow for additional 12 h at 20 °C. The cells were harvested by centrifuging at 6000 × *g* for 15 min at 4 °C, the pellet was then washed with phosphate buffer (50 mM, pH 7.4), and resuspended using 50 mM phosphate buffer under pH 7.4. The cells were lysed by sonication and the supernatant was collected by centrifugation at 10,000 × *g* for 60 min at 4 °C. Protein purification was performed at 4 °C using an AKTA Purifier system and analyzed by SDS-PAGE, protein concentration was measured using absorbance at 280 nm.

### Cryo-EM sample vitrification and data acquisition

A phosphate buffer solution enriched with EAG-derived alcohol dehydrogenase was obtained by placing EAG in, shaking, and centrifugation for multiple times. The enzyme solution concentrated to approximately 1 mg/mL was incubated for 10 min at 8 °C. Quantifoils (Cu R1.2/1.3, 300 mesh carbon grids) were glow-discharged for 30 s at a medium power setting, and aliquots of 4 μL sample were spread on them. By using a Vitrobot Mark IV (FEI), the grids were blotted for 2.5 s at 8 °C and 100% humidity, and then flash-frozen in liquid ethane cooled by liquid nitrogen after waiting for 10 s. Grids were transferred to a 300 kV Titan Krios (FEI) electron microscope equipped with a Gatan K3 Summit detector and a GIF Quantum energy filter. By using AutoEMation^38^, zero-loss movie stacked micrographs (32 frames each stack) were automatically acquired with a 20-eV slit width on the energy filter and a defocus range of −0.7 to −1.6 μm in super-resolution mode at a nominal magnification of ×105,000 with 0.8374 Å/pixel.

### Image processing

Each stack was exposed for 1.28 s with an exposure time of 0.03996 s per frame and recorded as a movie of 32 frames, resulting in the total dose rate of approximately 50 electrons/Å^2^ for each movie stack. The stacks were preprocessed by using TsinghuaTitan software package (motion-corrected by MotionCor2^39^ and contrast transfer function (CTF) estimation with Gctf). The data were processed for structure reconstruction with CryoSparc software^40^. A total of 7064 movies were collected, and 2,410,644 particles were autopicked in Template-picker method. After 2D classification, 742,106 particles were selected and subjected to initial model reconstruction. The resulting total 2,032,232 particles from 2D classification were subjected to several rounds of 3D classification. At last, a total of 1,254,132 particles were selected and subjected to a 3D autorefinement with an overall mask, resulting in a 2.12 Å resolution map with D2 Symmetry and 2.36 Å resolution map with C1 Symmetry after postprocessing.

### Model building and refinement

Wild-type TbSADH (PDB ID: 1YKF) was used as an initial model and fitted into the electrostatic potential (ESP) map using UCSF Chimera^41^. The model was rebuilt manually with Coot^42^. Metal ions in TbSADH were placed by visual inspection and then merged into the map using Coot. Further automated refinement was performed with PHENIX^43^.

### Preparation of E_Hmtz, E_Mg, and EAG

Hmtz (60 mg/mL) was first added to a phosphate buffer solution (50 mM, pH = 7.4) containing 1 mg/mL TbSADH, and the obtained mixture was stirred at 220 rpm and 25 °C for pre-gelation. E_Hmtz was collected via centrifugation and washed twice with pure water after pre-gelation for 2 h. For EAG preparation, a Mg(NO_3_)_2_·6H_2_O aqueous solution (10 mM) was added to the mixed solution of TbSADH and Hmtz at a Δ*t* of 8–10 min and stirred continuously for 2 h for gelation. EAG was obtained via centrifugation and washed twice with pure water. For E_Mg preparation, 1 mg/mL TbSADH was mixed with 200 mM Mg(NO_3_)_2_·6H_2_O to produce a solid precipitate, and E_Mg was collected via centrifugation and washed twice with pure water after precipitation for 2 h. All the lyophilized samples were obtained by rapid freezing with liquid nitrogen and then transferred to a freeze dryer for 8 h. The protein content in the synthesized complexes was determined using UV absorption spectroscopy after dissolution in a 10% (w/v) sodium dodecyl sulfate solution (Supplementary Fig. 35). To conduct screening experiments involving different triazoles and metal ions, equimolar amounts of other triazoles and metal ions were used to replace Hmtz and Mg^2+^ ions, respectively, and the corresponding EAGs were synthesized according to the above-mentioned procedure.

### Determination of catalytic activity

Unless specified otherwise, the reaction conditions for activity determination were 5 mM AP, 10% IPA, 1 mM NADP^+^, and 1 mg/mL TbSADH in 500 μL of phosphate-buffered saline (50 mM, pH = 7.4). The enzyme concentration of 1 mg/mL used for all reactions in this work, as determined according to literatures^5,44^, means that the free enzyme or immobilized enzyme with a TbSADH content of 1 mg was used for 1 mL of reaction solution. The reaction was conducted at 30 °C for 10 min. The obtained samples were extracted with ethyl acetate for chiral HPLC detection using an Agilent 1260 instrument (Agilent Technologies Singapore (International) Pte. Ltd., Singapore) equipped with a Chiralcel OD-H column (4.6 mm × 250 mm, 5 μm, Diacel). HPLC analysis was performed at a temperature of 30 °C and detection wavelength of 220 nm, and an *n*-hexane:IPA mixture (92:8, v/v) was used as the eluent at a flow rate of 1.0 mL/min. The catalytic conversion was determined by the measured substrate concentration versus the standard calibration (Supplementary Fig. 36). The chirality and e.e. of the alcohol products were determined by comparing the chiral HPLC results with the racemic standards (Supplementary Figs. 37–39). The catalytic yield was determined by the measured alcohol concentration versus the standard calibration (Supplementary Fig. 40). The retention times of each substance are listed in Supplementary Table 4.

### Cryo-TEM

After pre-gelation or gelation for a specified time, 4 μl of the solution was sampled and rapidly applied to a glow-discharged Quantifoil (Cu R1.2/1.3, 300 mesh carbon grid). On a Vitrobot Mark IV (FEI), the grid was blotted with filter paper for 2.5 s (8 °C, 100% humidity) to remove excess sample. After waiting for 15 s, the grid was immersed in liquid ethane (−180 °C) cooled by liquid nitrogen. Cryo-TEM images were acquired on the FEI Falcon II Direct Electron Detector on a Tecnai Arctica (FEI) microscope equipped with a Gatan Orius SC200B (830) CCD.

### MD simulation

PROPKA 3^45^ was employed to assign the protonation states of titratable residues. And the protonation states and side chain orientations were also checked by visual inspection. All crystal waters were removed. All MD simulations were conducted using the GROMACS 2019.3, along with the Amber ff14SB^46^ force-field parameters and the reoptimized parameters for the ω torsional angle for protein. The parameters for Hmtz and AP were generated with the antechamber module of Amber18 using the general Amber force field (GAFF)^47^, with partial charges set to fit the electrostatic potential generated with HF/6-31G(d) by RESP. For the Zn^2+^ at the active site and Mg^2+^ in 7XY9, their parameters were calculated using MCPB.py. The system was placed in a periodic cubic box of water molecules represented by the three-point charge TIP3P model. The total system was energy minimized by a succession of steepest descent and conjugate gradient methods. Thereafter, it was equilibrated for 100 ns at constant temperature (298.15 K) and pressure (1 bar) (NPT). We used V-rescale thermostat and Parrinello–Rahman barostat to keep the temperature and pressure constant, respectively. The cutoff radius for neighbor searching and nonbonded interactions was taken to be 12 Å, and all bonds were constrained using the LINCS algorithm. All computed structures in MD simulations were illustrated using VMD.

### Reporting summary

Further information on research design is available in the Nature Portfolio Reporting Summary linked to this article.

## Supplementary information

Supplementary Information

Reporting Summary

## Data Availability

The cryo-EM atomic model has been deposited in the Protein Data Bank (PDB ID code 7XY9). The data that support the findings of this study are available within the manuscript, Supplementary Information, Source data file, and from the corresponding authors upon request. Source data are provided with this paper.

## Source data

Source Data
