# Supplementary material for: Active and stable alcohol dehydrogenase-assembled hydrogels via synergistic bridging of triazoles and metal ions
Source: Nat Commun. 2023 Apr 13;14:2117. doi: 10.1038/s41467-023-37921-y (PMC10102205; doi:10.1038/s41467-023-37921-y)
Supplement: Supplementary file 1 — Supplementary Information [file 41467_2023_37921_MOESM1_ESM.pdf]

Supplementary Information

for

Active and stable alcohol dehydrogenase-assembled hydrogels via synergistic bridging of triazoles and metal ions

Qiang Chen[a], Ge Qu[b], Xu Li[b], Mingjian Feng[a], Fan Yang[c], Yanjie Li[c], Jincheng Li[b], Feifei Tong[b], Shiyi Song[b], Yujun Wang[a,]*, Zhoutong Sun[b,]*, and Guangsheng Luo[a]

[a] *The State Key Lab of Chemical Engineering, Department of Chemical Engineering, Tsinghua University, Beijing 100084, China*

[b] *Tianjin Institute of Industrial Biotechnology, Chinese Academy of Sciences, Tianjin 300308, China*

[c] *Technology Center for Protein Sciences, School of Life Sciences, Tsinghua University, Beijing 100084, China*

*Corresponding author Yujun Wang: Tel.: 86-10-62798447

E-mail address: wangyujun@mail.tsinghua.edu.cn

*Corresponding author Zhoutong Sun: Tel.: 022-84861981

E-mail address: sunzht@tib.cas.cn

Table of Contents

Supplementary Methods ....3~5

Supplementary Figures 1 to 44 ....6~49

Supplementary Tables 1 to 4 ....50~53

Enzyme Sequences 54

Supplementary References 55

Supplementary Methods

Chemicals and Materials

Isopropanol (IPA) and nicotinamide adenine dinucleotide phosphate ($NADP^+$) were purchased from Energy Chemical Technology (Shanghai) Co., Ltd. Acetophenone (AP), benzyl acetone (BAT), ethyl 2-oxo-4-phenylbutyrate (EBP), 3-methyl-1,2,4-trizole (Hmtz or 3-Me-3N), 1-methyl-1,2,4-trizole (1-Me-3N), 3-nitro-1,2,4-trizole (3-NO_2-3N), 3-amino-1,2,4-trizole (3-NH_2-3N), 1,2,4-trizole (3N), imidazole (2N), and tetrazole (4N) were purchased from Shanghai Bide Pharmatech Co., Ltd. Magnesium nitrate hexahydrate, calcium nitrate tetrahydrate, and zinc nitrate hexahydrate were obtained from Sinopharm Chemical Reagent Beijing Co., Ltd. Cobaltous nitrate hexahydrate was supplied by Sigma-Aldrich. Copper (II) nitrate hydrate was supplied by Shanghai Macklin Biochemical Co., Ltd. Fluorescein isothiocyanate (FITC) and sodium dodecyl sulfate (SDS) were purchased from Shanghai Meryer Chemical Technology Co., Ltd. Uranyl acetate dihydrate was purchased from Shanghai Acmec Biochemical Co., Ltd.

Characterizations and Instruments

Scanning electron microscopy (SEM) images were acquired on a JEOL JSM-7900F (Japan) with an energy-dispersive X-ray (EDX) analyzer. Dynamic light scattering (DLS) was conducted on a Zetasizer Nano ZS90 (Malvern Instruments, UK). Transmission electron microscopy (TEM) was conducted on a JEOL 1011 electron microscope. EAG morphologies in solution were observed using a FV1000 confocal

laser scanning microscope (CLSM) and a confocal fluorescence microscope equipped with an IX-81 inverted base and a photomultiplier tube detector. Atomic force microscope (AFM) analysis was carried out on a Cypher ES. Fourier transform infrared spectra (FTIR) were measured by a PerkinElmer Frontier (USA). Raman spectra were recorded with a Horiba LabRAM HR Evolution (Japan). Water adsorption isotherms were recorded using a Quantachrome Autosorb IQ MP (USA). N_2 sorption isotherms were recorded using a Tristar II 3020 (Micromeritics, USA). Degassing process were under vacuum at 100 ºC for 12 h. The Barrett-Joyner-Halenda (BJH) pore size distributions were calculated from the desorption curves. Small-angle X-ray scattering (SAXS) were performed on a SAXSess MC2 (Anton Paar). Differential scanning calorimetry (DSC) were performed on a PerkinElmer DSC8000. Excitation-Emission-Matrix (EEM) spectra analysis were conducted on a Hitachi F7000. X-ray photoelectron spectroscopy (XPS) were measured on a Thermo Scientific K-Alpha. Isothermal Titration Calorimetry (ITC) was carried out on a MicroCal iTC200 (GE). UV-Vis absorption spectra were obtained with an UV-2450 spectrophotometer (Shimadzu, Japan). Organic elemental analysis was performed on an Elementar Unicube (Germany). Inductively coupled plasma optical emission spectrometer (ICP-OES) analysis was conducted on an Agilent 5110. ^{1}H and ^{13}C nuclear magnetic resonance (NMR) spectra were performed on a Bruker Avance III HD500.

Preparation of fluorescein isothiocyanate-labeled EAG

A 10-fold molar amount of fluorescein isothiocyanate (FITC) was added to a

phosphate buffer (50 mM, pH = 7.4) containing TbSADH, and the resulting solution was stirred in the dark at 4 °C for 5 h. Half-saturated ammonium sulfate was used to precipitate the labeled enzyme, and the supernatant containing free FITC molecules was poured out after centrifugation. Residual ammonium sulfate was removed via dialysis. FITC-labeled EAG was prepared from the labeled enzyme according to the previously described method.

Supplementary Figures

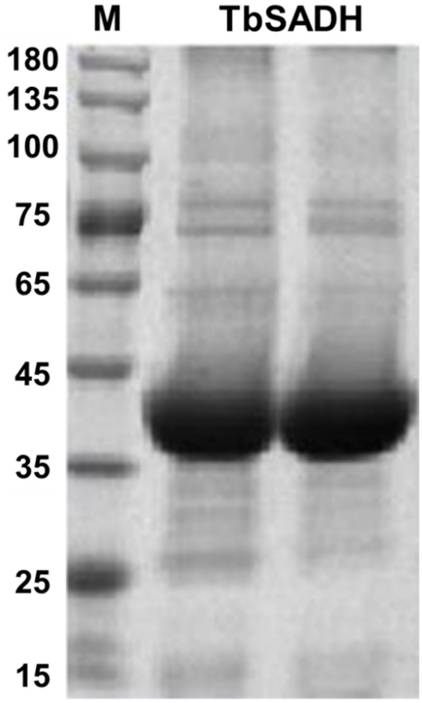

Supplementary Figure 1. SDS-PAGE of the TbSADH variant SZ2205[1] used for gelation to synthesize EAG. M: SDS-PAGE marker. The test was repeated twice. Representative images from two independent experiments are shown. The results confirmed that the enzyme used was of high purity. The molecular weight of the monomer is 37.6 kDa, and thus the calculated molecular weight of the tetramer is about 150 kDa.

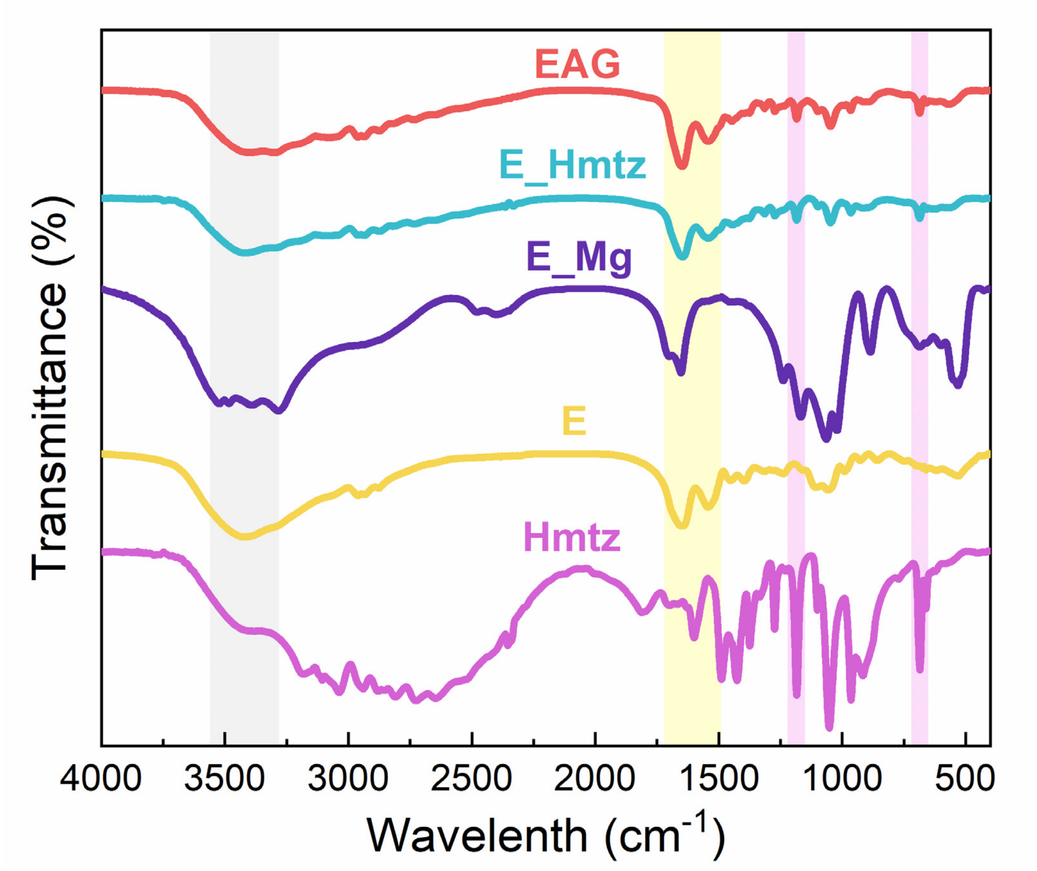

Supplementary Figure 2. FTIR spectra of Hmtz, E, E_Mg, E_Hmtz, and EAG at 4000-400 cm^{-1}. The yellow scheme marked the characteristic absorption peak (1650 and 1543 cm^{-1}) of the amide group of alcohol dehydrogenase. The pink scheme marked the characteristic absorption peaks of Hmtz. Peaks at 685 cm^{-1} are attributed to the C–N–N in-plane bending (β-CNN). Peaks at 1184 cm^{-1} are attributed to the N–H in-plane bending (β-NH).

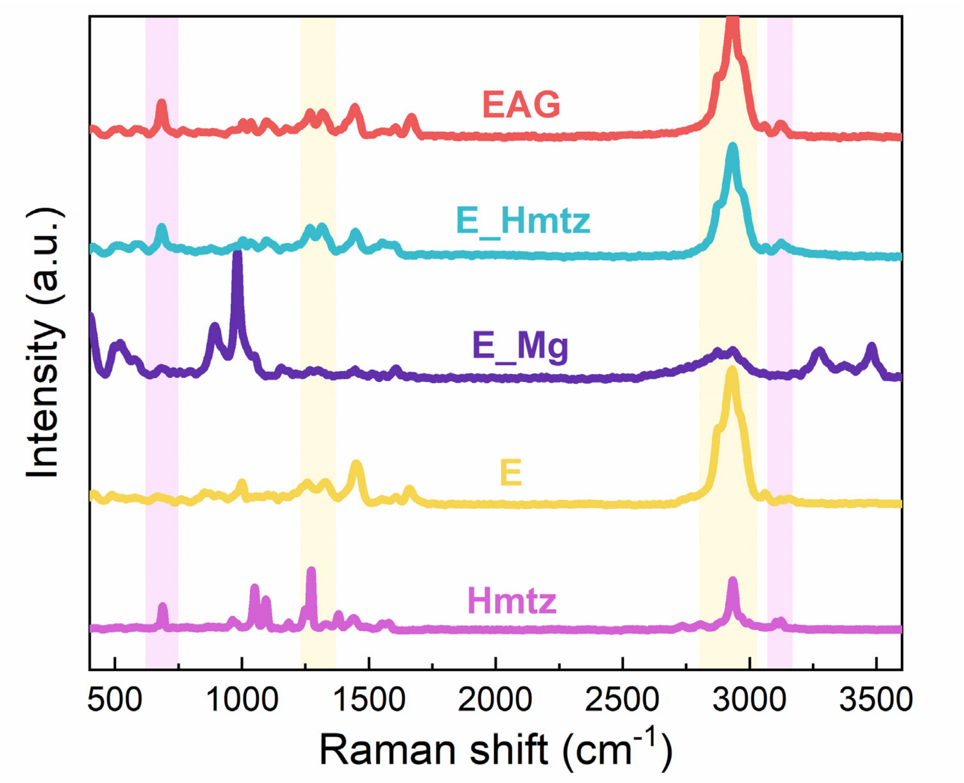

Supplementary Figure 3. Raman spectra of Hmtz, E, E_Mg, E_Hmtz, and EAG at 400-3600 cm^{-1}. The weak bands at 1258 and 1330 cm^{-1} are attributed to the stretching vibration of amide III of TbSADH. The moderate band at 685 cm^{-1} are attributed to the stretching vibration of *β*-CNN. The weak band at 3120 cm^{-1} is attributed to the N–H stretching (*v*-NH). The results confirmed the presence of Hmtz and TbSADH in E_Hmtz and EAG.

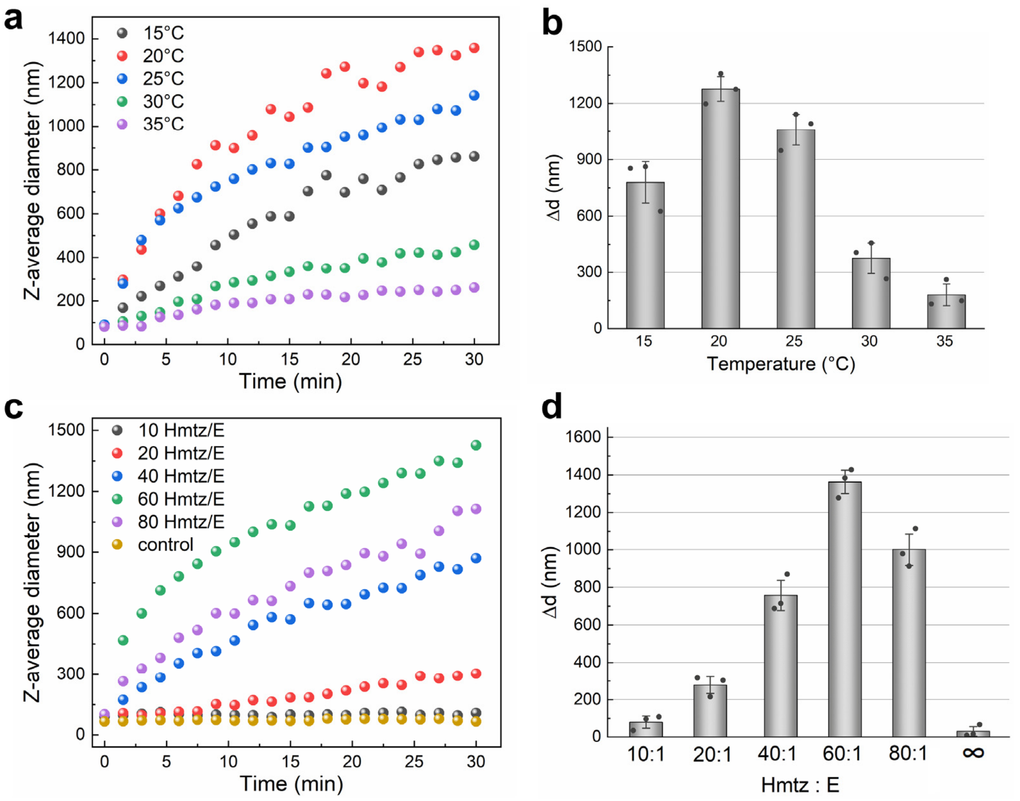

Supplementary Figure 4. Effects of temperature and the mass ratio of Hmtz to E on the pre-gelation process. (a) Growth of the hydrodynamic diameter (Z-average) of the E_Hmtz measured by DLS at different temperatures with time. Hmtz: E = 60:1 (mass ratio). (b) Comparison of diameter changes (Δ*d*) within 30 min at different temperatures. The results showed that the gelation rate was maximum at 20~25 °C. Considering that room temperature is more convenient and energy efficient, (pre-)gelation were carried out at 25 °C. (c) Growth of the hydrodynamic diameter at different ratios of Hmtz to E with time at 25 °C. (d) Comparison of Δ*d* within 30 min at different ratios. The results showed that the ratio of 60:1 was more conducive to the gelation process. For (b) and (d), $n = 3$. Data are expressed as the mean ± SEM. Error bars represent the standard deviations from three independent experiments.

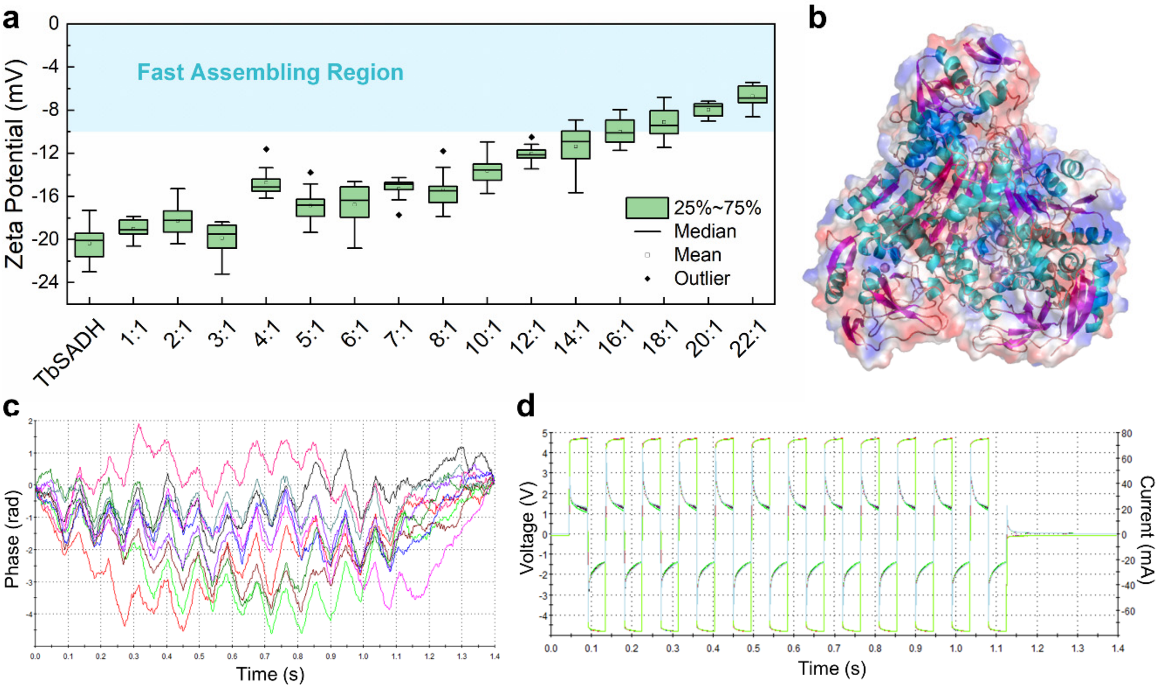

Supplementary Figure 5. Zeta potential analysis. (a) The changes of zeta potential with the increase of the ratio of Hmtz to E. The initial enzyme concentration was 5 mg/mL. n = 10. In order to exclude measurement errors and to obtain reliable trends, the results of ten tests for each condition were tallied into box plots for comparison. The box plots present the median (middle line) as well as the first and third quartiles (boxes), while the whiskers present 1.5 times the IQR above and below the boxes. The combination of Hmtz and enzyme led to the decrease of zeta potential, which further led to the aggregation between enzymes. When the ratio exceeded 20:1, the pre-gelation process became fast. (b) The relative surface potential of TbSADH, red and blue represent positive and negative potential respectively. (c) Phase plot as a function of time during a set of tests. (d) Voltage and current as functions of time during a set of tests.

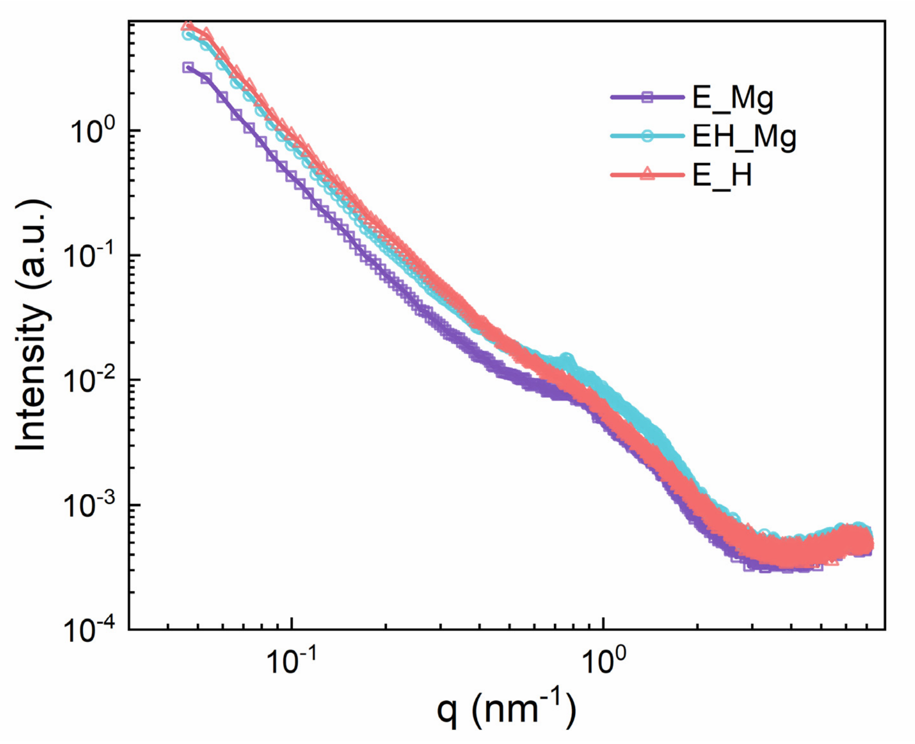

Supplementary Figure 6. Solid-phase SAXS profiles of E_Mg, E_Hmtz and EAG at scattering vector ranges of 0.04-7 nm^{-1}.

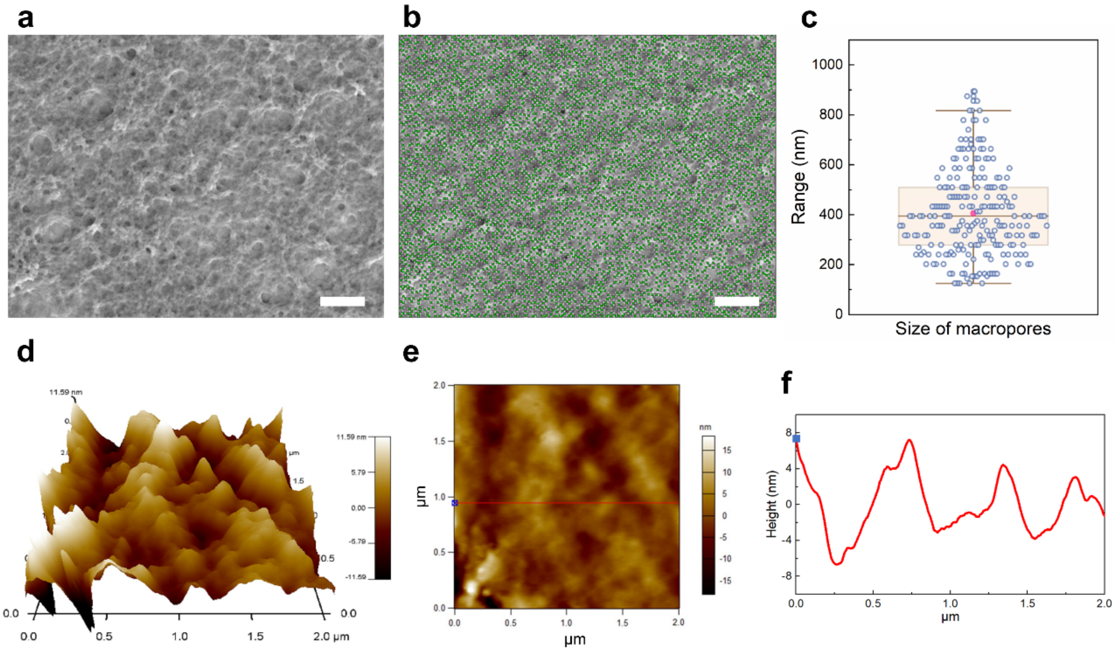

Supplementary Figure 7. Morphological characterization of EAG. (a) SEM images of the synthesized EAG with wide field of vision. Scale bar, 4 μm. (b) Porous topography highlighted after auto-marking high gray value areas. Scale bar, 4 μm. (c) Statistics of size distribution of macropores (>100 nm) of EAG. n = 225. The box plot presents the median (middle line) as well as the first and third quartiles (box), while the whiskers present 1.5 times the IQR above and below the box. This statistic is designed to provide information on the pore size distribution of the material from the electron microscope level. The mesopores was determined by N_2 adsorption-desorption experiments. (d) 3D and (e) 2D representations obtained by AFM. (f) Cross-sectional analysis, as marked by the red line in (e), suggests the hydrogel structure consists of an interconnected network.

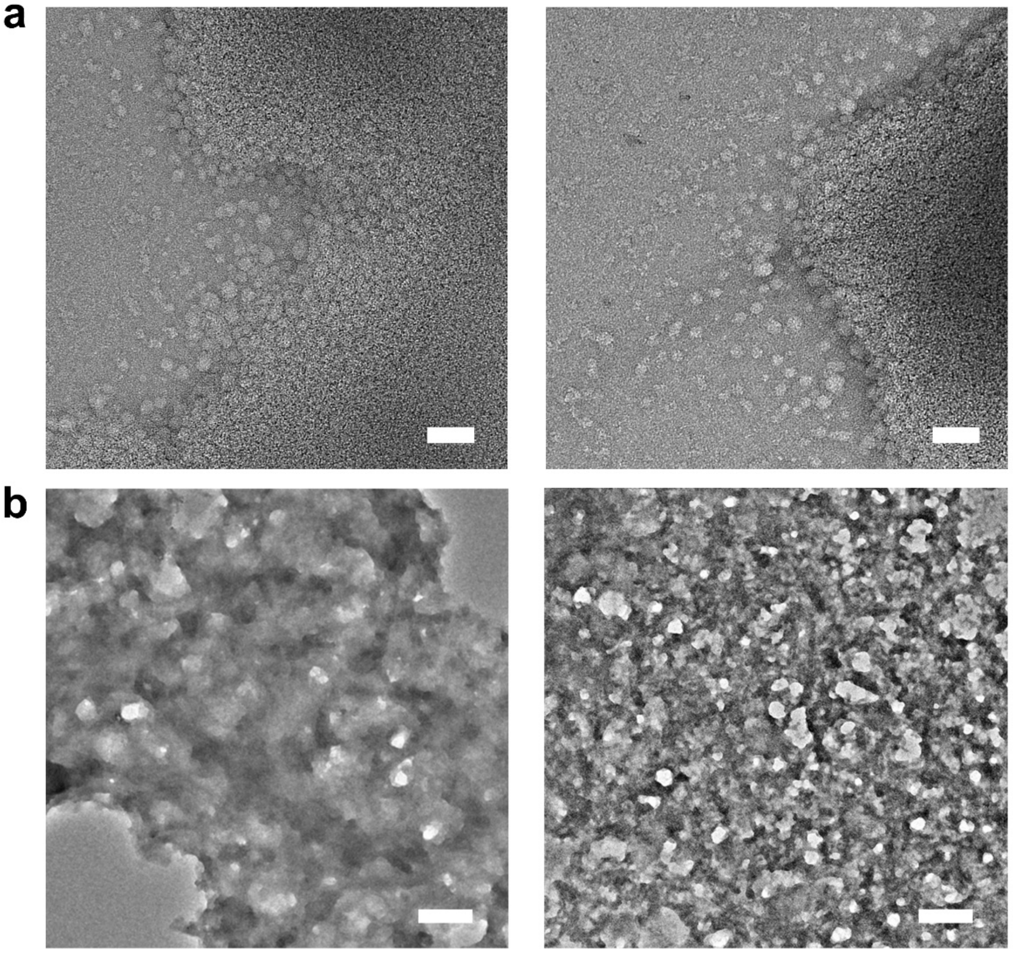

Supplementary Figure 8. (a) Negatively stained TEM images of EAG. Scale bar, 50 nm. White particles are enzymes. (b) TEM images of EAG. Scale bar, 500 nm. For (a) and (b), representative images from three independent experiments are shown.

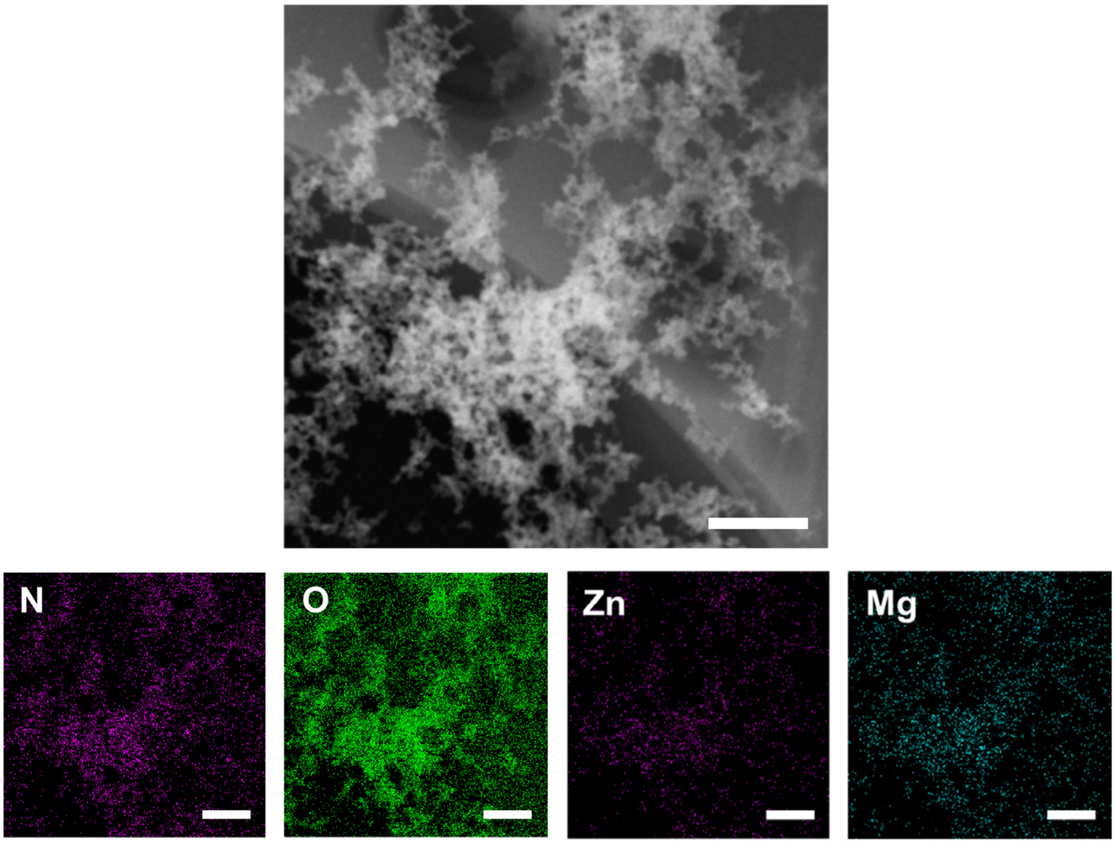

Supplementary Figure 9. Negatively stained TEM image of EAG at the beginning of gelation process and the corresponding energy-dispersive X-ray spectroscopy analysis. Representative images from three independent experiments are shown. When the gelation was carried out for 5 min, it was terminated immediately and negative-stained samples were prepared. Scale bar, 500 nm.

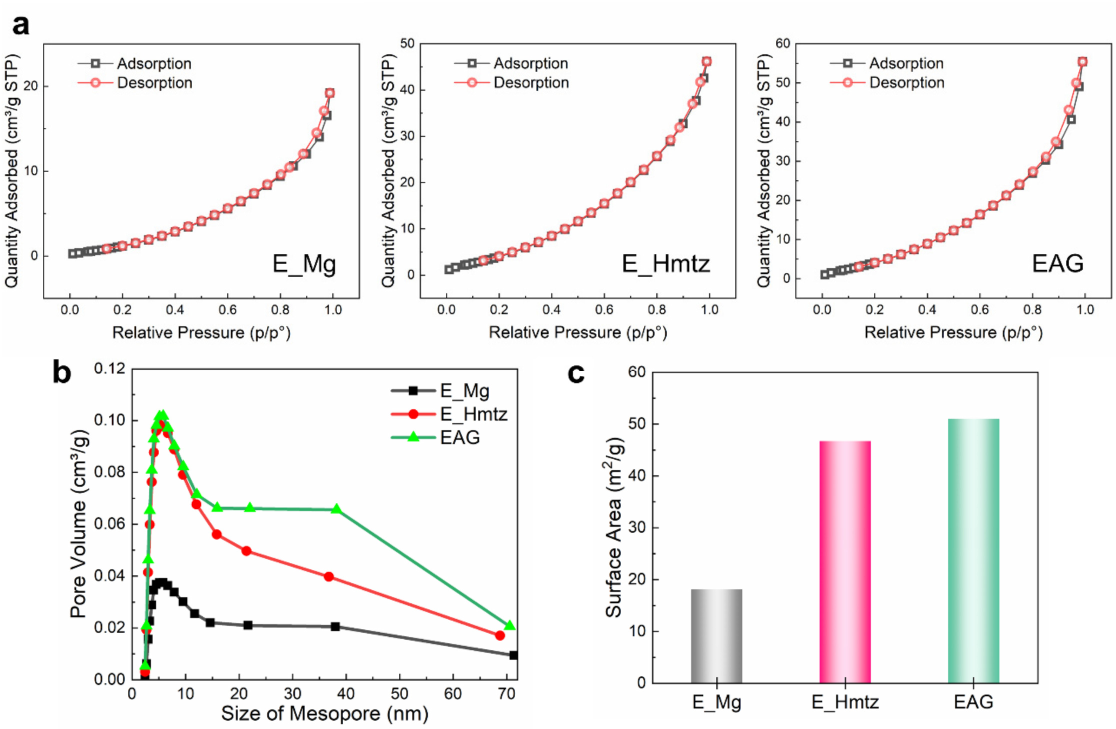

Supplementary Figure 10. N_2 physisorption characterizations of E_Mg, E_Hmtz and EAG. (a) adsorption-desorption isotherms. (b) Size distributions of mesopores (<100 nm). (c) Surface area of pores between 1.7 nm and 100 nm calculated from desorption curve.

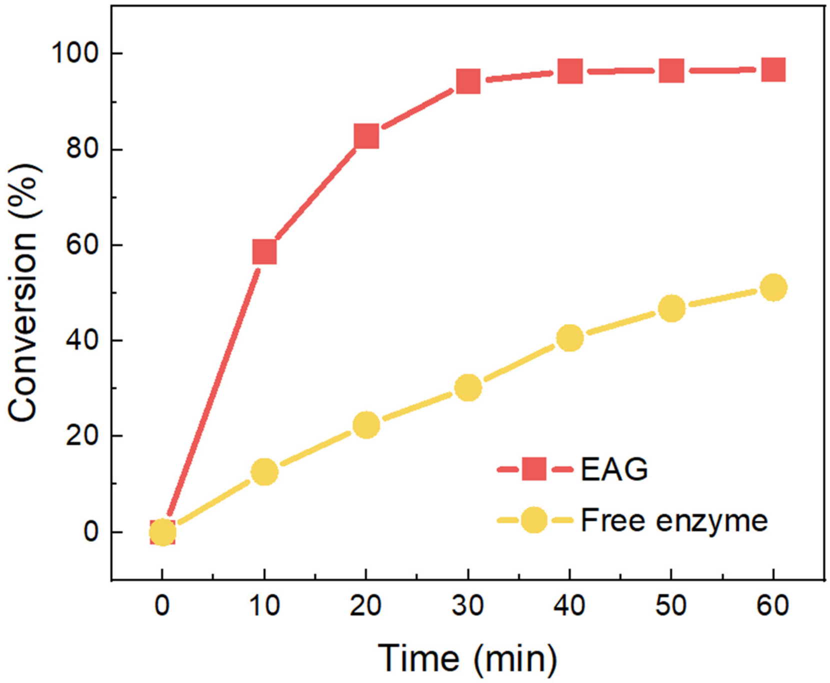

Supplementary Figure 11. Plots of AP conversions as functions of time in the reaction solution with enzyme content of 0.2 mg/mL provided by free enzyme or EAG.

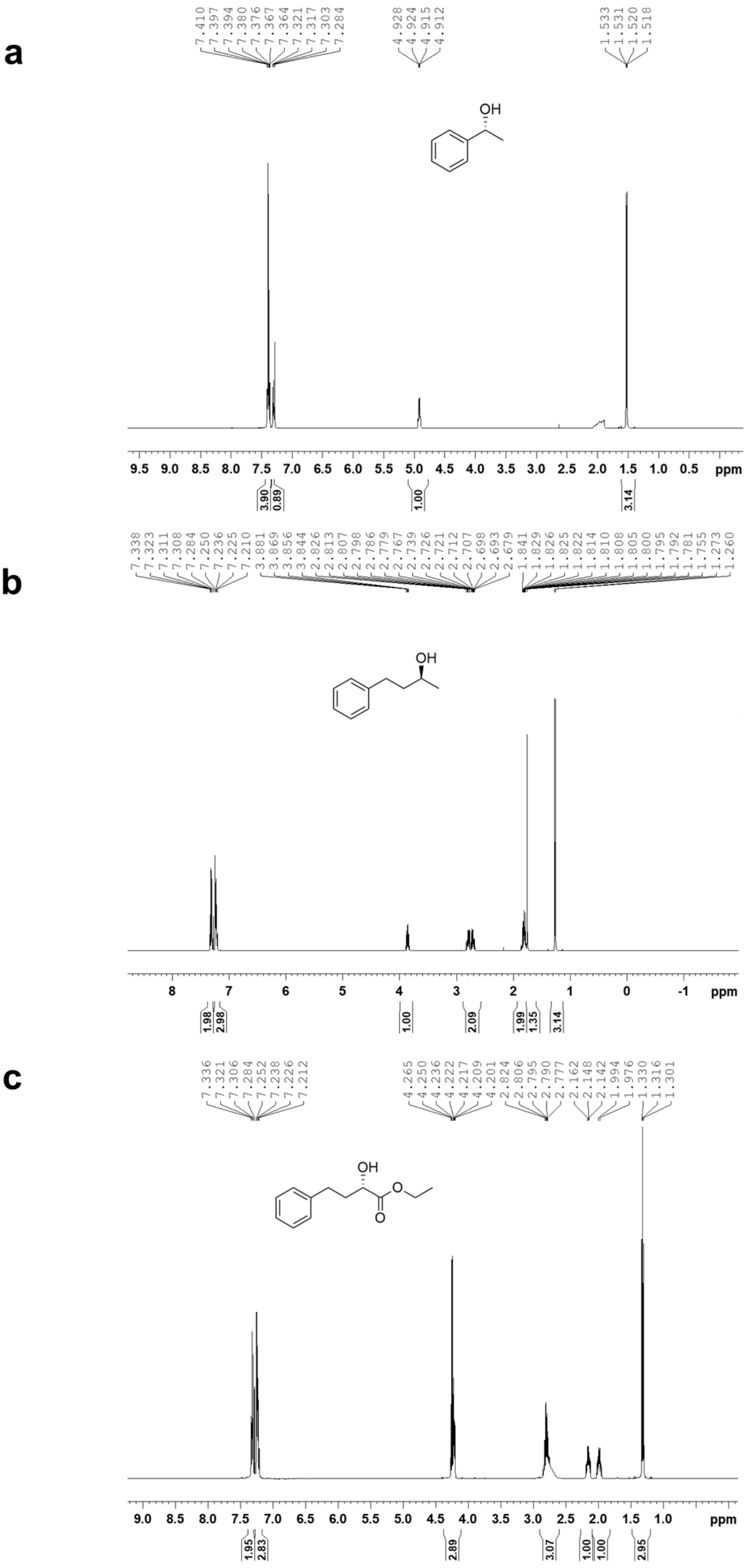

Supplementary Figure 12. ^1H NMR spectrum (500 MHz, $CDCl_3$) of compounds (a) AP, (b) BAT, and (c) EBP at 25 °C.

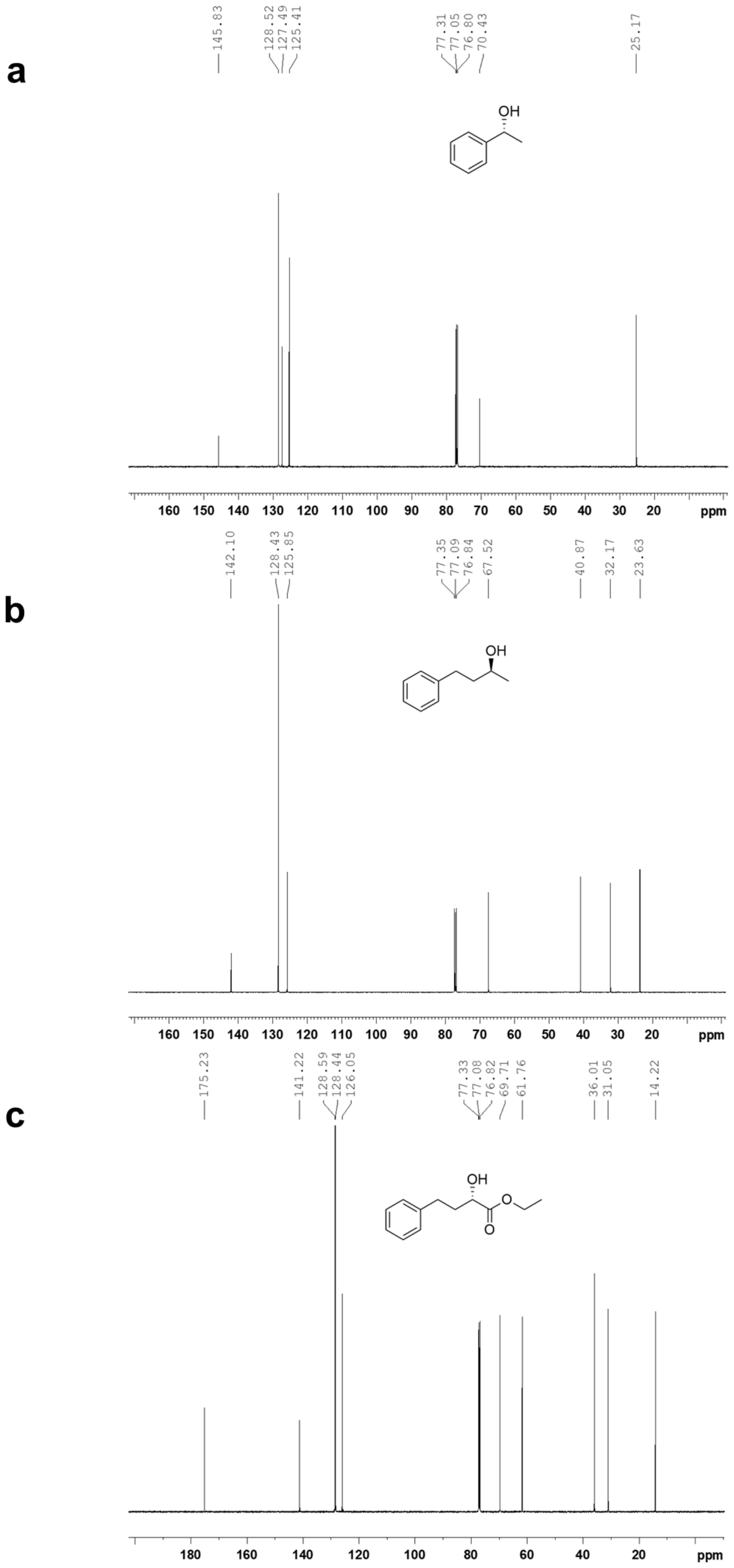

Supplementary Figure 13. ^{13}C NMR spectrum (500 MHz, $CDCl_3$) of compounds (a) AP, (b) BAT, and (c) EBP at 25 °C.

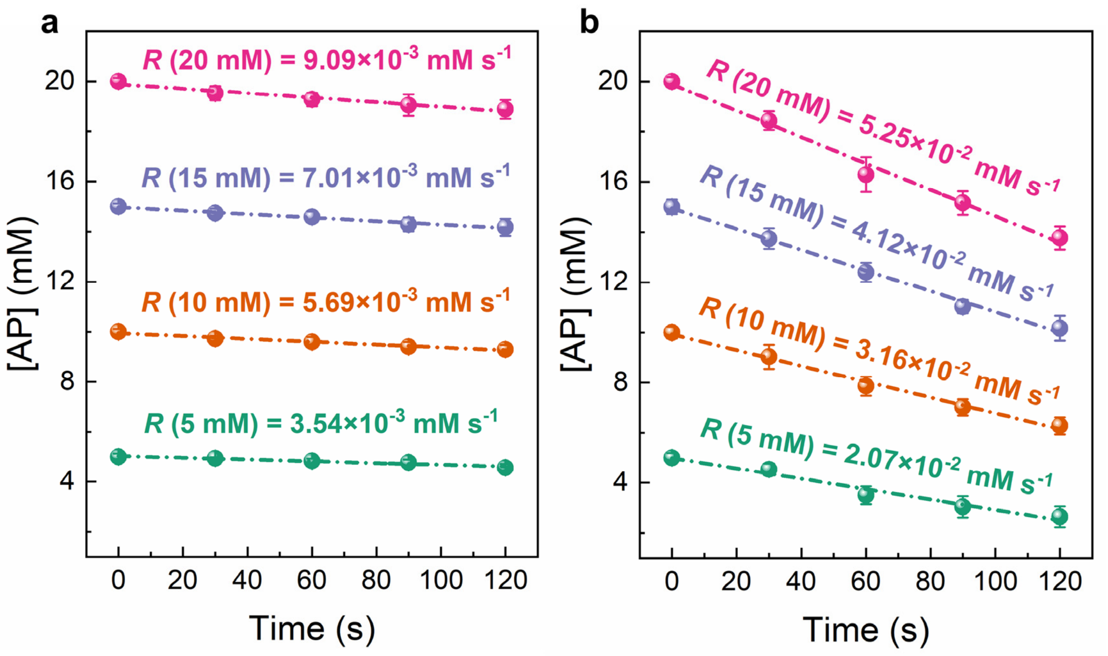

Supplementary Figure 14. Catalytic kinetics of (a) free enzyme and (b) EAG. The assay was performed in PBS (pH 7.4, 50 mM) with TbSADH concentration of 1 mg/mL and AP concentration in the range 5 to 20 mM. The error bars indicate the standard deviation of two independent measurements. *R* refers to the initial velocity of the reaction, which was obtained from the slope of the fitting line. Error bars represent the standard deviations from three independent experiments.

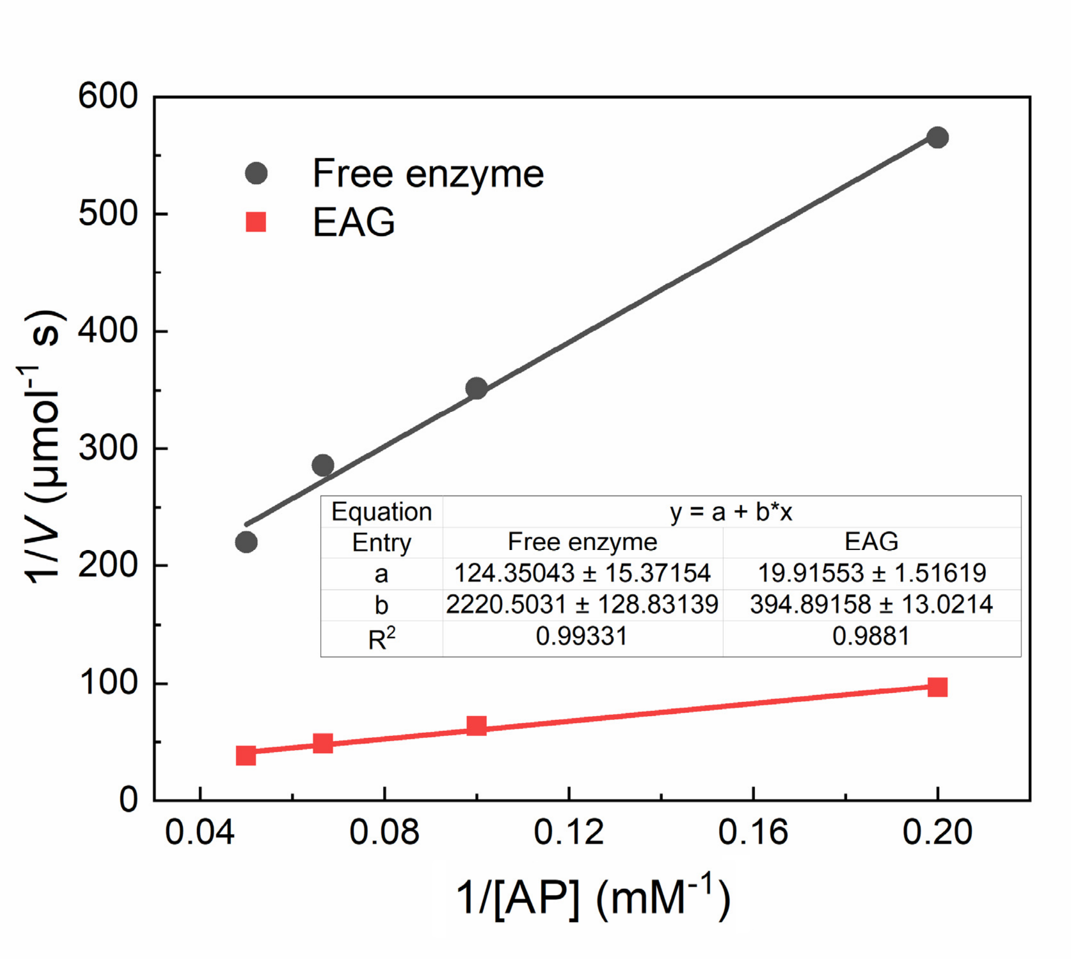

Supplementary Figure 15. Determination of V_{max} and K_m from plot of $1/V$ against 1/[AP] (0.05 to 0.2 mM^{-1}) for the asymmetric catalytic reduction of AP with free enzyme or EAG. The reactions were conducted in 50 mM PBS (pH 7.4) and 30 °C. V refers to the reaction velocity. The linear relationship between $1/V$ and 1/[AP] suggested that the reactions conform to the Michealis–Menten equation. Through the intercept and slope of the fitted line, the relevant parameters V_{max} and K_m can be calculated out (Supplementary Table 1).

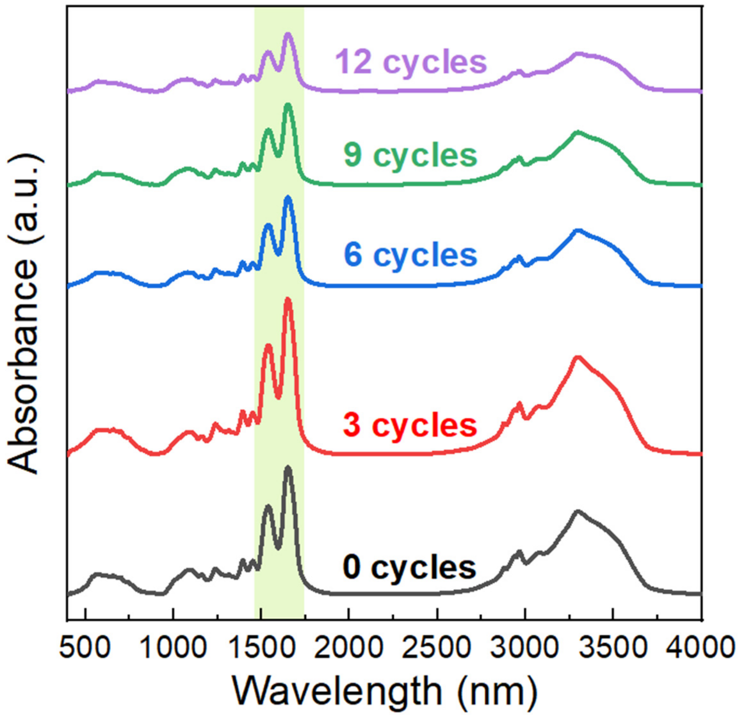

Supplementary Figure 16. FTIR spectra of EAG after repeated use for asymmetric reduction reactions. After each cycle, EAG was centrifuged and washed twice with PBS. After freeze drying, it was pressed to tablet to test the absorbance at different wavelengths.

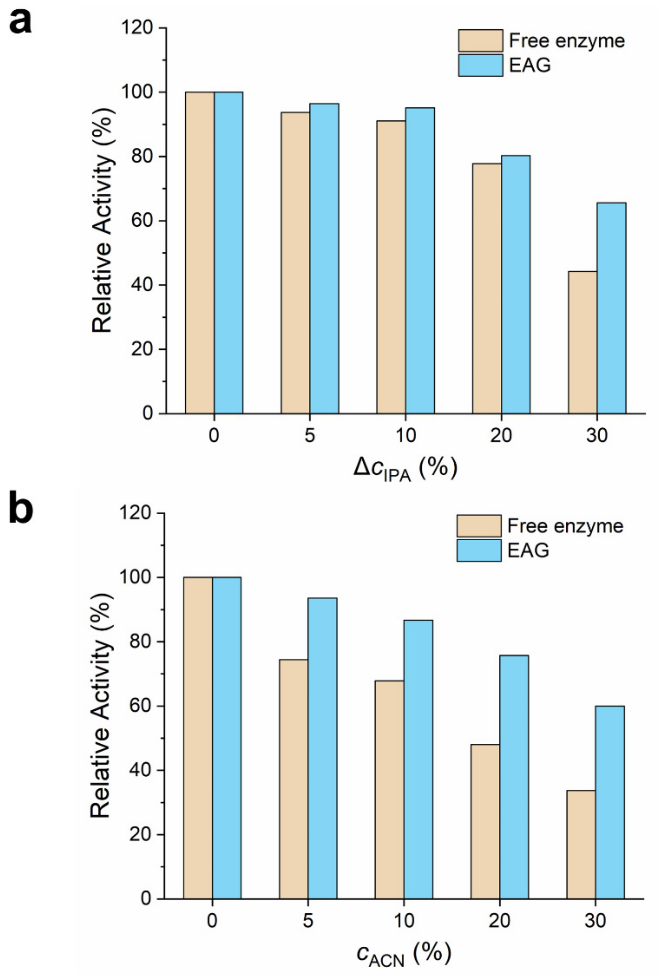

Supplementary Figure 17. Tolerance test of EAG to organic reagents. Relative activity of the assembled gel (EAG) and free enzyme (TbSADH) in the presence of different concentrations (a) isopropanol (IPA), and (b) acetonitrile (ACN). Data were normalized against the activity at 0%. EAG showed better tolerance, especially at high organic reagent concentrations.

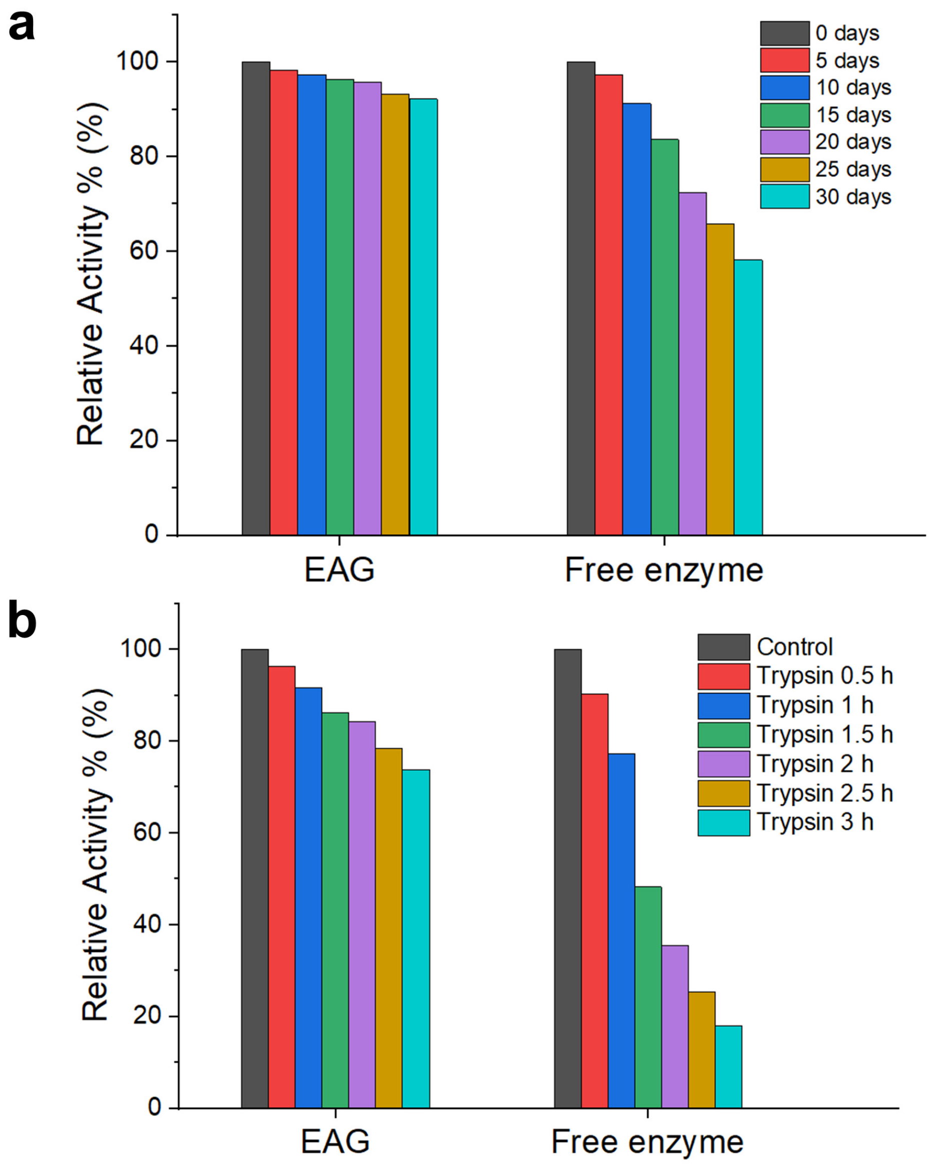

Supplementary Figure 18. Comparison of (a) storage and (b) digestion stability of EAG and free enzyme. Storage conditions: sealed and stored in an atmospheric pressure environment of 25 °C. Digestion conditions: 3 mg/mL trypsin treatment for a certain time. Data were normalized against the activity before storage or trypsin treatment. The results revealed that the EAG state provides better stabilization and protection for alcohol dehydrogenase.

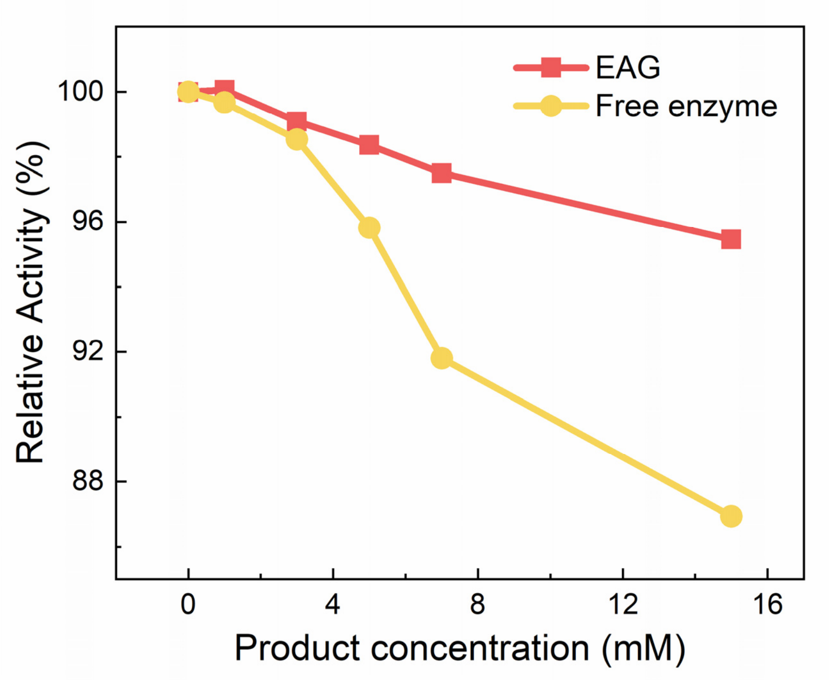

Supplementary Figure 19. Relative activities of EAG and free enzyme after additional addition of different concentrations (0-15 mM) of product. EAG exhibited significantly higher residual activity with increasing product concentration, indicating that the gel state is beneficial for alleviating product inhibition.

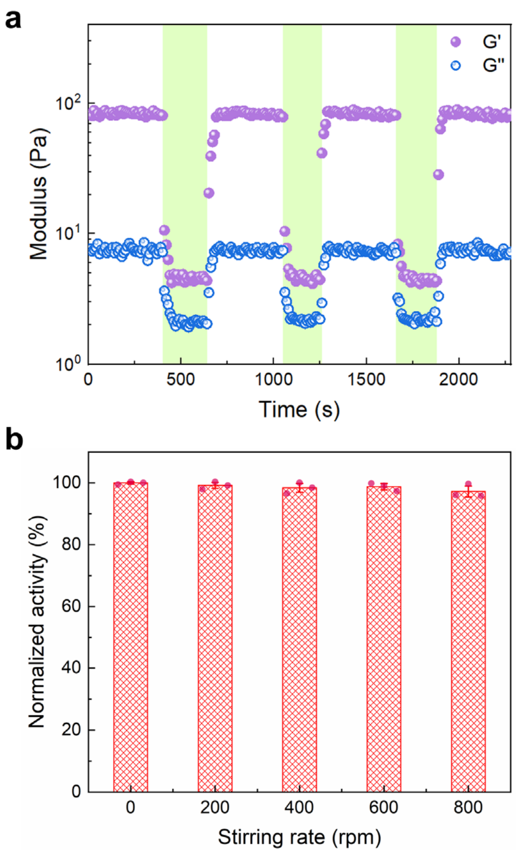

Supplementary Figure 20. Mechanical stability of EAG. (a) Three step modulus scans of the subjected EAG at 20% strain over 200 s (green columns), followed by a 400 s recovery period at 0.1% strain. G′ denotes the storage modulus, and G″ denotes the loss modulus. The frequency was constant at 1 Hz. (b) Normalized activity of EAG after stirring at different rates for 30 min. n = 3. These results show that the EAG was able to recover the modulus quickly within 40 s without serious hysteresis over multiple scan cycles. The mechanical strength was recovered rapidly and repeatedly even after three cyclic applied strains. Moreover, the activity of EAG showed negligible attenuation even after 800 rpm stirring. Therefore, we believe that EAG has good mechanical stability and is well suited for use in flow or batch reactors. Error bars represent the standard deviations from three independent experiments. Data are expressed as the mean ± SEM.

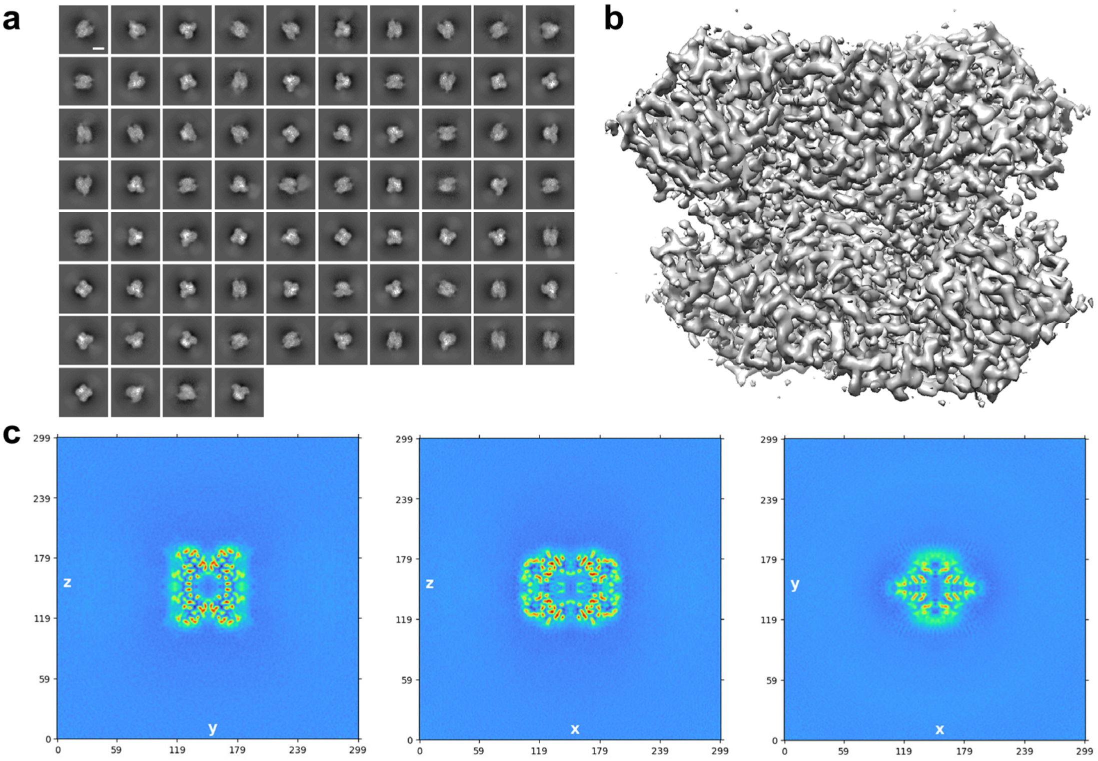

Supplementary Figure 21. (a) Supplementary representative 2D class averages of the enzyme from EAG. Scale bar, 5 nm. (b) The cryo-EM 3D reconstruction at 2.1 Å resolution from 1,254,312 particles. The contour level was set at 0.71. (c) Resolution maps of the central slices in three orthogonal directions. Local resolutions are between 1.8 and 2.6 Å.

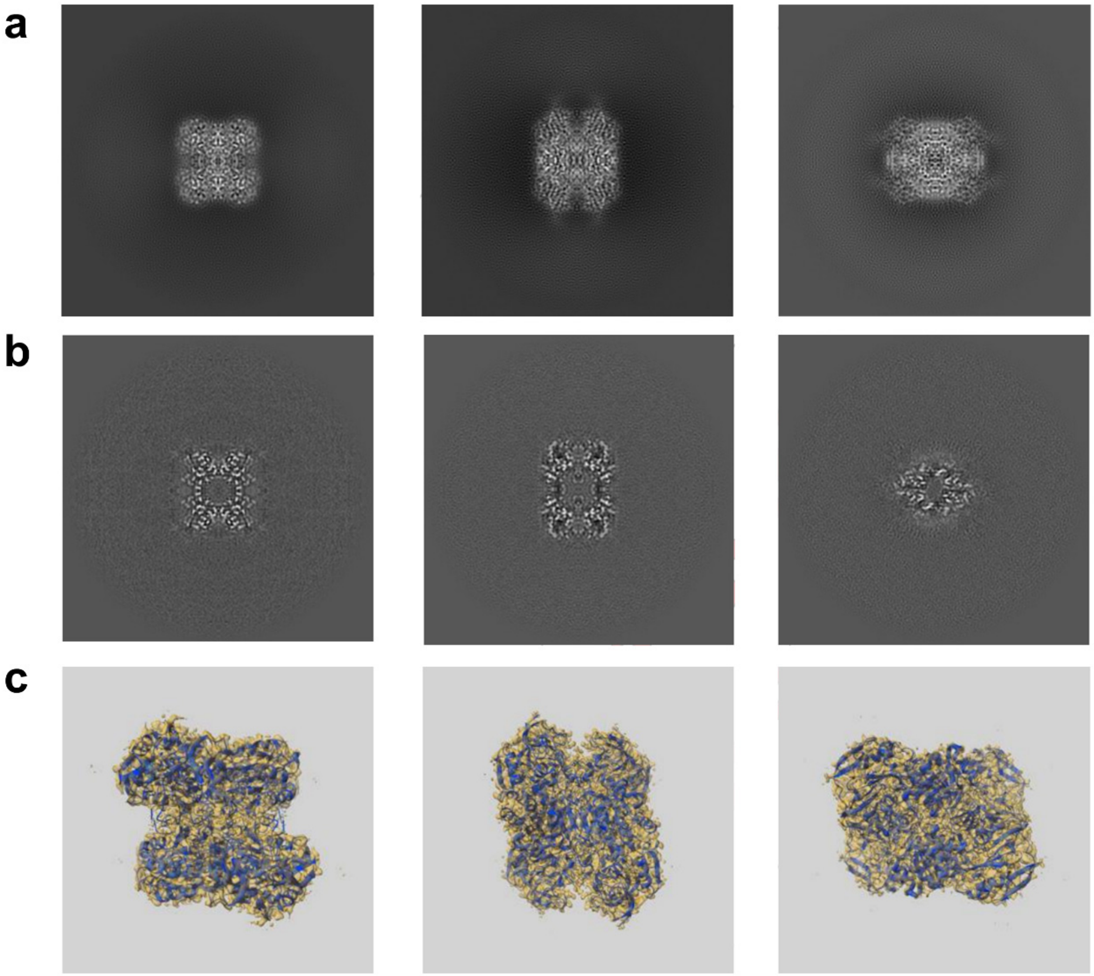

Supplementary Figure 22. Map visualization and map-model overlay. (a) Primary map projected in three orthogonal directions. (b) Central slices of (a). (c) Overlay of map and PDB model, where 3D surface view of the map at the recommended contour level 0.71 in yellow overlaid with a ribbon representation of the model colored in blue. These images demonstrated good quality of fit between the atomic model and the map.

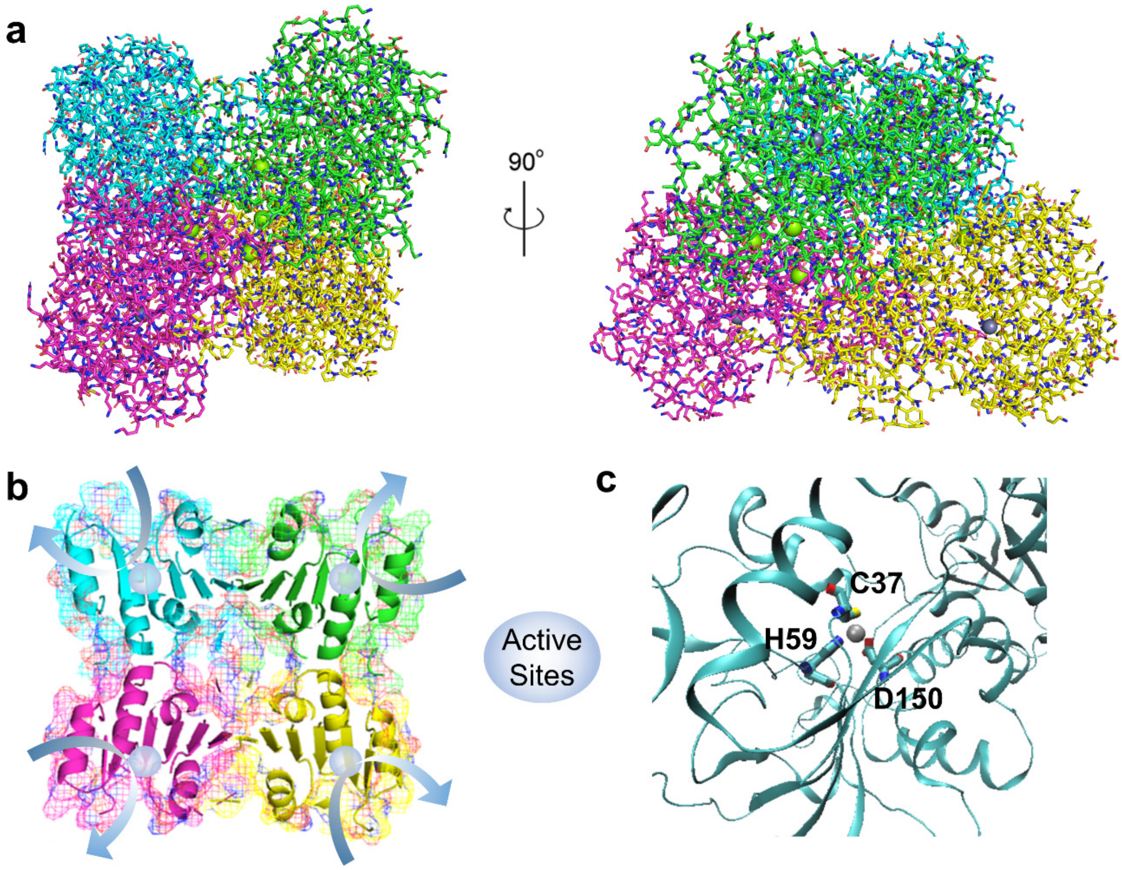

Supplementary Figure 23. (a) Overall sticks visualization of the cryo-EM structure of the TbSADH from EAG. Colored by chain. (b) The central cross section of the overlay of cartoon and mesh models, clearly showing the four catalytic active sites of this tetrameric enzyme. (c) Display of the catalytic active site. The active center is mainly composed of zinc ions (gray bead) and three amino acid residues (H59, C37, and D150).

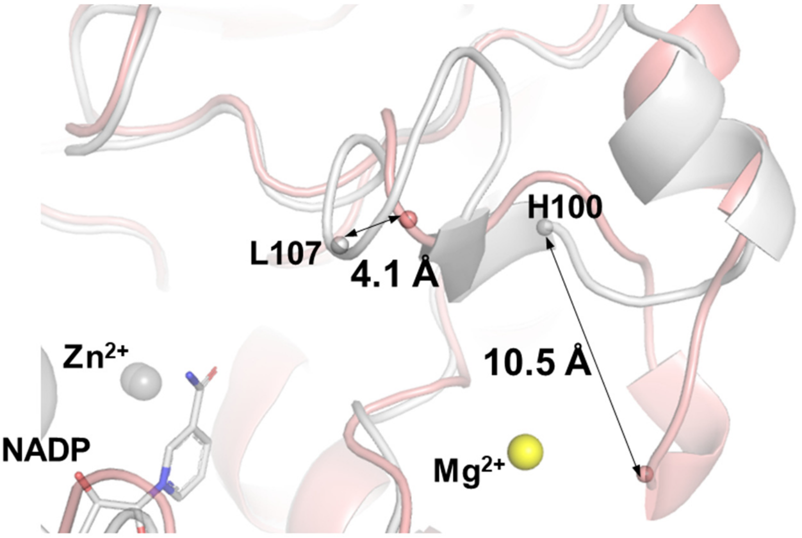

Supplementary Figure 24. The loop motion of the engineered TbSADH from EAG (salmon) compared to the wild-type TbSADH (grey). Arrows indicate the shifting of L107 and H100 with the distance of 4.1 Å and 10.5 Å, respectively.

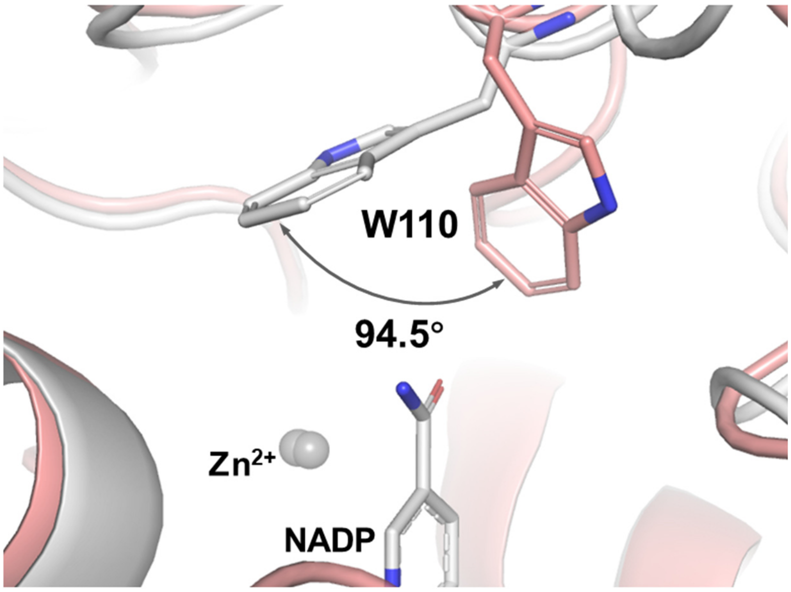

Supplementary Figure 25. The side-chain flipping of residue W110. Arrow indicates the 94.5-degree angle between the two indole planes.

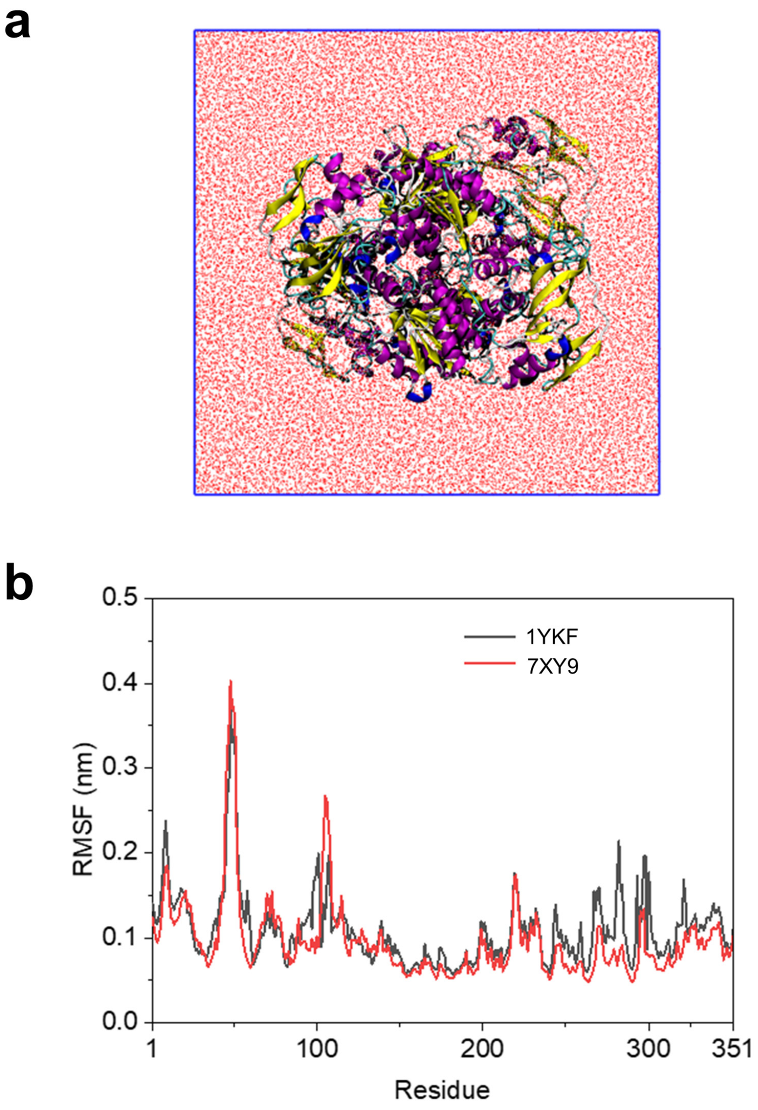

Supplementary Figure 26. (a) The systems were solvated in a cubic box with water. (b) The per-residue root mean square fluctuations (RMSF) of 1YKF and 7XY9.

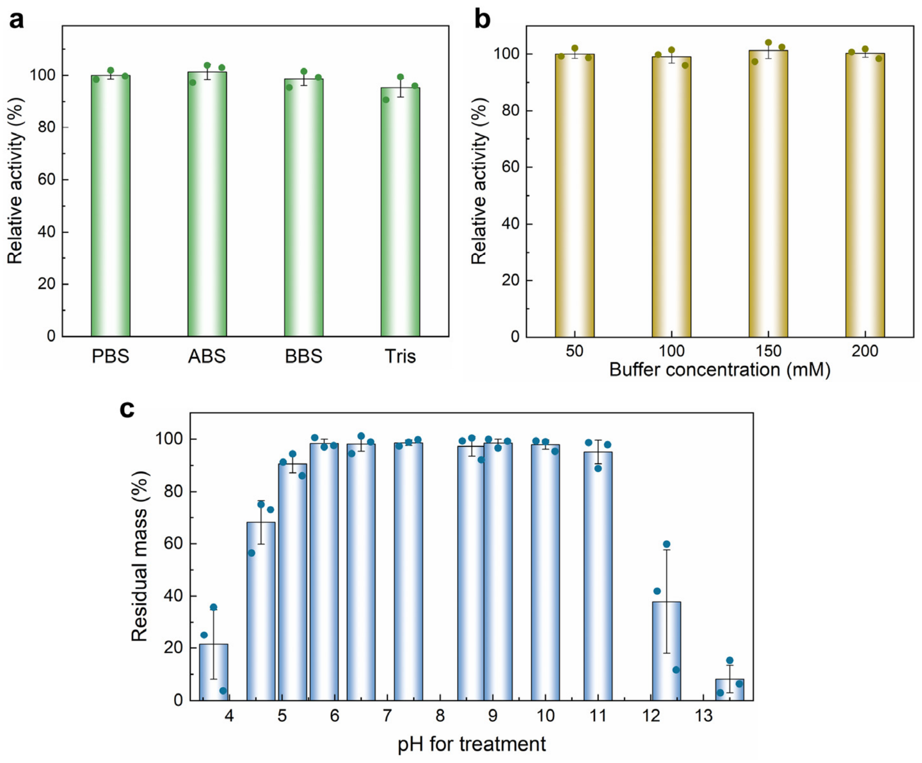

Supplementary Figure 27. Tolerance of EAG to different buffer salt conditions. (a) Comparison of the catalytic activity of the filtered gel after immersion of equal amounts of EAG in four buffer salt solutions (50 mM, pH 7.4) for 3 h, respectively. Deionized water was used for multiple cleaning during the filtrations. PBS refers to phosphate buffer solution, ABS refers to acetate buffer solution, BBS refers to borate buffer solution, and Tris refers to (tris-(hydroxymethyl)-aminomethane)-HCl buffer solution. (b) Comparison of the catalytic activity of the filtered gel after immersion in different concentrations of PBS (pH 7.4) for 3 h. The results in (a) and (b) indicate that the buffer salt type and salt concentration have a small effect on the stability of EAG. (c) Percentages of mass remaining after immersion of equal amounts of EAG in NaCl solutions (50 mM) with different pH for 30 min. The immersed EAG was obtained after filtration and washing with deionized water, and then weighed after drying. The residual mass is the percentage of weight after treatment relative to that before treatment. The results showed that EAG would be severely dissolved in the case of too acidic or too alkaline, and the suitable pH was determined to be 5.8–11. For all, n = 3. Data are expressed as the mean ± SEM. Error bars represent the standard deviations from three independent experiments.

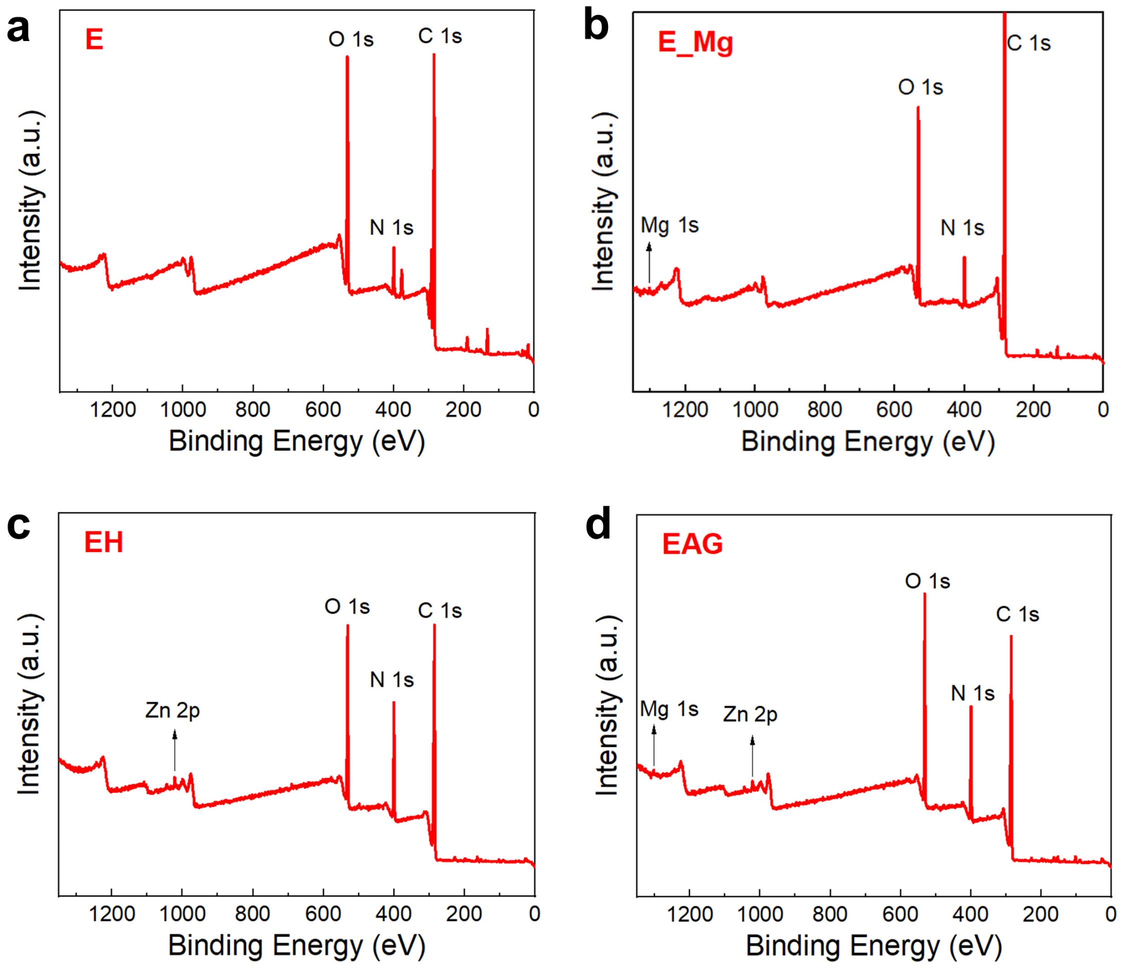

Supplementary Figure 28. XPS analysis. The survey spectrum of (a) lyophilized enzyme powder, (b) enzyme-Mg^{2+} complex, (c) enzyme-Hmtz complex generated by pre-gelation, and (d) EAG. The enhancement of N1s signal in EH and EAG revealed the existence of Hmtz. Mg1s signal in E_Mg and EAG revealed the existence of Mg^{2+}. EH and EAG exhibited Zn 2p signals, while E and E_Mg did not, indicating that the active sites of alcohol dehydrogenase in gel state were more significantly exposed.

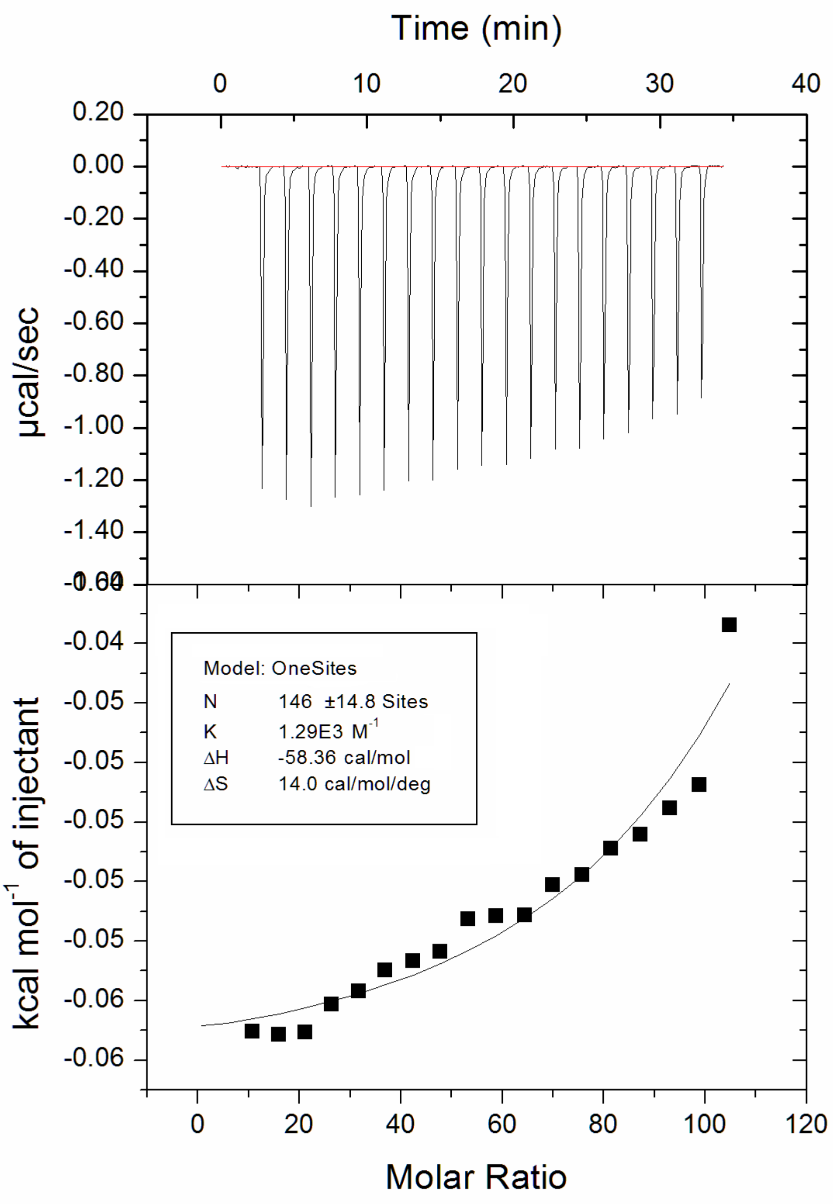

Supplementary Figure 29. Isothermal titration thermograms of Hmtz (20 mM) titrated into the solution of TbSADH (0.1 mM) in water at 25 °C. The results suggested intermolecular weak interactions between enzyme and Hmtz with a dissociation constant (K_d) of 7.7×10^{-4} M.

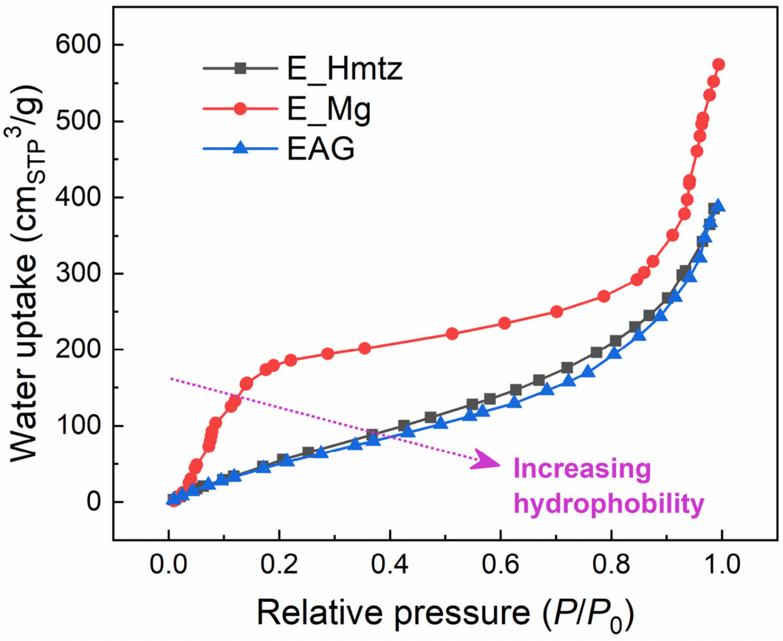

Supplementary Figure 30. Water adsorption isotherms of E_Hmtz, E_Mg, and EAG at 298 K. The lower the water uptake at a certain relative pressure, the more hydrophobic the sample is. The results revealed that the combination of Hmtz and enzyme significantly enhanced the hydrophobic effect, which may be a driving force of the gelation process. The hydrophobicity order[2] of the three solids: EAG>E_Hmtz>E_Mg.

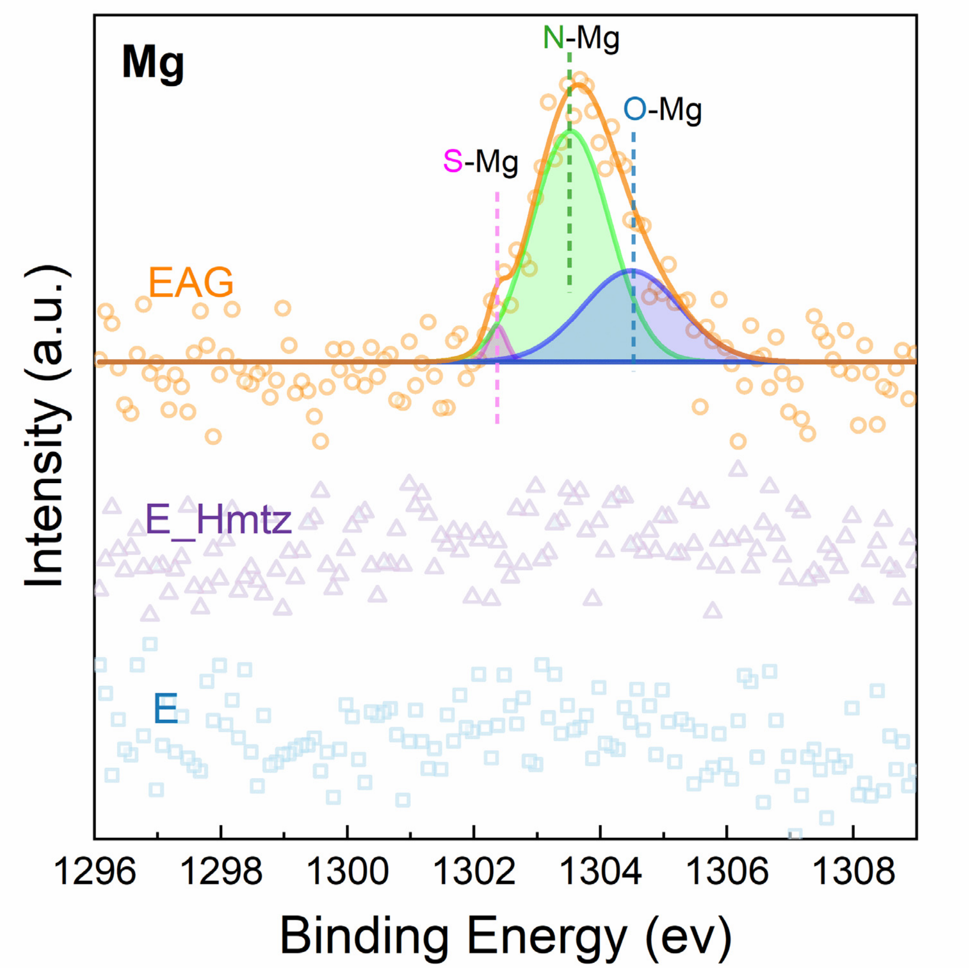

Supplementary Figure 31. High-resolution Mg 1s XPS spectra of E, E_Hmtz and EAG. The results demonstrated that there was a strong signal of Mg in EAG, which was in the prestation of divalent ions[3]. The coordination of Mg^{2+} with N, O, and S elements of the enzyme was further confirmed.

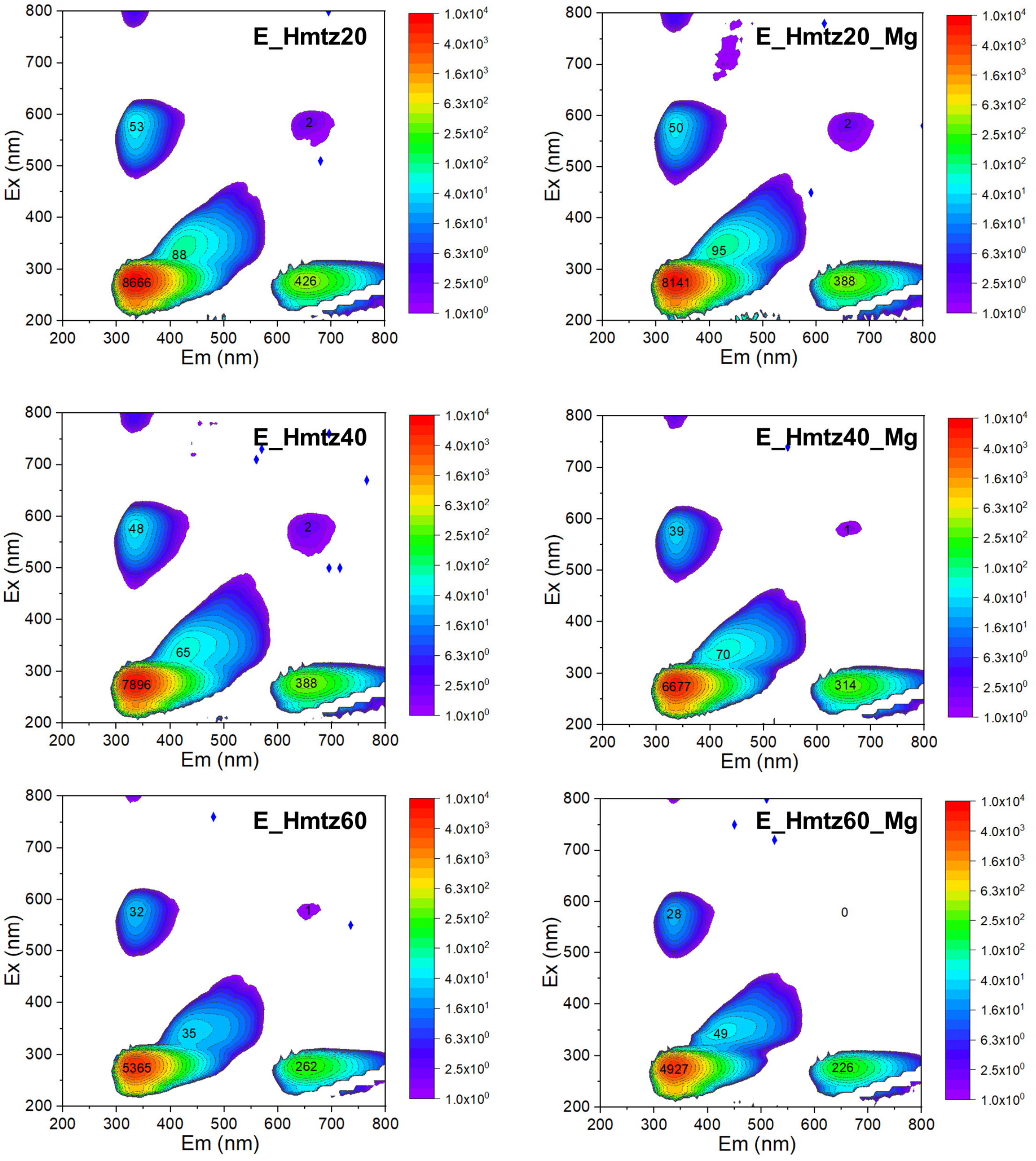

Supplementary Figure 32. Three-dimensional excitation-emission matrix (EEM) fluorescence spectra of various E_Hmtz and EAG of different Hmtz/E ratios. The main fluorescence was concentrated in (Ex, Em) = (280 nm, 335 nm), which belongs to the fluorescence characteristics of tryptophan[4]. Hence the tryptophan of the enzyme can be used as a probe to study the interaction between Hmtz or magnesium ions and the enzyme.

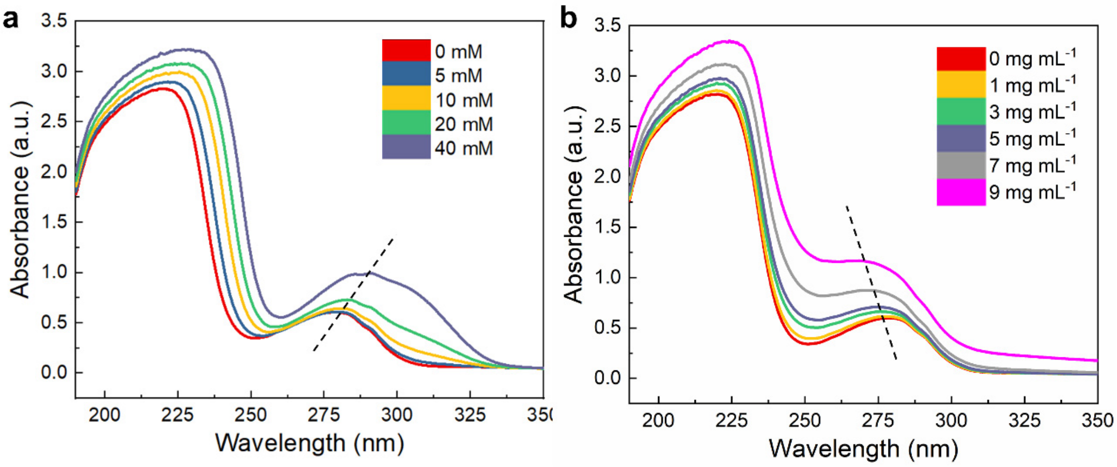

Supplementary Figure 33. The UV absorption shift caused by the addition of magnesium ions and Hmtz. (a) Absorption curves after adding different concentrations of magnesium ions (0~40 mM) to the enzyme solution. The concentrations refer to that in the mixed solution formed after adding. The maximum absorption wavelength (λ_{max}) was shifted from 278 nm to 291 nm as the concentration of Mg^{2+} increased from 0 to 40 mM, suggesting the binding affinity of the tryptophan with Mg^{2+} [5]. (b) Absorption curves after adding different concentrations of Hmtz (0~9 mg mL^{-1}) to the enzyme solution. λ_{max} was shifted from 278 nm to 268 nm as the concentration of Hmtz increased from 0 to 9 mg mL^{-1}, indicating that the microenvironmental hydrophobicity of the surface tryptophan increased[6].

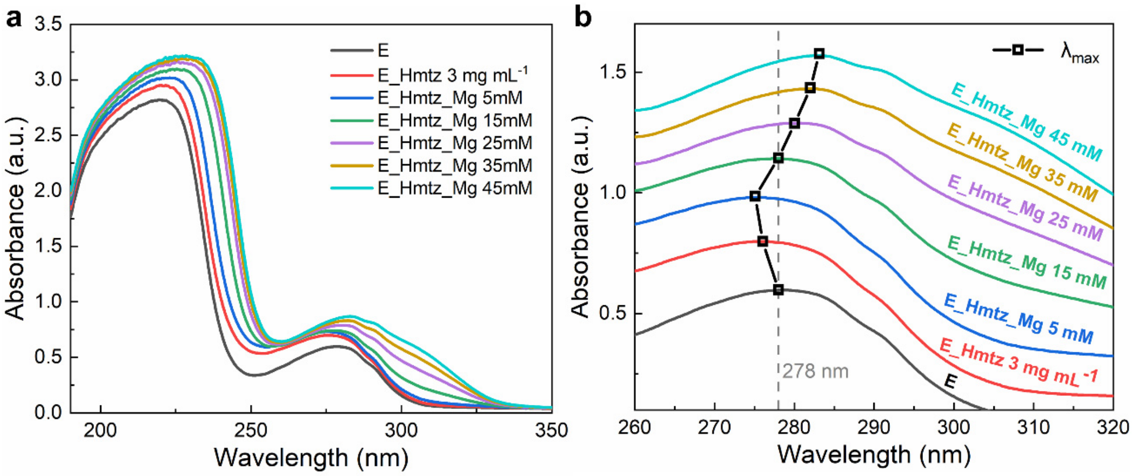

Supplementary Figure 34. The UV-vis absorption curves of each stage of adding Hmtz and then magnesium ions in the enzyme solution. (a) Wide range spectrum of 190~350 nm. (b) Fine spectrum around 278 nm. The results demonstrated that interactions between Hmtz/Mg^{2+} and enzyme were different, and the shift of λ_{max} can be corrected by appropriate proportion of Hmtz to Mg^{2+}.

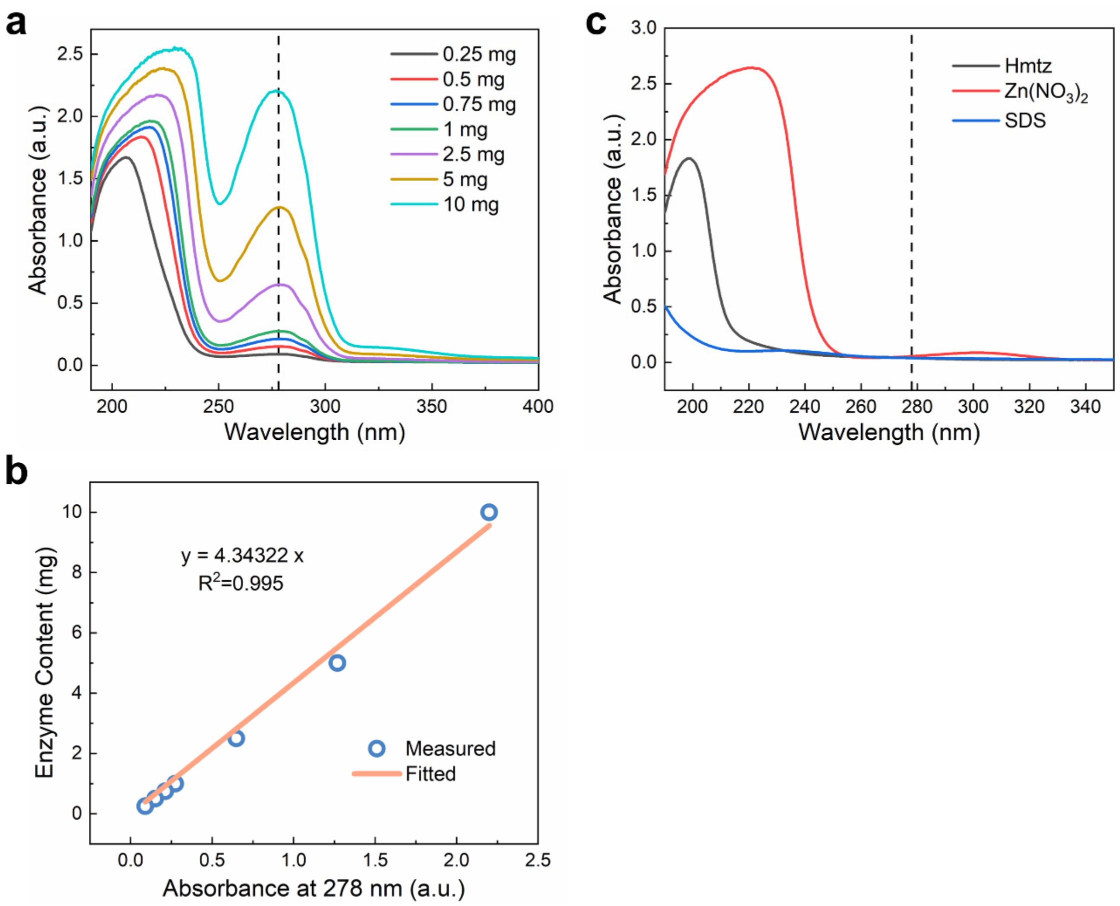

Supplementary Figure 35. Determination of enzyme content in E_Hmtz or EAG by UV-vis absorption. (a) Absorption curves of alcohol dehydrogenases at different concentrations. E_Hmtz or EAG contained a certain amount of enzyme was first dissolved in 1 mL 10% SDS, and then it was diluted to 3 mL with PBS (50 mM, pH 7.4). The test solution volume was 3 mL. (b) Calibration curve for enzyme content in E_Hmtz or EAG. (c) Absorption curves of Hmtz, $Zn(NO_3)_2$ and SDS in 50 mM PBS (pH 7.4). There is no UV absorption at 278 nm, which excludes the influence of these components on the determination of enzyme amount.

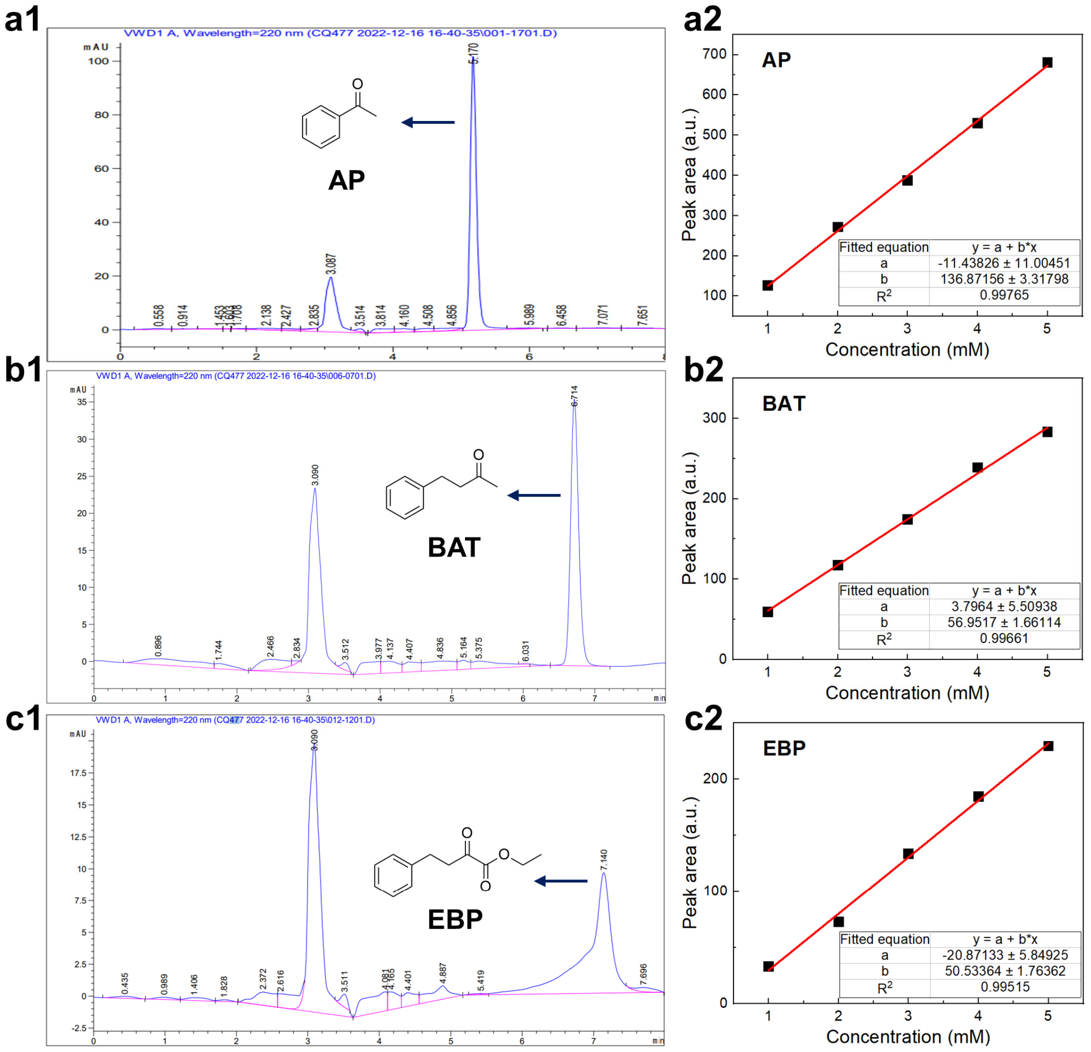

Supplementary Figure 36. Chromatographs and the standard calibration plots of the substrate AP (a), BAT (b), and EBP (c). Chromatographic condition: a Chiralcel OD-H column (4.6 mm×250 mm, 5 μm, Diacel) was used at 30°C with a detection wavelength of 220 nm, and hexane/isopropanol (92:8, v/v) was used as eluent at 1.0 mL min^{-1}.

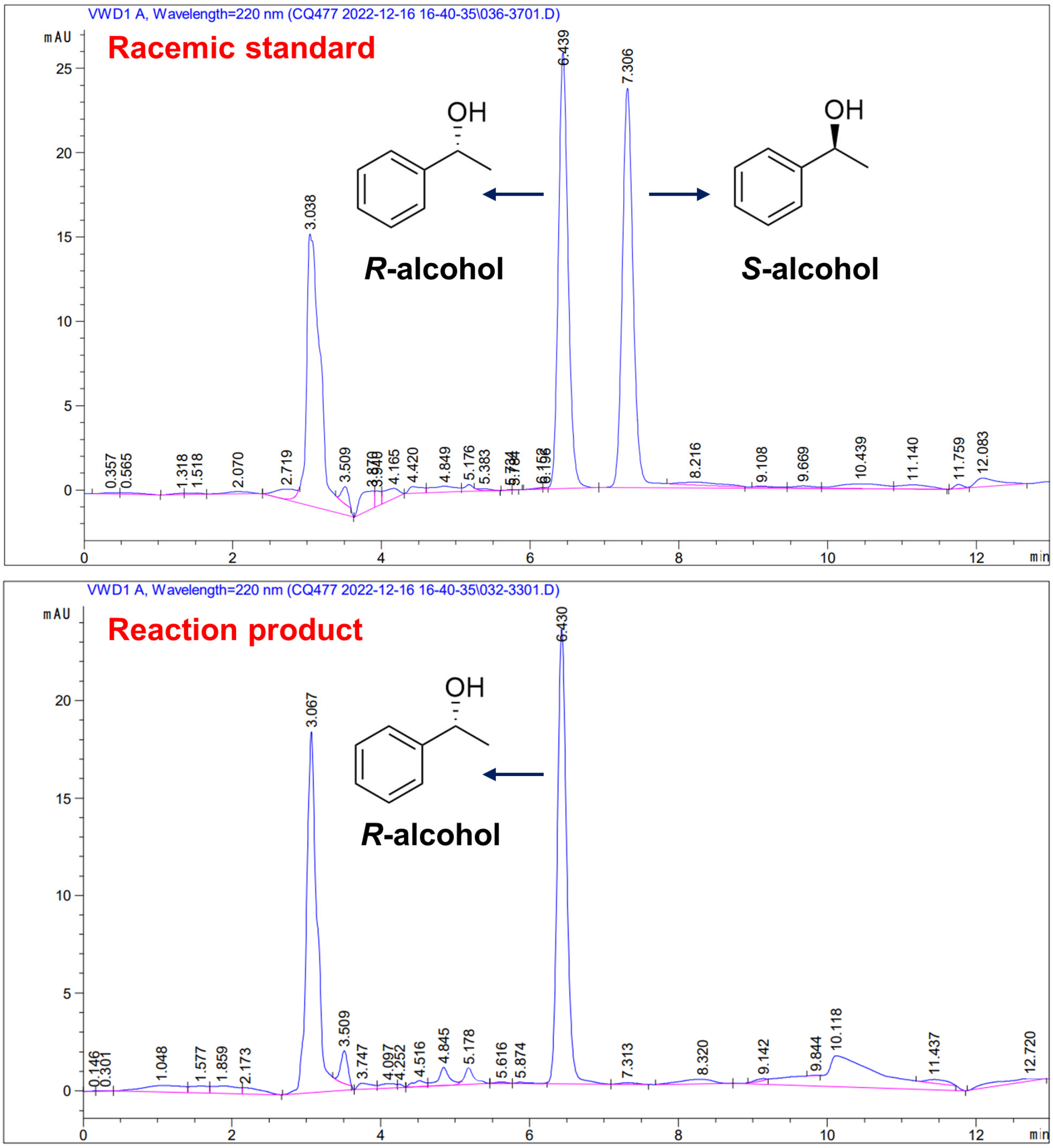

Supplementary Figure 37. Chromatographs of the standard racemic mixture of *R*- and *S*- alcohol related to AP, and the EAG-catalyzed product from the asymmetric reduction of AP. The results showed that enantiopure *R*-alcohol was obtained, with e.e. above 99%.

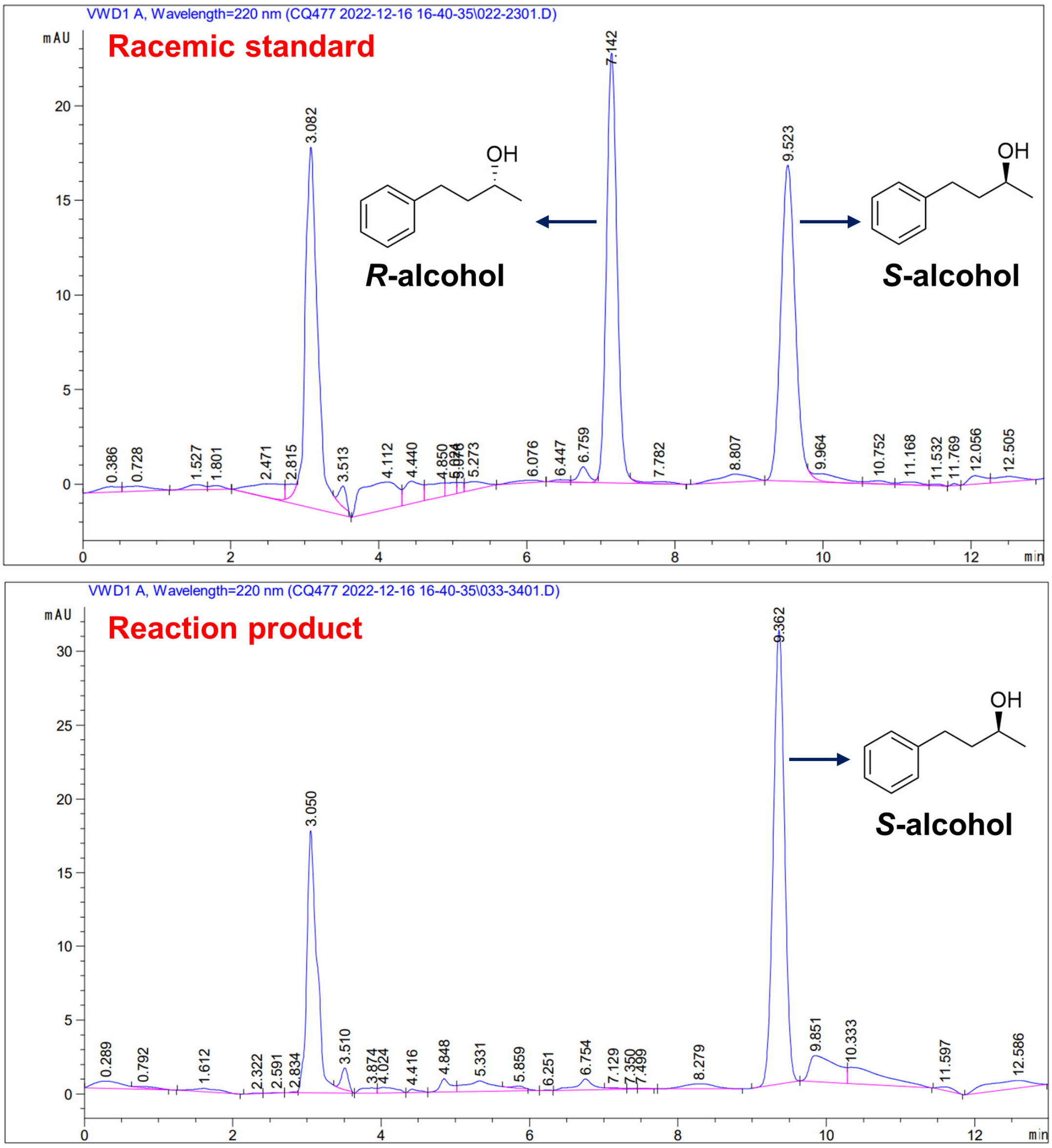

Supplementary Figure 38. Chromatographs of the standard racemic mixture of *R*- and *S*- alcohol related to BAT, and the EAG-catalyzed product from the asymmetric reduction of BAT. The results showed that enantiopure *S*-alcohol was obtained, with e.e. above 99%.

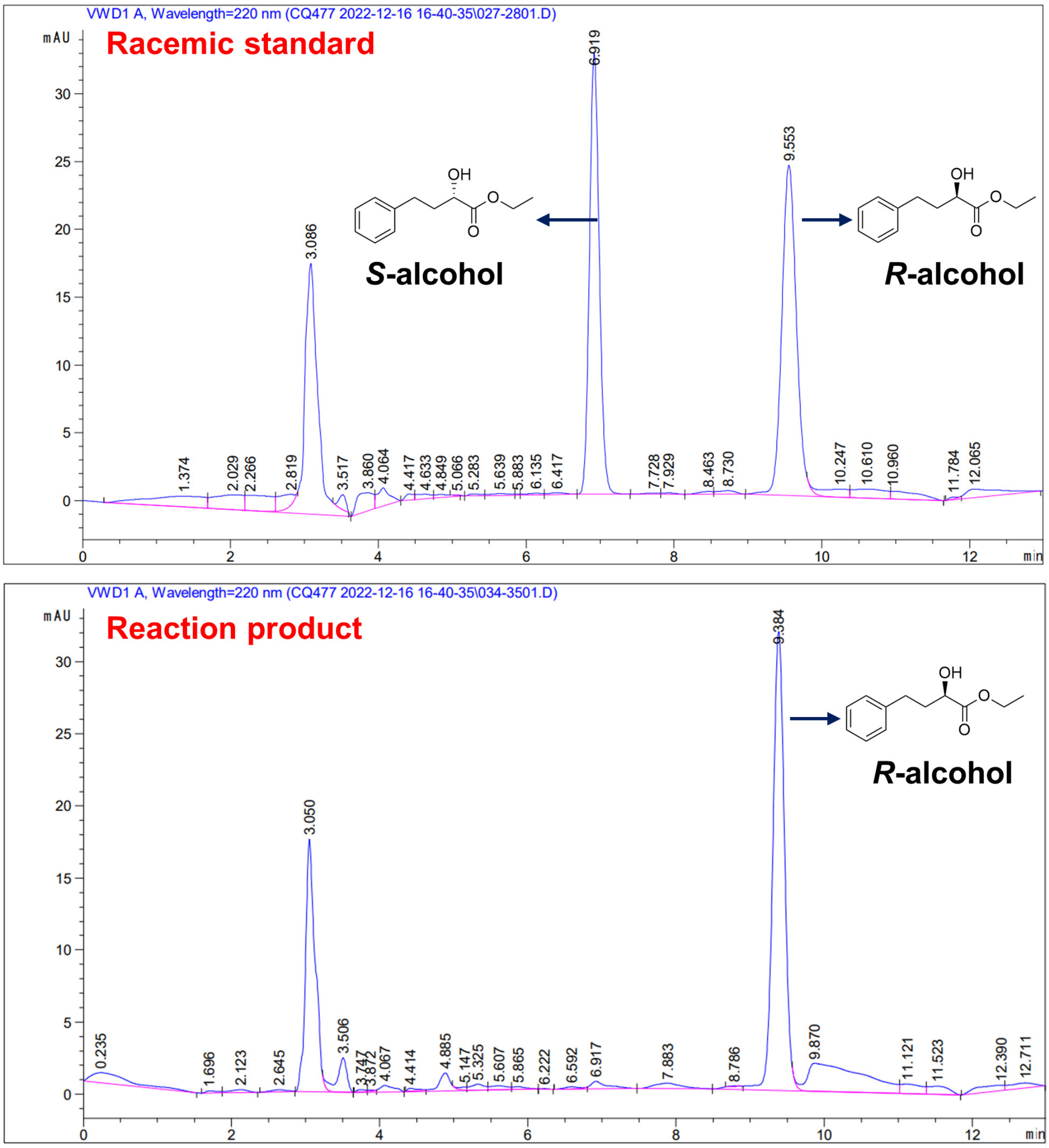

Supplementary Figure 39. Chromatographs of the standard racemic mixture of *R*- and *S*- alcohol related to EBP, and the EAG-catalyzed product from the asymmetric reduction of EBP. The results showed that enantiopure *R*-alcohol was obtained, with e.e. above 99%.

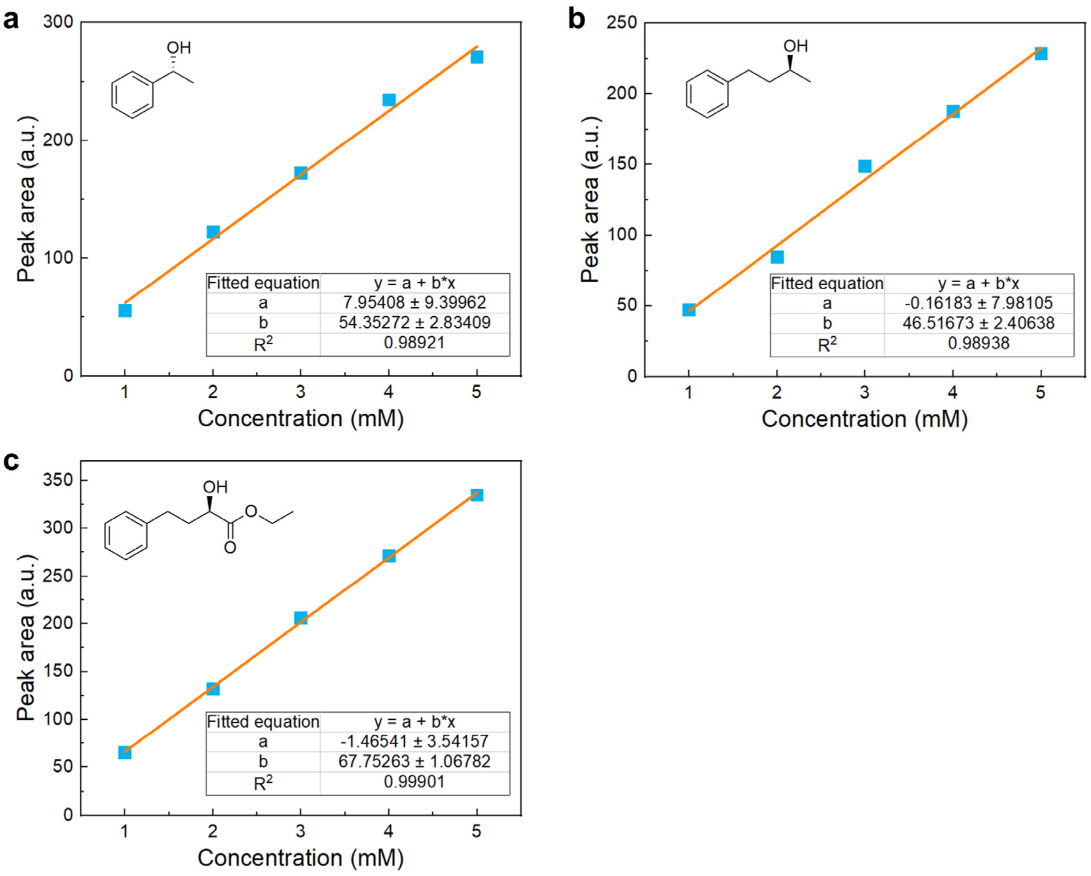

Supplementary Figure 40. Standard calibration plots of the produced enantiopure alcohol related to AP (a), BAT (b), and EBP (c). Chromatographic condition: a Chiralcel OD-H column (4.6 mm×250 mm, 5 µm, Diacel) was used at 30°C with a detection wavelength of 220 nm, and hexane/isopropanol (92:8, v/v) was used as eluent at 1.0 mL min^{-1}.

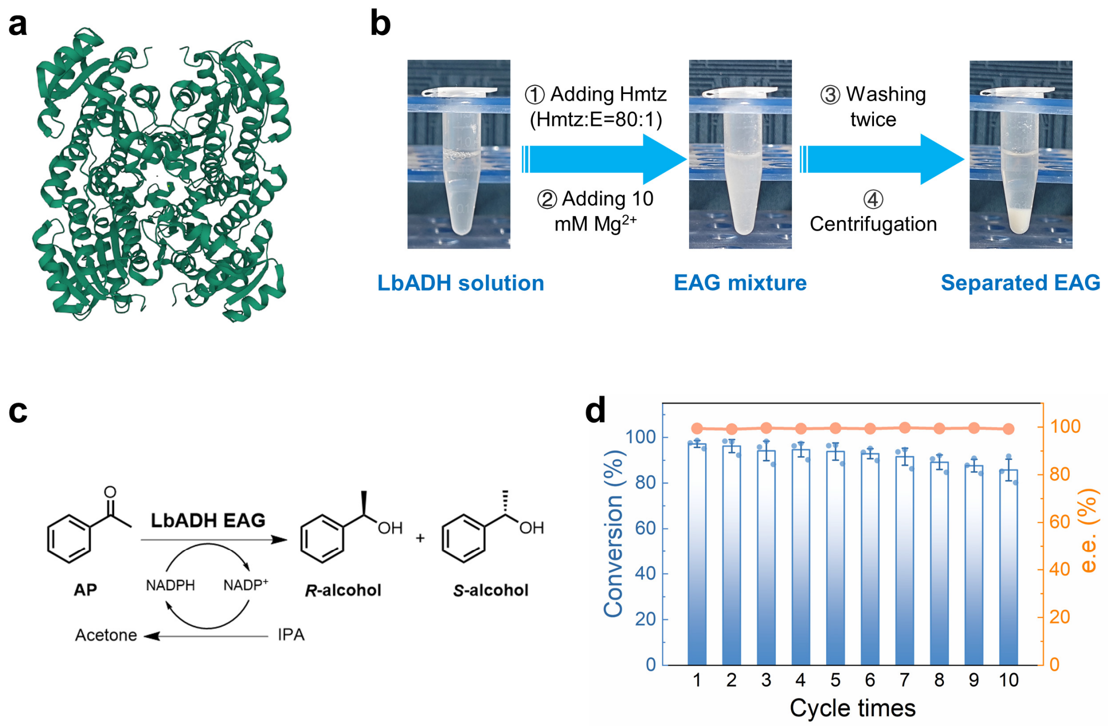

Supplementary Figure 41. Catalytic reusability test of the EAG made with LbADH (an ADH from *Levilactobacillus brevis*). (a) Overall structure of LbADH. PDB code: 1NXQ. (b) The preparation process of LbADH EAG. The complete preparation method is the same as that of TbSADH. (c) LbADH EAG-catalyzed asymmetric reduction of 5 mM AP. (d) Conversions and enantiomeric excess values measured during 10 reaction cycles using LbADH EAG as the catalyst. The results showed a chiral selectivity of >99% for the *R*-alcohol. The catalytic activity of LbADH EAG retained 88.3% of the initial activity after being reused 10 times. The determination method of the catalytic activity is the same as TbSADH EAG except that the reaction time is 20 min. n = 3. Error bars represent the standard deviations from three independent experiments. Data are expressed as the mean ± SEM.

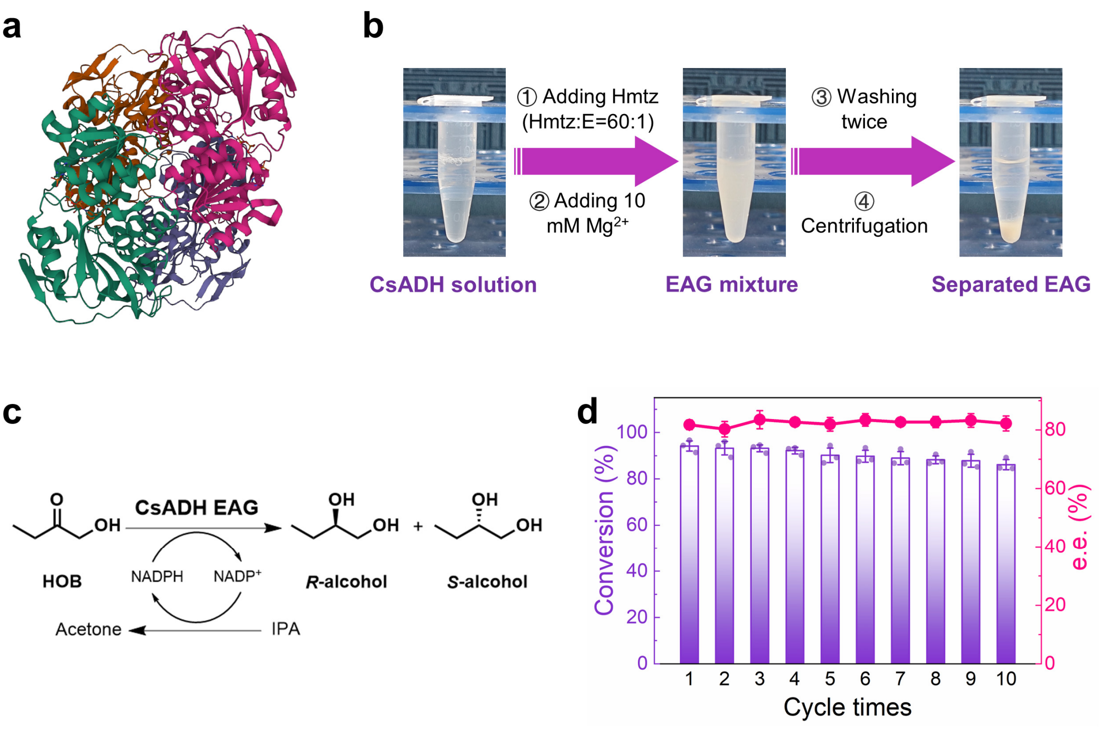

Supplementary Figure 42. Catalytic reusability test of the EAG made with CsADH (an ADH from *Caldanaerobacter subterraneus*). (a) The structure of the CsADH used has not been reported, and reference can be made to the protein structure with PDB code 7JNS. (b) The preparation process of CsADH EAG. The complete preparation method is the same as that of TbSADH. (c) CsADH EAG-catalyzed asymmetric reduction of 5 mM 1-hydroxy-2-butanon (HOB). (d) Conversions and enantiomeric excess values measured during 10 reaction cycles using CsADH EAG as the catalyst. The results showed a chiral selectivity of ~82% for the *R*-alcohol. The catalytic activity of CsADH EAG retained 91.5% of the initial activity after being reused 10 times. The determination method of the catalytic activity is the same as TbSADH EAG except that the reaction time is 20 min. $n = 3$. Error bars represent the standard deviations from three independent experiments. Data are expressed as the mean ± SEM.

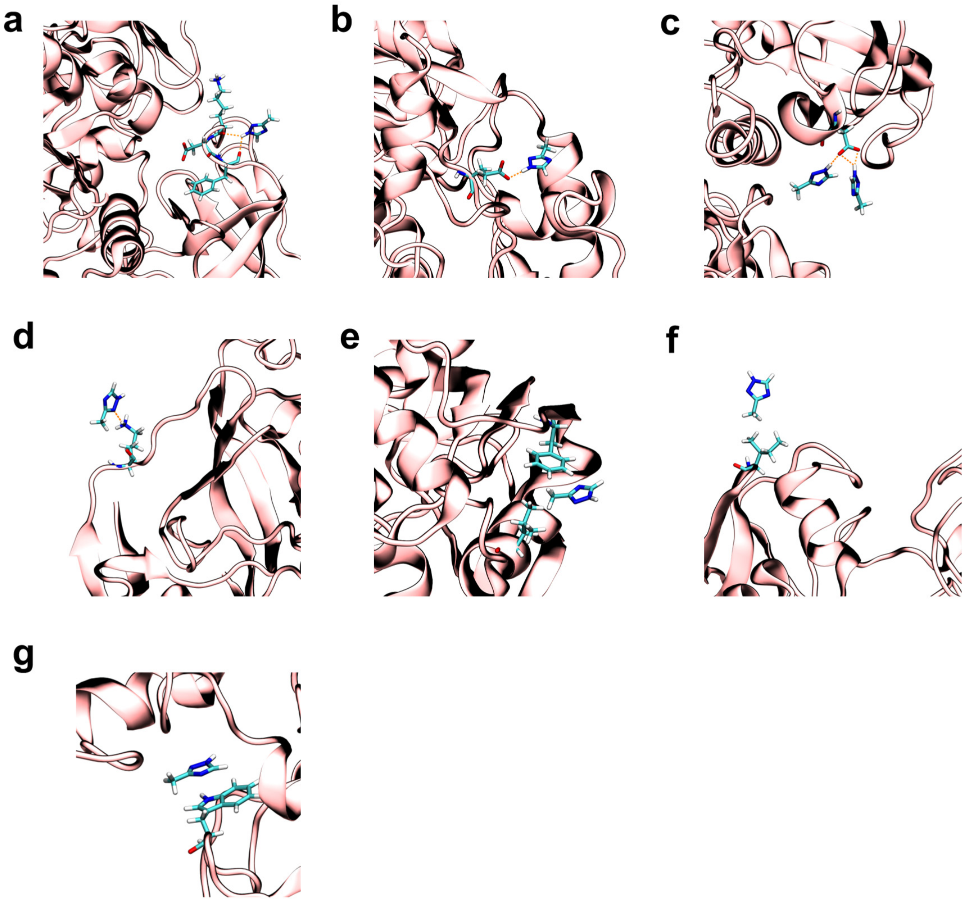

Supplementary Figure 43. Potential sites of non-covalent interaction of Hmtz on the protein surface. Potential locations include, for example, hydrogen bonding sites provided by the carbonyl oxygen of backbone or Asp/Glu residues (a–d), hydrophobic interaction sites provided by Leu/Ile residues (e–f), and π-π interaction sites provided by Trp residues (g).

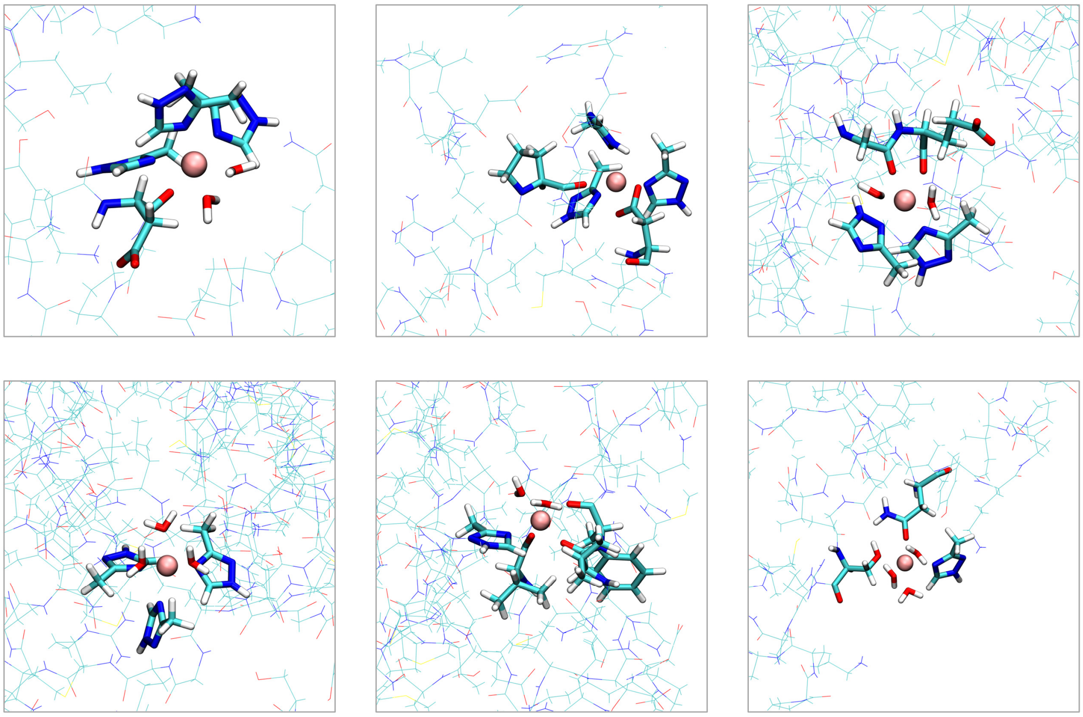

Supplementary Figure 44. Examples of local structures of magnesium ions coordinated with Hmtz and proteins based on MD simulations. The results show that magnesium ions mainly coordinated with N atoms of Hmtz, N or O atoms of backbone or amino acid residues on the protein surface, which are consistent with the XPS results (Fig. 7e and Supplementary Fig. 31).

Supplementary Tables

Supplementary Table 1. The enzymatic kinetic parameters K_m and V_{max} determined from the plots of $1/V$ against 1/[AP] (0.05 to 0.2 mM^{-1}) for the asymmetric catalytic reduction of AP with free enzyme or EAG. The relevant parameters were fitted and solved by literature method[7].

Catalyst	Free E	EAG
K_m (mM)	17.86	19.84
V_{max} ($\times 10^{-2}$, μmol s^{-1})	0.80	5.02
k_{cat} (s^{-1})	2.46	15.45
k_{cat}/K_m (s^{-1} mM^{-1})	0.14	0.78

Supplementary Table 2. Conversion of AP at 4 min catalyzed by EAG synthesized under different magnesium ion concentration ($c(Mg^{2+})$) and pre-gelation time (Δt).

Entry	$c(Mg^{2+})$ /mM	Δt /min	Conversion /%
1	0	2	49.2
2	0	5	49.7
3	0	10	49.6
4	0	15	45.8
5	0	20	46.1
6	5	2	59.8
7	5	5	66.3
8	5	10	71.2
9	5	15	49.1
10	5	20	48.3
11	10	2	54.5
12	10	5	61.3
13	10	10	72.4
14	10	15	53.0
15	10	20	49.3
16	20	2	51.0
17	20	5	58.3
18	20	10	68.2
19	20	15	55.3
20	20	20	50.8
21	50	2	45.7
22	50	5	56.6
23	50	10	64.3
24	50	15	52.2
25	50	20	53.9
26	100	2	43.1
27	100	5	47.3
28	100	10	60.4
29	100	15	43.1
30	100	20	40.8
31	200	2	26.9
32	200	5	36.2
33	200	10	41.0
34	200	15	28.0
35	200	20	27.8
36	200	2	26.9

Supplementary Table 3. Cryo-EM data collection and model statistics.

**Data collection**	
EM equipment	FEI Titan Krios
Voltage (kV)	300
Detector	Gatan K3 (6k × 4k)
Pixel size (Å)	0.8374
Electron dose (e^-/Å^2)	50
Defocus range (μm)	-0.7 ~ -1.6
Number of collected micrographs	8299
Number of selected micrographs	7064
Frames per micrograph	32
**Reconstruction**	
Software	RELION
Number of used Particles	1254312
Symmetry	D2
Map resolution (Å)	2.12
Map sharpening B-factor (Å^2)	90.4
**Refinement**	
Software	Phenix & Coot
FSC threshold	0.143
Model composition	
Protein residues	1376
Metal	4 Zn & 8 Mg
R.m.s.deviations	
Bonds length (Å)	0/10608
Bonds Angle (˚)	0/14344
Ramachandran plot statistics (%)	
Preferred	96
Allowed	4
Outlier	0

Supplementary Table 4. Conditions for chiral HPLC analysis and retention time of each substance.

Reaction	Substance	Procedure	Retention time /min
AP reduction	AP	Chiralcel OD-H column (4.6 mm × 250 mm, 5 μm, Diacel); 30 °C; 220 nm; *n*-hexane: IPA (92:8, v/v), 1 mL min^{-1}	5.2
	R-alcohol		6.4
	S-alcohol		7.3
BAT reduction	BAT		6.7
	R-alcohol		7.1
	S-alcohol		9.5
EBP reduction	EBP		7.1
	S-alcohol		6.9
	R-alcohol		9.5

Enzyme Sequences

>TbSADH SZ2205 (GeneBank: WP_041589967.1, an ADH from *Thermoanaerobacter*) in pET22b vector

MHHHHHHKGFAMLSIGKVGWIEKEKPAPGPFDAIVRPLAVAPCTSDIHTVFE
GAIGERHNMILGHEAVGEVVEVGSEVKDFKPGDRVVVPANTPDWRTSEVQR
GYHQHSGGMLAGWKFSNVKDGVFGEFFHVNDADMNLAHLPKEIPLEAAVM
IPDMMTTGFHGAELADIELGATVAVLGIGPVGLMAVAGAKLRGAGRIIAVGS
RPVCVDAAKYYGATDIVNYKDGPIESQIMNLTEGKGVDAAIIAGGNADIMAT
AVKIVKPGGTIANVNYFGEGEVLPVPRLEWGCGMAHKTIKGGLCPGGRLRM
ERLIDLVFYKRVDPSKLVTHVFRGFDNIEKAFMLMKDKPKDLIKPVVILA*

>LbADH (GeneBank: CAD66648.1, an ADH from *Levilactobacillus brevis*) in pET28a vector

MGSSHHHHHHSSGLVPRGSHMSNRLDGKVAIITGGTLGIGLAIATKFVEEGAK
VMITGRHSDVGEKAAKSVGTPDQIQFFQHDSSDEDGWTKLFDATEKAFGPVS
TLVNNAGIAVNKSVEETTTAEWRKLLAVNLDGVFFGTRLGIQRMKNKGLGAS
IINMSSIEGFVGDPSLGAYNASKGAVRIMSKSAALDCALKDYDVRVNTVHPGY
IKTPLVDDLPGAEEAMSQRTKTPMGHIGEPNDIAYICVYLASNESKFATGSEFV
VDGGYTAQ*

>CsADH (GeneBank: WP_003868131.1, an ADH from *Caldanaerobacter subterraneus*) in pET28a vector

MGMKGFAMLSIGKVGWIEKEKPTPGPFDAIVRPLAVAPCTSDIHTVFEGAIGE
RHNMILGHEAVGEVVEVGSEVKDFKPGDRVVVPAITPDWRTSEVQRGYHQHS
GGMLAGWKFSNIKDGVFGEFFHVNDADMNLAHLPKEIPLEAAVMIPDMMTT
GFHGAELAEIELGASVAVLGIGPVGLMAVAGAKLRGAGRIIVVGSRPVCVDAA
KYYGATDIVNYKNGPIDSQIMDLTEGKGVDAAIIAGGNADIMATAVKIVKPGG
TIANVNYFGEGDVLPVPRLEWGCGMAHKTIKGGLCPGGRLRMERLINLVFYK
RVDPSKLVTHVFQGFDNIEKALMLMKDKPKDLIKPVVILTLEHHHHHH*

Supplementary References

1. Sun ZT, Li GY, Ilie A, Reetz MT. Exploring the substrate scope of mutants derived from the robust alcohol dehydrogenase TbSADH. *Tetrahedron Lett* **57**, 3648-3651 (2016).
2. Li YM*, et al.* Fine-Tuning the Micro-Environment to Optimize the Catalytic Activity of Enzymes Immobilized in Multivariate Metal-Organic Frameworks. *Journal of the American Chemical Society* **143**, 15378-15390 (2021).
3. Khairallah F, Glisenti A, Galenda A, Natile MM. Mixed Magnesium and Zinc Oxide Prepared by Co-precipitation and Analyzed by XPS. *Surf Sci Spectra (USA)* **19**, 13-22 (2012).
4. Hudson N*, et al.* Can fluorescence spectrometry be used as a surrogate for the Biochemical Oxygen Demand (BOD) test in water quality assessment? An example from South West England. *Science of the Total Environment* **391**, 149-158 (2008).
5. Kim D-Y, Shinde S, Ghodake G. Colorimetric detection of magnesium (II) ions using tryptophan functionalized gold nanoparticles. *Sci Rep* **7**, (2017).
6. Li XY*, et al.* Cooperative catalysis by a single-atom enzyme-metal complex. *Nat Commun* **13**, (2022).
7. Chen WH, Vazquez-Gonzalez M, Zoabi A, Abu-Reziq R, Willner I. Biocatalytic cascades driven by enzymes encapsulated in metal-organic framework nanoparticles. *Nat Catal* **1**, 689-695 (2018).
